# Supplementary material for: Multivariate analysis reveals shared genetic architecture of brain morphology and human behavior
Source: Commun Biol. 2021 Oct 12;4:1180. doi: 10.1038/s42003-021-02712-y (PMC8511103; doi:10.1038/s42003-021-02712-y)
Supplement: Supplementary file 2 — Supplementary Information [file 42003_2021_2712_MOESM2_ESM.pdf]

Supplementary Information: Multivariate analysis reveals shared genetic architecture of brain morphology and human behavior

Ronald de Vlaming[1,*], Eric A.W. Slob[2,3,4,*], Philip R. Jansen[5,6], Alain Dagher[7], Philipp D. Koellinger[1,8], Patrick J.F. Groenen[9], and Cornelius A. Rietveld[2,3,*]

[1]School of Business and Economics, Vrije Universiteit Amsterdam, Amsterdam, The Netherlands

[2]Department of Applied Economics, Erasmus School of Economics, Rotterdam, The Netherlands

[3]Erasmus University Rotterdam Institute for Behavior and Biology, Erasmus School of Economics, Rotterdam, The Netherlands

[4]MRC Biostatistics Unit, School of Clinical Medicine, University of Cambridge, Cambridge, UK

[5]Amsterdam Neuroscience, Vrije Universiteit Amsterdam, Amsterdam, The Netherlands

[6]Department of Complex Trait Genetics, Center for Neuroscience and Cognitive Research, Amsterdam Neuroscience, Vrije Universiteit Amsterdam, Amsterdam, The Netherlands

[7]Montreal Neurological Institute, McGill University, Montreal, Quebec, Canada

[8]La Follette School of Public Affairs, University of Wisconsin-Madison, WI, USA

[9]Econometric Institute, Erasmus School of Economics, Rotterdam, The Netherlands

[*]These authors contributed equally

[**]Corresponding author: Cornelius A. Rietveld, Erasmus University Rotterdam, Burgemeester Oudlaan 50, 3062 PA, Rotterdam, The Netherlands, Phone: +31(0)10-408-1401, E-mail: `nrietveld@ese.eur.nl`

Supplementary Note 1

Genomic-relatedness-based restricted maximum likelihood (GREML) estimation, as developed and introduced by Yang et al. (2010), quantifies the degree to which genetic similarity between individuals maps to phenotypic similarity. Bivariate GREML has been developed to additionally estimate genetic correlations between combinations of two traits (Lee et al., 2012). Although tools such as MTG2 (Lee and Van der Werf, 2016) and GEMMA (Zhou and Stephens, 2012) provide multivariate generalisations of GREML, these tools offer only limited scalability in terms of the number of traits (for a detailed comparison, see Supplementary Note 3). We develop a computational efficient multivariate version of GREML, MGREML, which can be used to analyse a large number of quantitative traits simultaneously. The derivations presented in this section reflect the implementation of our method in Python 3.x (available via `https://github.com/devlaming/mgreml`).

A maximum likelihood approach is taken to jointly estimate the genetic and environmental covariance between multiple traits. In this section, efficient expressions are presented that are fundamental to make MGREML estimation computationally feasible for large data sets. Most importantly, a combination of a canonical transformation (as also proposed by Lee and Van der Werf (2016)), and a transformation using a commutation matrix are used to transform the full covariance matrix across traits and observations into a block-diagonal matrix.

As an optimisation method, we employ the quasi-Newton approach of the Broyden–Fletcher–Goldfarb–Shanno (BFGS) algorithm (see e.g., Nocedal and Wright, 2006). For this algorithm, computationally efficient expressions are needed for the log-likelihood and the gradient. In addition, to compute standard errors of our estimates, we need efficient expressions for the average information (AI) matrix (Gilmour et al., 1995).

We take the following steps to obtain an implementation of MGREML that can perform BFGS steps in $O\left(NT^2\right)$ time and calculate the standard errors of heritabilities and genetic correlations in $O\left(NT^4\right)$ time, where N denotes the sample size and T the number of phenotypes included in the analysis:

1. Set up the multivariate model used in MGREML.

2. Transform phenotypes using eigenvectors from the genomic-relatedness matrix (GRM) $\mathbf{A}$, where $\mathbf{A}$ is derived from single-nucleotide polymorphism (SNP) data, and re-order the observations, in such a way that the grand phenotypic covariance matrix across all traits and observations is block-diagonal.

3. Define the log-likelihood function, its gradient, and the AI matrix.

4. Develop tractable notation for different covariates across traits.

5. Find efficient expressions to calculate the log-likelihood.

6. Parametrise the genetic variance matrix $\mathbf{V}_G$ and environmental variance matrix $\mathbf{V}_E$ such that they are both guaranteed to be at least positive semidefinite.

7. Given the parametrisation, find efficient expressions to calculate the gradient.

8. Given the parametrisation, find efficient expressions to calculate the AI matrix.

9. Maximise the log-likelihood using a BFGS algorithm.

10. Use a delta method to compute standard errors of estimated heritabilities, genetic correlations, and environmental correlations.

In the next subsections, we discuss these steps one by one in more detail. Throughout the derivations, we will make use of the fundamental property of linear combinations of multivariate normally distributed vectors, that is, if vector $\boldsymbol{\delta} \sim \mathcal{N}(\boldsymbol{\mu}, \boldsymbol{\Sigma})$, then the linear combination $\mathbf{C}\boldsymbol{\delta} + \mathbf{m} \sim \mathcal{N}(\mathbf{C}\boldsymbol{\mu} + \mathbf{m}, \mathbf{C}\boldsymbol{\Sigma}\mathbf{C}^\top)$.

Step 1. Setting up the multivariate model

The original model for bivariate GREML, as developed by Lee et al. (2012), for two normally distributed quantitative traits, Y_1 and Y_2, observed in the same set of N unrelated individuals, can be written as follows:

$$\begin{pmatrix} \mathbf{y}_1 \\ \mathbf{y}_2 \end{pmatrix} \sim \mathcal{N}\left(\begin{pmatrix} \mathbf{X}_1 & \mathbf{0} \\ \mathbf{0} & \mathbf{X}_2 \end{pmatrix} \begin{pmatrix} \boldsymbol{\beta}_1 \\ \boldsymbol{\beta}_2 \end{pmatrix}, \begin{pmatrix} \sigma_{G_{11}}\mathbf{A} & \sigma_{G_{12}}\mathbf{A} \\ \sigma_{G_{12}}\mathbf{A} & \sigma_{G_{22}}\mathbf{A} \end{pmatrix} + \begin{pmatrix} \sigma_{E_{11}}\mathbf{I}_N & \sigma_{E_{12}}\mathbf{I}_N \\ \sigma_{E_{12}}\mathbf{I}_N & \sigma_{E_{22}}\mathbf{I}_N \end{pmatrix} \right), \tag{1}$$

where parameter $\sigma_{G_{11}}$ (resp. $\sigma_{G_{22}}$) denotes the genetic variance of trait Y_1 (Y_2) and $\sigma_{G_{12}}$ denotes the genetic covariance of traits Y_1 and Y_2. The parameters with subscript E are defined in an analogous manner for the environmental variances and covariance. Matrix $\mathbf{A}$ denotes the $N \times N$ genomic-relatedness matrix (GRM) and matrix $\mathbf{I}_N$ denotes the $N \times N$ identity matrix.. Vector $\mathbf{y}_1$ (resp. $\mathbf{y}_2$) denotes the $N \times 1$ vector of outcomes for trait Y_1 (Y_2), and matrix $\mathbf{X}_1$ (resp. $\mathbf{X}_2$) denotes the fixed-effect covariates with effects $\boldsymbol{\beta}_1$ ($\boldsymbol{\beta}_2$) for Y_1 (Y_2).

Eq. 1 can be written more compactly using the Kronecker product (denoted by $\otimes$) and by applying the vectorisation operator (denoted by $\operatorname{vec}()$, where $\operatorname{vec}([\mathbf{v}_1 \quad \dots \quad \mathbf{v}_b]) = \begin{bmatrix} \mathbf{v}_1^\top & \dots & \mathbf{v}_b^\top \end{bmatrix}^\top$) to the $N \times 2$

matrix of phenotypes, $\mathbf{Y}^*$. That is:

$$\text{vec}\left(\mathbf{Y}^*\right) \sim \mathcal{N}\left(\mathbf{X}^*\boldsymbol{\beta}, \mathbf{V}_G \otimes \mathbf{A} + \mathbf{V}_E \otimes \mathbf{I}_N\right), \text{ where}$$

$$\mathbf{Y}^* = \begin{pmatrix} \mathbf{y}_1^* & \mathbf{y}_2^* \end{pmatrix}, \quad \mathbf{X}^* = \begin{pmatrix} \mathbf{X}_1^* & \mathbf{0} \\ \mathbf{0} & \mathbf{X}_2^* \end{pmatrix}, \quad \boldsymbol{\beta} = \begin{pmatrix} \boldsymbol{\beta}_1 \\ \boldsymbol{\beta}_2 \end{pmatrix},$$

$$\mathbf{V}_G = \begin{pmatrix} \sigma_{G_{11}} & \sigma_{G_{12}} \\ \sigma_{G_{12}} & \sigma_{G_{22}} \end{pmatrix}, \text{ and } \mathbf{V}_E = \begin{pmatrix} \sigma_{E_{11}} & \sigma_{E_{12}} \\ \sigma_{E_{12}} & \sigma_{E_{22}} \end{pmatrix}.$$

Here, $\mathbf{V}_G$ is the 2×2 genetic covariance matrix and $\mathbf{V}_E$ the 2×2 environmental covariance matrix. This model can easily be generalised to a model for T normally distributed quantitative traits, all observed in the same set of N individuals, in an $N \times T$ matrix $\mathbf{Y}^*$. That is:

$$\text{vec}\left(\mathbf{Y}^*\right) \sim \mathcal{N}\left(\mathbf{X}^*\boldsymbol{\beta}, \mathbf{V}^*\right), \text{ where } \mathbf{V}^* = \mathbf{V}_G \otimes \mathbf{A} + \mathbf{V}_E \otimes \mathbf{I}_N, \tag{2}$$

and where $\mathbf{X}^*$ is a block-diagonal matrix, comprising blocks $\mathbf{X}_t^*$ for traits $t = 1, \ldots, T$, where $\mathbf{X}_t^*$ is the $N \times K_t$ matrix of fixed-effects covariates for trait t, where K_t denotes the number of fixed-effect covariates that apply to trait t. In this model, $\mathbf{V}_G$ denotes the $T \times T$ genetic covariance matrix across the T traits and $\mathbf{V}_E$ the environmental covariance matrix.

Step 2. Transforming and re-ordering phenotypes

Using a canonical transformation (see, e.g., Ducrocq and Chapuis (1997)), such that we transform the data to be independent across individuals (rather than across traits), as suggested and applied by Lee and Van der Werf (2016), we introduce a high degree of sparsity in our model. That is, by taking the eigenvalue decomposition (EVD) of $\mathbf{A} = \mathbf{PDP}^\top$, where $\mathbf{P}$ is an orthogonal matrix (i.e., such that $\mathbf{P}^\top\mathbf{P} = \mathbf{PP}^\top = \mathbf{I}_N$, with $\mathbf{I}_N$ again being the identity matrix) and $\mathbf{D}$ a diagonal matrix of eigenvalues (EVs), $d_1, \ldots, d_N$, we can

premultiply $\mathbf{Y}^*$ in Eq. 2 by $\mathbf{P}^\top$ to obtain a sparse formulation of the model. Specifically, we have that:

$$\text{vec}\left(\mathbf{P}^\top\mathbf{Y}^*\right) = \left(\mathbf{I}_T \otimes \mathbf{P}^\top\right)\text{vec}\left(\mathbf{Y}^*\right) \sim \mathcal{N}\left(\left(\mathbf{I}_T \otimes \mathbf{P}^\top\right)\mathbf{X}^*\boldsymbol{\beta}, \left(\mathbf{I}_T \otimes \mathbf{P}^\top\right)\mathbf{V}^*\left(\mathbf{I}_T \otimes \mathbf{P}\right)\right), \text{ where} \tag{3}$$

$$\left(\mathbf{I}_T \otimes \mathbf{P}^\top\right)\mathbf{V}^*\left(\mathbf{I}_T \otimes \mathbf{P}\right) = \mathbf{V}_G \otimes \left(\mathbf{P}^\top\mathbf{A}\mathbf{P}\right) + \mathbf{V}_E \otimes \left(\mathbf{P}^\top\mathbf{P}\right) \tag{4}$$

$$= \mathbf{V}_G \otimes \left(\mathbf{P}^\top\mathbf{P}\mathbf{D}\mathbf{P}^\top\mathbf{P}\right) + \mathbf{V}_E \otimes \left(\mathbf{P}^\top\mathbf{P}\right) \tag{5}$$

$$= \mathbf{V}_G \otimes \mathbf{D} + \mathbf{V}_E \otimes \mathbf{I}_N. \tag{6}$$

The distribution of $\text{vec}\left(\mathbf{P}^\top\mathbf{Y}^*\right)$ can now be written more compactly as:

$$\text{vec}\left(\mathbf{P}^\top\mathbf{Y}^*\right) \sim \mathcal{N}\left(\left(\mathbf{I}_T \otimes \mathbf{P}^\top\right)\mathbf{X}^*\boldsymbol{\beta}, \mathbf{V}_G \otimes \mathbf{D} + \mathbf{V}_E \otimes \mathbf{I}_N\right). \tag{7}$$

The covariance matrix of this model is highly sparse, but this sparsity is spread over many rows and columns. However, by pre-multiplying the left-hand side of our model by the appropriate commutation matrix, $\mathbf{K}^{(N,T)}$ (i.e., re-ordering observations such that we order by individuals first, and then by traits, rather than the other way around), we can obtain a model where the resulting covariance matrix $\mathbf{V}$ is block-diagonal. That is, based on Eq. 7, we can now write $\mathbf{y} = \mathbf{K}^{(N,T)}\text{vec}\left(\mathbf{P}^\top\mathbf{Y}^*\right) = \text{vec}\left(\mathbf{Y}^{*\top}\mathbf{P}\right) = \text{vec}\left(\mathbf{Y}\right)$ as follows:

$$\mathbf{y} \sim \mathcal{N}\left(\widetilde{\mathbf{X}}\boldsymbol{\beta}, \mathbf{V}\right), \text{ where } \mathbf{V} = \mathbf{D} \otimes \mathbf{V}_G + \mathbf{I}_N \otimes \mathbf{V}_E \text{ and } \widetilde{\mathbf{X}} = \begin{pmatrix} \mathbf{X}_1 \\ \mathbf{X}_2 \\ \vdots \\ \mathbf{X}_N \end{pmatrix}, \tag{8}$$

and where

$$\mathbf{X}_j = \begin{pmatrix} \mathbf{x}_{j1}^\top & & \mathbf{0} \\ & \ddots & \\ \mathbf{0} & & \mathbf{x}_{jT}^\top \end{pmatrix}, \tag{9}$$

is the $T \times K$ matrix of fixed-effect covariates across traits for observation $j = 1, \ldots, N$ after the linear transformation by the eigenvectors from the GRM. Here, K is the total number of covariates across traits (i.e., $K = \sum_{t=1}^{T} K_t$). More specifically, vector $\mathbf{x}_{jt}^\top$ equals the j-th row from the $N \times K_t$ matrices $\mathbf{P}^\top\mathbf{X}_t^*$, and

the vectors of zeros are conformable to the dimensions of $\mathbf{x}_{jt}$. In turn, $\mathbf{X}_t^*$ is the $N \times K_t$ matrix of fixed-effect covariates for trait t.

Notice that several asterisks have been dropped. That is, we here define our $T \times N$ matrix of outcomes $\mathbf{Y}$ as ${\mathbf{Y}^*}^\top \mathbf{P}$, and our grand matrix of fixed-effect covariates, $\widetilde{\mathbf{X}}$, and its constituents, $\mathbf{X}_j$ for $j = 1, \ldots, N$, and our covariance matrix, $\mathbf{V}$, as outlined above. These definitions boil down to pre-multiplication of conformable matrices by the transposed matrix of eigenvectors of the GRM, and re-ordering of rows of vectorised matrices in accordance with the commutation matrix. These definitions of $\mathbf{Y}$ and $\mathbf{X}_j$ for $j = 1, \ldots, N$ enable us to reduce the computational complexity of our model relatively easily. Notice that $\mathbf{V}$ can be written as follows:

$$\mathbf{V} = \begin{pmatrix} \mathbf{V}_1 & & \mathbf{0} \\ & \ddots & \\ \mathbf{0} & & \mathbf{V}_N \end{pmatrix}, \text{ where} \tag{10}$$

$$\mathbf{V}_j = d_j \mathbf{V}_G + \mathbf{V}_E \text{ for } j = 1, \ldots, N, \tag{11}$$

and where $\mathbf{V}_j$ is a $T \times T$ symmetric positive (semi)definite matrix. Thus, $\mathbf{V}$ is now a block-diagonal matrix in which each block on the diagonal is a $T \times T$ matrix. As such, $\mathbf{V}$ is nothing more than a linear combination of $\mathbf{V}_G$ and $\mathbf{V}_E$ with weights based on the consecutive eigenvalues of the GRM. This matrix has sufficient structure to permit significant computational gains.

The combination of (i) $d_j \geq 0 \,\forall\, j$, (ii) $\mathbf{V}_G$ being at least positive semidefinite, and (iii) $\mathbf{V}_E$ being positive definite, forms a set of sufficient conditions for $\mathbf{V}_j$ to be positive definite $\forall\, j$, and, in turn, for $\mathbf{V}$ to be positive definite. As the GRM is at least positive semidefinite by definition, the first condition holds for sure. Secondly, by choosing an appropriate parametrisation of our model, we ensure $\mathbf{V}_G$ is always at least positive semidefinite. Finally, by again choosing an appropriate parametrisation, we also ensure $\mathbf{V}_E$ is always at least positive semi-definite.

In further derivations, we make the stronger assumption that $\mathbf{V}_E$ is always positive definite (thus, by virtue of the set of sufficient conditions, ensuring $\mathbf{V}$ is always positive definite). Although this assumption may fail to hold in certain empirical applications (e.g., perfectly multicollinear traits as input for an MGREML analysis), it is safe to make this assumption for the derivations, especially since the software implementation of MGREML by default only accepts phenotypes that are not perfectly multicollinear. This requirement forces each phenotype to have its own idiosyncratic signal, even if that signal is very small.

The situation is in fact quite analogous to derivations for, e.g., the ordinary least squares (OLS) estimator, where one has the assumption that regressors (i.e., the explanatory variables) are not perfectly multicollinear although there is nothing preventing such collinearity to be present in empirical data. Analogous to OLS regression with perfect multicollinearity of regressors, MGREML produces an error in case multicollinearity among the phenotypes is too high.

Step 3. The log-likelihood function, its gradient, and the information matrix

Log-likelihood function. The generic log-likelihood of a mixed linear model for $n \times 1$ vector $\mathbf{y}$ (in our case $n = NT$), as a function of parameters in $\boldsymbol{\theta}$, with fixed-effect covariates $\widetilde{\mathbf{X}}$, and variance matrix $\mathbf{V}$, is given by

$$\log l(\boldsymbol{\theta}) = -\frac{1}{2}\left(n\log(2\pi) + \log|\mathbf{V}| + \log\left|\widetilde{\mathbf{X}}^{\top}\mathbf{V}^{-1}\widetilde{\mathbf{X}}\right| - \log\left|\widetilde{\mathbf{X}}^{\top}\widetilde{\mathbf{X}}\right| + \mathbf{y}^{\top}\mathbf{M}\mathbf{y}\right), \text{ where} \tag{12}$$

$$\mathbf{M} = \mathbf{V}^{-1} - \mathbf{V}^{-1}\widetilde{\mathbf{X}}\left(\widetilde{\mathbf{X}}^{\top}\mathbf{V}^{-1}\widetilde{\mathbf{X}}\right)^{-1}\widetilde{\mathbf{X}}^{\top}\mathbf{V}^{-1}. \tag{13}$$

Importantly, $\mathbf{V}$ is a function of $\boldsymbol{\theta}$. Thus, we aim to find the $\boldsymbol{\theta}$ that sets the matrix $\mathbf{V}$ such that it maximises the log-likelihood of the observed data under the assumed distribution. Although this model incorporates fixed-effects $\widetilde{\mathbf{X}}$ to correct for possible confounding effects, it is not concerned with estimating those fixed effects. The primary aim of the model is to estimate the parameters $\boldsymbol{\theta}$ that make the variance matrix fitting the data as well as possible. Nevertheless, one can still use, e.g., the generalised least squares (GLS) estimator to obtain estimates of the fixed effects, because the model yields estimates of $\widehat{\boldsymbol{\theta}}$ and $\widehat{\mathbf{V}}$. That is, one can obtain the fixed-effects estimates by:

$$\mathbf{b} = \left(\widetilde{\mathbf{X}}^{\top}\widehat{\mathbf{V}}^{-1}\widetilde{\mathbf{X}}\right)^{-1}\widetilde{\mathbf{X}}^{\top}\widehat{\mathbf{V}}^{-1}\mathbf{y}. \tag{14}$$

This option is also implemented in the `GCTA` software package (Yang et al., 2011). The generic log-likelihood in Eq. 12 is also described by Yang et al. (2011) and is, in turn, based on a broad literature, including the works of Harville (1977), Casella and Searle (1985), and Searle et al. (1992). This log-likelihood is such that the presence of fixed-effect covariates does not bias the estimation of variance components, something that typically occurs when applying classical maximum likelihood estimation to such problems. For the simple case without covariates, it is clear that in our case (without the canonical transformation and commutation) the matrix $\mathbf{V}$ is a full $(NT) \times (NT)$ matrix. Thus, under this naïve approach, even computing the log-likelihood requires calculation of the eigenvalues of $\mathbf{V}$ (in order to calculate $\log|\mathbf{V}|$ in a numerically stable

manner). These eigenvalues can be computed in $O\left(N^3T^3\right)$ time when using standard algorithms. As such, the complexity becomes prohibitively large when N and T increase. Alternative strategies to compute $\log|\mathbf{V}|$ (where $\mathbf{V}$ is a full $(NT) \times (NT)$ matrix) exist, yet involve a computational bottleneck requiring $O\left(N^3T^3\right)$ time.

Restricted maximum likelihood. In order to estimate $\mathbf{V}_G$ and $\mathbf{V}_E$, we need to find the parameters that maximise the log-likelihood function in Eq. 12. This approach is known as restricted maximum likelihood (REML). We are able to perform REML estimation efficiently by using highly efficient expressions for the log-likelihood and the gradient that we derive in subsequent sections. These expressions allow for rapid application of line-search methods and a BFGS algorithm. In addition, once our estimates have converged, we will use an efficient expression for the AI matrix (Gilmour et al., 1995). This expression will also be derived in subsequent sections. Given we can easily calculate the AI matrix, obtaining the covariance matrix of $\widehat{\boldsymbol{\theta}}$ is straightforward. In turn, we can use covariance matrix $\widehat{\boldsymbol{\theta}}$ in conjunction with a delta method to compute the standard errors of our estimates of genetic correlations and heritabilities. Derivations for this delta method are also provided later on in this section.

Gradient of the log-likelihood. For a given parameter θ_1 in the set of parameters $\boldsymbol{\theta}$ (using index 1 without loss of generality), the gradient of the log-likelihood, in accordance with Yang et al. (2011), is given by:

$$g_1 = \frac{1}{2}\mathbf{y}^\top \mathbf{M}\frac{\partial \mathbf{V}}{\partial \theta_1}\mathbf{M}\mathbf{y} - \frac{1}{2}\mathrm{tr}\left(\mathbf{M}\frac{\partial \mathbf{V}}{\partial \theta_1}\right). \tag{15}$$

As we will show later on, we are able to compute $NT \times 1$ vector $\mathbf{r} = \mathbf{M}\mathbf{y}$ in $O(NT)$ time in case covariates are absent in our model and in $O\left(NT^2\right)$ time in case there is a fairly limited number of covariates. Using the expression in Eq. 13 for $\mathbf{M}$, we can rewrite the gradient as follows:

$$g_1 = \frac{1}{2}\mathbf{r}^\top \frac{\partial \mathbf{V}}{\partial \theta_1}\mathbf{r} - \frac{1}{2}\mathrm{tr}\left(\mathbf{V}^{-1}\frac{\partial \mathbf{V}}{\partial \theta_1}\right) + \frac{1}{2}\mathrm{tr}\left(\mathbf{V}^{-1}\widetilde{\mathbf{X}}\left(\widetilde{\mathbf{X}}^\top \mathbf{V}^{-1}\widetilde{\mathbf{X}}\right)^{-1}\widetilde{\mathbf{X}}^\top \mathbf{V}^{-1}\frac{\partial \mathbf{V}}{\partial \theta_1}\right). \tag{16}$$

In our case, $\mathbf{V}$ is a block-diagonal matrix and therefore its inverse and its derivatives (both first- and second-order partial derivatives) also have a block-diagonal structure. These block-diagonal matrices have equally sized blocks. It holds for two block-diagonal matrices with equally-sized blocks, $\mathbf{B}$ and $\mathbf{C}$, that the block diagonal matrix resulting from their product has blocks that are given by the products of the corresponding

blocks in the two matrices. That is, block h in $\mathbf{BC}$ is the product of block h in $\mathbf{B}$ times block h in $\mathbf{C}$. Also, note that $\mathrm{tr}\,(AB) = \mathrm{tr}\,(BA)$ and that the trace of a block-diagonal matrix can be written as the sum of traces of those blocks. These insights can be used to further rewrite the gradient as follows:

$$g_1 = \frac{1}{2}\left[\left(\sum_{j=1}^{N} \mathbf{r}_j^\top \frac{\partial \mathbf{V}_j}{\partial \theta_1}\mathbf{r}_j\right) - \sum_{j=1}^{N} \mathrm{tr}\left(\mathbf{V}_j^{-1}\frac{\partial \mathbf{V}_j}{\partial \theta_1}\right) + \mathrm{tr}\left(\mathbf{Z}^{-1}\left(\sum_{j=1}^{N} \mathbf{X}_j^\top \mathbf{V}_j^{-1}\frac{\partial \mathbf{V}_j}{\partial \theta_1}\mathbf{V}_j^{-1}\mathbf{X}_j\right)\right)\right], \text{ where} \tag{17}$$

$$\mathbf{Z} = \widetilde{\mathbf{X}}^\top \mathbf{V}^{-1}\widetilde{\mathbf{X}} = \sum_{j=1}^{N} \mathbf{X}_j^\top \mathbf{V}_j^{-1}\mathbf{X}_j. \tag{18}$$

Although Equations 17 and 18 may seem more involved than Eq. 16, the gradient has now been decomposed into a contribution per observation. This decomposition is at the core of making time complexity for the gradient linear in the number of observations. For reasons of brevity, we refer to $\mathbf{Z}$ in Eq. 18 as the GLS precision matrix.

Information matrix of the log-likelihood. Without loss of generality, let $\mathcal{I}_{12}$ (resp. $\mathbb{E}\left[\mathcal{I}_{12}\right]$) denote the element from the observed (expected) information matrix, corresponding to parameters θ_1 and θ_2. Based on the work by Gilmour et al. (1995), we know that

$$\mathcal{I}_{12} = \frac{1}{2}\mathrm{tr}\left(\mathbf{M}\frac{\partial^2 \mathbf{V}}{\partial \theta_1 \partial \theta_2}\right) - \frac{1}{2}\mathrm{tr}\left(\mathbf{M}\frac{\partial \mathbf{V}}{\partial \theta_1}\mathbf{M}\frac{\partial \mathbf{V}}{\theta_2}\right) + \mathbf{y}^\top \mathbf{M}\frac{\partial \mathbf{V}}{\partial \theta_1}\mathbf{M}\frac{\partial \mathbf{V}}{\partial \theta_2}\mathbf{My} - \frac{1}{2}\mathbf{y}^\top \mathbf{M}\frac{\partial^2 \mathbf{V}}{\partial \theta_1 \partial \theta_2}\mathbf{My} \text{ and} \tag{19}$$

$$\mathbb{E}\left[\mathcal{I}_{12}\right] = \frac{1}{2}\mathrm{tr}\left(\mathbf{M}\frac{\partial \mathbf{V}}{\partial \theta_1}\mathbf{M}\frac{\partial \mathbf{V}}{\theta_2}\right). \tag{20}$$

The average of these two expressions can be written as follows:

$$\frac{1}{4}\mathrm{tr}\left(\mathbf{M}\frac{\partial^2 \mathbf{V}}{\partial \theta_1 \partial \theta_2}\right) - \frac{1}{4}\mathbf{y}^\top \mathbf{M}\frac{\partial^2 \mathbf{V}}{\partial \theta_1 \partial \theta_2}\mathbf{My} + \frac{1}{2}\mathbf{y}^\top \mathbf{M}\frac{\partial \mathbf{V}}{\partial \theta_1}\mathbf{M}\frac{\partial \mathbf{V}}{\partial \theta_2}\mathbf{My}. \tag{21}$$

As the computational complexity of this expression is high, Gilmour et al. (1995) note that $\mathbf{y}^\top \mathbf{M}\frac{\partial^2 \mathbf{V}}{\partial \theta_1 \partial \theta_2}\mathbf{My}$ can be approximated by its expectation $\mathrm{tr}\left(\mathbf{M}\frac{\partial^2 \mathbf{V}}{\partial \theta_1 \partial \theta_2}\right)$ when these second-order derivatives are non-zero, as is the case under our parametrisation.

In fact, when $\mathbf{V}$ is linear in the parameters of the model, these second-order derivatives with respect to $\mathbf{V}$ are zero at any rate, in which case the AI matrix is the exact average of the observed and expected information matrices. This exact average is used by others in the AI-REML literature (e.g., Yang et al. (2011)).

For our nonlinear parametrisation, under the approximation as proposed by Gilmour et al. (1995), the

expression for an element of the AI matrix is given by:

$$\overline{\mathcal{I}}_{12} = \frac{1}{2}\mathbf{y}^\top \mathbf{M}\frac{\partial \mathbf{V}}{\partial \theta_1}\mathbf{M}\frac{\partial \mathbf{V}}{\partial \theta_2}\mathbf{M}\mathbf{y}. \tag{22}$$

Thus, given the efficient expression of $\mathbf{My}$ and the block-diagonal structure of the problem, an element of the AI matrix can be described as:

$$\overline{\mathcal{I}}_{12} = \frac{1}{2}\left[\mathbf{r}^\top \frac{\partial \mathbf{V}}{\partial \theta_1}\mathbf{V}^{-1}\frac{\partial \mathbf{V}}{\partial \theta_2}\mathbf{r} - \mathbf{r}^\top \frac{\partial \mathbf{V}}{\partial \theta_1}\mathbf{V}^{-1}\widetilde{\mathbf{X}}\left(\widetilde{\mathbf{X}}^\top \mathbf{V}^{-1}\widetilde{\mathbf{X}}\right)^{-1}\widetilde{\mathbf{X}}^\top \mathbf{V}^{-1}\frac{\partial \mathbf{V}}{\partial \theta_2}\mathbf{r}\right] \tag{23}$$

$$= \frac{1}{2}\left[\left(\sum_{j=1}^{N}\mathbf{r}_j^\top \frac{\partial \mathbf{V}_j}{\partial \theta_1}\mathbf{V}_j^{-1}\frac{\partial \mathbf{V}_j}{\partial \theta_2}\mathbf{r}_j\right) - \left(\sum_{j=1}^{N}\mathbf{r}_j^\top \frac{\partial \mathbf{V}_j}{\partial \theta_1}\mathbf{V}_j^{-1}\mathbf{X}_j\right)\mathbf{Z}^{-1}\left(\sum_{j=1}^{N}\mathbf{X}_j^\top \mathbf{V}_j^{-1}\frac{\partial \mathbf{V}_j}{\partial \theta_2}\mathbf{r}\right)\right]. \tag{24}$$

Step 4. Tractable notation for different covariates across traits

In most derivations that will follow, we assume there is one set of k fixed-effect covariates that applies to all traits. In that case, we allow for Tk fixed effects in total. This assumption simplifies derivations considerably. However, here we show that the resulting expressions can easily be generalised to cases in which different sets of covariates apply to different traits, as well as to the case of having no covariates at all. The biggest advantage of considering one set of covariates that applies to all traits, is that we can write down the matrix of fixed-effect covariates for a particular observation j as a Kronecker product.

Let $\mathcal{K}_t$ denote the set of covariates that applies to trait t for $t = 1, \ldots, T$. We assume that $\mathcal{K} = \bigcup_{t=1}^{T}\mathcal{K}_t$ is the complete set of covariates, which, without loss of generality, for now applies to all traits. That is, $\mathcal{K} = \mathcal{K}_t \,\forall\, t$, with $k = ||\mathcal{K}||$ denoting the total number of unique covariates. Now, letting

$$\mathbf{X} = \mathbf{P}^\top \mathbf{X}^*, \tag{25}$$

with $\mathbf{X}^*$ and $\mathbf{X}$ denoting the $N \times k$ matrices of fixed-effect covariates (the latter after the canonical transformation) with observations in the rows and covariates in the columns, we obtain that the $T \times Tk$ matrices $\mathbf{X}_j$ for observations $j = 1, \ldots, N$, as defined in Eq. 9, can be written as:

$$\mathbf{X}_j = \mathbf{I}_T \otimes \mathbf{x}_j^\top. \tag{26}$$

Here, the $1 \times k$ vector $\mathbf{x}_j^\top$ is the j-th row from $\mathbf{X}$ for $j = 1, \ldots, N$. With this notation for the same set

of covariates applying to all traits, we can easily generalise to the case of different covariates for different traits. Now, assume there is a binary matrix $\mathbf{S}_t$ for each trait, such that $\mathbf{S}_t$ is a $K_t \times k$ matrix ($K_t \leq k$), with $\mathbf{S}_t\mathbf{S}_t^\top = \mathbf{I}_{K_t}$. When covariate j (from the full set of covariates, $\mathcal{K}$) is the i-th covariate for trait t, then element i, j in matrix $\mathbf{S}_t$ is equal to one. Otherwise, that element equals zero. Note that in case all covariates apply to a given trait, $\mathbf{S}_t$ is simply equal to $\mathbf{I}_k$. Effectively, $\mathbf{S}_t\mathbf{A}$ yields a submatrix of matrix $\mathbf{A}$, comprising only K_t unique rows from the k rows in $\mathbf{A}$. If matrix $\mathbf{A}$ is square, $\mathbf{S}_t\mathbf{A}\mathbf{S}_t^\top$ yields the $K_t \times K_t$ submatrix of $\mathbf{A}$ in which the appropriate rows and columns from $\mathbf{A}$ are selected.

Now, we can construct a grand block-diagonal matrix

$$\mathbf{S} = \begin{pmatrix} \mathbf{S}_1 & & \mathbf{0} \\ & \ddots & \\ \mathbf{0} & & \mathbf{S}_T \end{pmatrix}, \tag{27}$$

such that $\mathbf{S}\mathbf{S}^\top = \mathbf{I}_K$, where $K = \sum_{t=1}^{T} K_t$. Using these definitions, the GLS precision matrix can be written as:

$$\mathbf{Z} = \mathbf{S}\left[\sum_{j=1}^{N} \left(\mathbf{I}_T \otimes \mathbf{x}_j\right)\mathbf{V}_j^{-1}\left(\mathbf{I}_T \otimes \mathbf{x}_j^\top\right)\right]\mathbf{S}^\top, \tag{28}$$

and the $T \times K$ matrix of fixed-effect covariates for observation j as

$$\mathbf{X}_j = \left(\mathbf{I}_T \otimes \mathbf{x}_j^\top\right)\mathbf{S}^\top. \tag{29}$$

We now have a concise notation for the two possible scenarios. In the first scenario, there is a set of k fixed-effect covariates that applies to all traits. In the second scenario, there are different covariates for different traits. This notation help us to obtain efficient expressions for the log-likelihood, gradient, and the AI matrix.

Step 5. Calculating the log-likelihood efficiently

To calculate the log-likelihood rapidly, we need efficient expressions for $\mathbf{V}^{-1}$, $\log|\mathbf{V}|$, $\log|\mathbf{Z}|$, and $\mathbf{y}^\top\mathbf{M}\mathbf{y} = \mathbf{y}^\top\mathbf{r}$ with $\mathbf{r}$ referring to what we call the rescaled GLS residuals or, even more briefly, just residuals.

Decomposition of the variance matrix. The full variance matrix, $\mathbf{V}$, in our model in Eq. 8, has a block-diagonal structure as illustrated in Eq. 10 and Eq. 11. Hence, the inverse of $\mathbf{V}$ is a block-diagonal matrix too, with $\mathbf{V}_j^{-1}$ for $j = 1, \ldots, N$ as blocks. Also, the determinant of $\mathbf{V}$ is the product of the determinants of $\mathbf{V}_j$ for $j = 1, \ldots, N$. Let the EVD of $\mathbf{V}_E$ be given by $\mathbf{Q}\mathbf{\Phi}\mathbf{Q}^\top$, with $\mathbf{Q}\mathbf{Q}^\top = \mathbf{Q}^\top\mathbf{Q} = \mathbf{I}_T$. We then can rewrite $\mathbf{V}_j$ as follows:

$$\mathbf{V}_j = \mathbf{Q}\mathbf{\Phi}^{\frac{1}{2}}\left(d_j\widetilde{\mathbf{V}}_G + \mathbf{I}_T\right)\mathbf{\Phi}^{\frac{1}{2}}\mathbf{Q}^\top, \text{ where} \tag{30}$$

$$\widetilde{\mathbf{V}}_G = \mathbf{\Phi}^{-\frac{1}{2}}\mathbf{Q}^\top\mathbf{V}_G\mathbf{Q}\mathbf{\Phi}^{-\frac{1}{2}}. \tag{31}$$

As our model assumes a positive definite $\mathbf{V}_E$, $\mathbf{\Phi}$ is a diagonal matrix with positive diagonal entries and $\mathbf{\Phi}^{-\frac{1}{2}}$ is defined in terms of real numbers. Moreover, because $\mathbf{V}_G$ is at least positive semi-definite, so is $\widetilde{\mathbf{V}}_G$ (this can be shown easily using quadratic forms with respect to $\widetilde{\mathbf{V}}_G$, which can be rewritten as quadratic forms with respect to $\mathbf{V}_G$). Let $\mathbf{L}\mathbf{\Lambda}\mathbf{L}^\top$ denote the EVD of $\widetilde{\mathbf{V}}_G$, with $\mathbf{L}\mathbf{L}^\top = \mathbf{L}^\top\mathbf{L} = \mathbf{I}_T$. Now, we can rewrite $\mathbf{V}_j$ as:

$$\mathbf{V}_j = \mathbf{Q}\mathbf{\Phi}^{\frac{1}{2}}\mathbf{L}\left(d_j\mathbf{\Lambda} + \mathbf{I}_T\right)\mathbf{L}^\top\mathbf{\Phi}^{\frac{1}{2}}\mathbf{Q}^\top. \tag{32}$$

Hence, we have that

$$\mathbf{V}_j^{-1} = \mathbf{F}\mathbf{D}_j^*\mathbf{F}^\top, \text{ where} \tag{33}$$

$$\mathbf{D}_j^* = \left(d_j\mathbf{\Lambda} + \mathbf{I}_T\right)^{-1} \text{ and} \tag{34}$$

$$\mathbf{F} = \mathbf{Q}\mathbf{\Phi}^{-\frac{1}{2}}\mathbf{L}. \tag{35}$$

Note that although $\mathbf{F}$ is square, it is neither necessarily a symmetric nor necessarily an orthogonal matrix. By means of this expression for $\mathbf{V}_j$, we are able to invert the $(NT) \times (NT)$ covariance matrix $\mathbf{V}$ at the price of performing N matrix multiplications, each in $O\left(T^3\right)$ time. That is, multiplying $T \times T$ matrix $\mathbf{F}\mathbf{D}_j^*$ with $T \times T$ matrix $\mathbf{F}^\top$. Thus, we have reduced time for inverting $\mathbf{V}$ from $O\left(N^3T^3\right)$ to $O\left(NT^3\right)$.

In our case, a further reduction is possible, because we can exploit the fact that $\mathbf{V}_j^{-1}$ only varies from observation to observation in terms of the diagonal matrix $\mathbf{D}_j^*$. This insight allows us to derive fast expressions for the log-likelihood and gradient, because we do not need to calculate $\mathbf{V}_j^{-1}$ explicitly. As we will show, this implies that we can calculate the log-likelihood and gradient in $O\left(NT^2\right)$ time.

Efficient determinant of the variance matrix. For two conformable square matrices, $\mathbf{B}$ and $\mathbf{C}$, the following identity holds:

$$|\mathbf{AB}| = |\mathbf{A}|\,|\mathbf{B}|\,. \tag{36}$$

Moreover, the determinant of a block-diagonal matrix can be written as the product of the determinants of the blocks. Therefore, the determinant of $\mathbf{V}$ can be rewritten as:

$$\begin{aligned}
|\mathbf{V}| &= \prod_{j=1}^{N} |\mathbf{V}_j| && (37)\\
&= \prod_{j=1}^{N} \left(\left| \mathbf{Q}\mathbf{\Phi}^{\frac{1}{2}}\mathbf{L}\left(d_j\mathbf{\Lambda} + \mathbf{I}_T\right)\mathbf{L}^{\top}\mathbf{\Phi}^{\frac{1}{2}}\mathbf{Q}^{\top} \right| \right) && (38)\\
&= \prod_{j=1}^{N} \left(|\mathbf{Q}| \left|\mathbf{\Phi}^{\frac{1}{2}}\right| |\mathbf{L}| \left|d_j\mathbf{\Lambda} + \mathbf{I}_T\right| \left|\mathbf{L}^{\top}\right| \left|\mathbf{\Phi}^{\frac{1}{2}}\right| \left|\mathbf{Q}^{\top}\right| \right) && (39)\\
&= \prod_{j=1}^{N} \left(\left|d_j\mathbf{\Lambda} + \mathbf{I}_T\right| \left|\mathbf{L}^{\top}\right| |\mathbf{L}| \left|\mathbf{\Phi}^{\frac{1}{2}}\right| \left|\mathbf{\Phi}^{\frac{1}{2}}\right| \left|\mathbf{Q}^{\top}\right| |\mathbf{Q}| \right) && (40)\\
&= \prod_{j=1}^{N} \left(\left|d_j\mathbf{\Lambda} + \mathbf{I}_T\right| \left|\mathbf{L}^{\top}\mathbf{L}\right| |\mathbf{\Phi}| \left|\mathbf{Q}^{\top}\mathbf{Q}\right| \right) && (41)\\
&= \prod_{j=1}^{N} \left(\prod_{t=1}^{T} \left(d_j\lambda_t + 1\right) |\mathbf{I}_T| \prod_{t=1}^{T} \phi_t \,|\mathbf{I}_T| \right) && (42)\\
&= \prod_{j=1}^{N} \left(\prod_{t=1}^{T} \left(d_j\lambda_t + 1\right) \prod_{t=1}^{T} \phi_t \right), && (43)
\end{aligned}$$

where λ_t is the t-th diagonal entry of $\mathbf{\Lambda}$ and where ϕ_t is defined analogously with respect to $\mathbf{\Phi}$. Hence, the log-determinant of $\mathbf{V}$ is given by

$$\log|\mathbf{V}| = N \sum_{t=1}^{T} \log\left(\phi_t\right) + \sum_{j=1}^{N}\sum_{t=1}^{T} \log\left(d_j\lambda_t + 1\right), \tag{44}$$

where λ_t are the EVs of $\widetilde{\mathbf{V}}_G$ in $\mathbf{\Lambda}$, d_j are the EVs of the GRM, and ϕ_t the EVs of $\mathbf{V}_E$ in $\mathbf{\Phi}$. Calculating this log-determinant now takes $O\left(NT\right)$ time, given we have precomputed the EVD of the GRM, $\widetilde{\mathbf{V}}_G$, and $\mathbf{V}_E$.

GLS precision matrix, its inverse, and determinant. Here we start again with the assumption of identical covariates across traits (i.e., $\mathbf{S} = \mathbf{I}_{Tk}$). Using our expression for $\mathbf{V}_j^{-1}$ in Eq. 33 and properties of

the Kronecker product, the GLS precision matrix can be rewritten as:

$$\mathbf{Z} = \sum_{j=1}^{N} \mathbf{X}_j^\top \mathbf{V}_j^{-1} \mathbf{X}_j \tag{45}$$

$$= \sum_{j=1}^{N} (\mathbf{I}_T \otimes \mathbf{x}_j) \left(\mathbf{V}_j^{-1} \otimes \mathbf{I}_1\right) \left(\mathbf{I}_T \otimes \mathbf{x}_j^\top\right) \tag{46}$$

$$= \sum_{j=1}^{N} \left(\mathbf{V}_j^{-1} \otimes \mathbf{x}_j \mathbf{x}_j^\top\right) \tag{47}$$

$$= \sum_{j=1}^{N} \left(\left(\mathbf{F}\mathbf{D}_j^* \mathbf{F}^\top\right) \otimes \mathbf{x}_j \mathbf{x}_j^\top\right) \tag{48}$$

$$= \sum_{j=1}^{N} (\mathbf{F} \otimes \mathbf{I}_k) \left(\mathbf{D}_j^* \otimes \mathbf{x}_j \mathbf{x}_j^\top\right) \left(\mathbf{F}^\top \otimes \mathbf{I}_k\right) \tag{49}$$

$$= (\mathbf{F} \otimes \mathbf{I}_k) \left[\sum_{j=1}^{N} \left(\mathbf{D}_j^* \otimes \mathbf{x}_j \mathbf{x}_j^\top\right)\right] \left(\mathbf{F}^\top \otimes \mathbf{I}_k\right) \tag{50}$$

$$= (\mathbf{F} \otimes \mathbf{I}_k) \begin{bmatrix} \mathbf{B}_1 & & \mathbf{0} \\ & \ddots & \\ \mathbf{0} & & \mathbf{B}_T \end{bmatrix} \left(\mathbf{F}^\top \otimes \mathbf{I}_k\right), \text{ where } \mathbf{B}_t = \sum_{j=1}^{N} \frac{1}{d_j \lambda_t + 1} \mathbf{x}_j \mathbf{x}_j^\top = \mathbf{X}^\top \mathbf{D}_t^\dagger \mathbf{X} \text{ and } \mathbf{D}_t^\dagger = (\mathbf{D}\lambda_t + \mathbf{I}_N)^{-1}. \tag{51}$$

Recall here that $\mathbf{X}$ is the $N \times k$ matrix of covariates, after the canonical transformation. Given that $k = O(1)$, each matrix $\mathbf{B}_t$ can be computed in $O(N)$ time. We can, therefore, calculate $\mathbf{B}_t$ across all traits in $O(NT)$ time. The outer pre- and post-multiplications of the resulting block-diagonal matrix are trivial. Thus, overall, $\mathbf{Z}$ can be calculated in $O(NT)$ time. Notice here how we have avoided the need to calculate $\mathbf{V}_j^{-1}$ explicitly, thus avoiding a computation in $O\left(NT^3\right)$ time. We will pursue similar strategies for the remaining terms in the log-likelihood, gradient, and AI matrix.

The inverse of the precision matrix can be obtained efficiently, as:

$$\mathbf{Z}^{-1} = \left(\left(\mathbf{F}^{-1}\right)^\top \otimes \mathbf{I}_k\right) \mathbf{J} \left(\mathbf{F}^{-1} \otimes \mathbf{I}_k\right), \text{ with} \tag{52}$$

$$\mathbf{J} = \begin{pmatrix} \mathbf{B}_1^{-1} & & \mathbf{0} \\ & \ddots & \\ \mathbf{0} & & \mathbf{B}_T^{-1} \end{pmatrix}. \tag{53}$$

Here, $\mathbf{F}$ is the $T \times T$ matrix which is fixed across observations and parameters. Therefore, inverting $\mathbf{F}$

requires only $O(T^3)$ time. Moreover, the blocks $\mathbf{B}_t$ of the inner matrix can jointly be inverted in $O(T)$ time, assuming $k = O(1)$.

For the determinant of the precision matrix, we have that:

$$
\begin{aligned}
|\mathbf{Z}| &= |\mathbf{F} \otimes \mathbf{I}_k| \prod_{t=1}^{T} |\mathbf{B}_t| \left|\mathbf{F}^\top \otimes \mathbf{I}_k\right| && (54)\\
&= \prod_{t=1}^{T} |\mathbf{B}_t| \, |\mathbf{F} \otimes \mathbf{I}_k| \left|\mathbf{F}^\top \otimes \mathbf{I}_k\right| && (55)\\
&= \prod_{t=1}^{T} |\mathbf{B}_t| \left|(\mathbf{F} \otimes \mathbf{I}_k)\left(\mathbf{F}^\top \otimes \mathbf{I}_k\right)\right| && (56)\\
&= \prod_{t=1}^{T} |\mathbf{B}_t| \left|\mathbf{F}\mathbf{F}^\top \otimes \mathbf{I}_k\right| && (57)\\
&= \prod_{t=1}^{T} |\mathbf{B}_t| \left|\mathbf{F}\mathbf{F}^\top\right|^k && (58)\\
&= \prod_{t=1}^{T} |\mathbf{B}_t| \left|\mathbf{Q}\mathbf{\Phi}^{-\frac{1}{2}}\mathbf{L}\mathbf{L}^\top\mathbf{\Phi}^{-\frac{1}{2}}\mathbf{Q}^\top\right|^k && (59)\\
&= \prod_{t=1}^{T} |\mathbf{B}_t| \left|\mathbf{Q}\mathbf{\Phi}^{-1}\mathbf{Q}^\top\right|^k && (60)\\
&= \prod_{t=1}^{T} |\mathbf{B}_t| \left(|\mathbf{Q}| \left|\mathbf{\Phi}^{-1}\right| \left|\mathbf{Q}^\top\right|\right)^k && (61)\\
&= \prod_{t=1}^{T} |\mathbf{B}_t| \left(\left|\mathbf{\Phi}^{-1}\right| |\mathbf{Q}| \left|\mathbf{Q}^\top\right|\right)^k && (62)\\
&= \prod_{t=1}^{T} |\mathbf{B}_t| \left(\left|\mathbf{\Phi}^{-1}\right| \left|\mathbf{Q}\mathbf{Q}^\top\right|\right)^k && (63)\\
&= \prod_{t=1}^{T} |\mathbf{B}_t| \, |\mathbf{\Phi}|^{-k}. && (64)
\end{aligned}
$$

Here, $\mathbf{\Phi}$ are the EVs of $\mathbf{V}_E$. Therefore, the log-determinant of $\mathbf{Z}$ is:

$$
\log|\mathbf{Z}| = \sum_{t=1}^{T} \left(\log|\mathbf{B}_t| - k\log(\phi_t)\right), \tag{65}
$$

where $\mathbf{B}_t = \mathbf{X}^\top \mathbf{D}_t^\dagger \mathbf{X}$ and where ϕ_t denotes the t-th EV of $\mathbf{V}_E$. For numerical stability, we compute the EVD of $\mathbf{B}_t$ for $t = 1, \ldots, T$ and compute $\log|\mathbf{B}_t|$ by taking the sum of the logarithm of the resulting EVs. We use the same EVDs to set $\mathbf{B}_t^{-1}$.

In case we have different covariates across traits, our precision matrix is given by:

$$\mathbf{Z} = (\mathbf{S}(\mathbf{F} \otimes \mathbf{I}_k)) \begin{bmatrix} \mathbf{B}_1 & & \mathbf{0} \\ & \ddots & \\ \mathbf{0} & & \mathbf{B}_T \end{bmatrix} \left(\left(\mathbf{F}^\top \otimes \mathbf{I}_k\right) \mathbf{S}^\top\right). \tag{66}$$

Here, $\mathbf{S}$ is the binary $K \times Tk$ matrix as defined earlier. This equation implies $\mathbf{Z}$ can still be obtained in $O(NT)$ when the covariates differ by trait, assuming $k = O(1)$. In such a case, we compute the EVD of $\mathbf{Z}$ as it is (which can still be done in $O\left(T^3\right)$ time), and use this decomposition to compute both $\log|\mathbf{Z}|$ and $\mathbf{Z}^{-1}$.

GLS residuals. Expressions for the GLS residuals and $\mathbf{y}^\top\mathbf{M}\mathbf{y}$ can now also be rewritten such that we can compute them in $O\left(NT^2\right)$ time. To see this, note that for the case of indentical covariates across traits, we can compute the $T \times 1$ vectors of rescaled GLS residuals, $\mathbf{r}_j$, the $Tk \times 1$ vector of GLS estimates, $\mathbf{b}$, and the rescaled $T \times 1$ phenotype vectors, $\widetilde{\mathbf{y}}_j$, for $j = 1, \ldots, N$ as follows:

$$\mathbf{M}\mathbf{y} = \begin{pmatrix} \mathbf{r}_1 \\ \vdots \\ \mathbf{r}_N \end{pmatrix}, \text{ where} \tag{67}$$

$$\mathbf{r}_j = \widetilde{\mathbf{y}}_j - \mathbf{F}\left(\mathbf{D}_j^*\mathbf{F}^\top\left(\left(\mathbf{I}_T \otimes \mathbf{x}_j^\top\right)\mathbf{b}\right)\right), \tag{68}$$

$$\mathbf{b} = \mathbf{Z}^{-1}\left(\sum_{j=1}^{N}\left(\mathbf{I}_T \otimes \mathbf{x}_j\right)\widetilde{\mathbf{y}}_j\right), \text{ and} \tag{69}$$

$$\widetilde{\mathbf{y}}_j = \left(\mathbf{F}\left(\mathbf{D}_j^*\left(\mathbf{F}^\top\mathbf{y}_j\right)\right)\right). \tag{70}$$

Now, let $\widetilde{\mathbf{Y}} = (\widetilde{\mathbf{y}}_1 \quad \ldots \quad \widetilde{\mathbf{y}}_N)$ denote the $T \times N$ matrix of $T \times 1$ vectors $\widetilde{\mathbf{y}}_j$ for $j = 1, \ldots, N$. Then, $\mathbf{b}$ can be written as:

$$\mathbf{b} = \mathbf{Z}^{-1}\left(\sum_{j=1}^{N}\left(\mathbf{I}_T \otimes \mathbf{x}_j\right)\left(\widetilde{\mathbf{y}}_j \otimes \mathbf{I}_1\right)\right) \tag{71}$$

$$= \mathbf{Z}^{-1}\left(\sum_{j=1}^{N}\widetilde{\mathbf{y}}_j \otimes \mathbf{x}_j\right) \tag{72}$$

$$= \mathbf{Z}^{-1}\text{vec}\left(\mathbf{X}^\top\widetilde{\mathbf{Y}}^\top\right). \tag{73}$$

Given these expressions, $\widetilde{\mathbf{Y}}$ can be calculated in $O\left(NT^2\right)$ time. Moreover, given that $k = O(1)$, $\mathbf{X}^\top\widetilde{\mathbf{Y}}^\top$ can be calculated in $O(NT)$ time, provided we have $\widetilde{\mathbf{Y}}$. Using $\widetilde{\mathbf{Y}}$, $\mathbf{b}$, and $\left(\mathbf{I}_T \otimes \mathbf{x}_j^\top\right)$, it is straightforward to construct the rescaled GLS residuals. The full set of residuals can therefore be obtained in $O\left(NT^2\right)$ time.

Let $\mathbf{R} = \begin{pmatrix}\mathbf{r}_1 & \dots & \mathbf{r}_N\end{pmatrix}$ denote the $T \times N$ matrix of rescaled GLS residuals. Then, we obtain that:

$$\mathbf{y}^\top\mathbf{M}\mathbf{y} = \boldsymbol{\iota}^\top\left(\mathbf{R} \circ \mathbf{Y}\right)\boldsymbol{\iota}. \tag{74}$$

Here, $\boldsymbol{\iota}^\top$ is a $1 \times T$ vector of ones and $\boldsymbol{\iota}$ a $N \times 1$ vector of ones, and '$\circ$' denotes the Hadamard or element-wise product. Recall that $\mathbf{Y}$ is the $T \times N$ matrix of phenotypes after post-multiplication by $\mathbf{P}$, the eigenvectors of the GRM. In case we have different covariates across traits, we only need to modify our expressions for $\mathbf{b}$ and $\mathbf{r}_j$. In this particular case, we have that:

$$\mathbf{r}_j = \widetilde{\mathbf{y}}_j - \mathbf{F}\left(\mathbf{D}_j^*\mathbf{F}^\top\left(\left(\mathbf{I}_T \otimes \mathbf{x}_j^\top\right)\mathbf{S}^\top\mathbf{b}\right)\right), \text{ where} \tag{75}$$

$$\mathbf{b} = \mathbf{Z}^{-1}\mathbf{S}\text{vec}\left(\mathbf{X}^\top\widetilde{\mathbf{Y}}^\top\right). \tag{76}$$

Constant. In case we are interested in the constant term in the log-likelihood function, we need an efficient expression for $\log\left|\widetilde{\mathbf{X}}^\top\widetilde{\mathbf{X}}\right|$. Under identical covariates across traits, we have that:

$$\widetilde{\mathbf{X}}^\top\widetilde{\mathbf{X}} = \sum_{j=1}^{N}\left(\mathbf{I}_T \otimes \mathbf{x}_j\right)\left(\mathbf{I}_T \otimes \mathbf{x}_j^\top\right) \tag{77}$$

$$= \sum_{j=1}^{N}\left(\mathbf{I}_T \otimes \mathbf{x}_j\mathbf{x}_j^\top\right) \tag{78}$$

$$= \begin{pmatrix} \mathbf{X}^\top\mathbf{X} & & \mathbf{0} \\ & \ddots & \\ \mathbf{0} & & \mathbf{X}^\top\mathbf{X} \end{pmatrix}. \tag{79}$$

Here, $\mathbf{X}$ is the $N \times k$ matrix of covariates that is identical across all traits, after the canonical transformation. It therefore holds that:

$$\log\left|\widetilde{\mathbf{X}}^\top\widetilde{\mathbf{X}}\right| = T\log\left|\mathbf{X}^\top\mathbf{X}\right|, \tag{80}$$

where $\log\left|\mathbf{X}^{\top}\mathbf{X}\right|$ can be obtained in a numerically stable manner from the EVD of $\mathbf{X}^{\top}\mathbf{X}$. $\mathbf{X}^{\top}\mathbf{X}$ and its EVD can be obtained in $O(N)$ time, provided $k = O(1)$. Note that when there are different covariates across traits,

$$\log\left|\widetilde{\mathbf{X}}^{\top}\widetilde{\mathbf{X}}\right| = \sum_{t=1}^{T}\log\left|\mathbf{S}_t\mathbf{X}^{\top}\mathbf{X}\mathbf{S}_t^{\top}\right|. \tag{81}$$

This step involves taking submatrices of $\mathbf{X}^{\top}\mathbf{X}$ and computation of the determinants of those submatrices. When we assume $k = O(1)$, all these determinants can be obtained in $O(T)$ time in total. Here, the bottleneck is the calculation of $\mathbf{X}^{\top}\mathbf{X}$, which takes $O(N)$ time when $k = O(1)$. Thus, from a computational point of view, the calculation of $\log\left|\mathbf{X}^{\top}\mathbf{X}\right|$ is trivial for reasonable values of N and T, and for $k = O(1)$.

Overall complexity of the log-likelihood. We can compute the MGREML log-likelihood in $O\left(NT^2\right)$ time, provided the number of unique covariates is at most $O(1)$, irrespective of whether we have (i) no covariates at all, (ii) identical covariates across traits, or (iii) partial overlap between the sets of covariates that apply to the different traits. However, in spite of the same time complexity, we still expect the lowest runtime in the first case, as terms such as $\log|\mathbf{Z}|$ can then be ignored altogether. We expect the highest runtime in the third case, because of the additional steps that need to be taken in that case.

Step 6. Parametrisation of the covariance matrices

Before we can derive efficient expressions for the gradient and AI matrix, we need to settle the parametrisation of our model so that we can define the partial derivatives of $\mathbf{V}$ with respect to our parameters. To do so, we follow a simple factor model for both $\mathbf{V}_G$ and $\mathbf{V}_E$. That is:

$$\mathbf{V}_G = \mathbf{C}_G\mathbf{C}_G^{\top}, \text{ and} \tag{82}$$

$$\mathbf{V}_E = \mathbf{C}_E\mathbf{C}_E^{\top}, \tag{83}$$

where $\mathbf{C}_G$ has size $T \times F_G$ and $\mathbf{C}_E$ has size $T \times F_E$, with $F_G \leq T$ and (for identification purposes) $F_E = T$. Thus, we have at most as many genetic factors as we have traits, and we have as many environmental factors as we have traits. Importantly, these definitions ensure $\mathbf{V}_G$ and $\mathbf{V}_E$ are always valid covariance matrices (i.e., they are both at least positive semidefinite).

Partial derivatives. Let γ denote the genetic parameter in row t and column f of $\mathbf{C}_G$. This parameter can be conceptualised as the coefficient of the path from genetic factor f to trait t or, put differently, the effect of some latent genetic factor f (with unit variance) on trait t. By the product rule, the partial derivative of $\mathbf{V}_G$ with respect to this parameter is:

$$\frac{\partial \mathbf{V}_G}{\partial \gamma} = \frac{\partial \mathbf{C}_G}{\partial \gamma}\mathbf{C}_G^\top + \mathbf{C}_G \frac{\partial \mathbf{C}_G^\top}{\partial \gamma} \tag{84}$$

$$= \frac{\partial \mathbf{C}_G}{\partial \gamma}\mathbf{C}_G^\top + \left(\frac{\partial \mathbf{C}_G}{\partial \gamma}\mathbf{C}_G^\top\right)^\top . \tag{85}$$

Notice that the partial derivative of $\mathbf{C}_G$ with respect to γ is zero everywhere, except for the element in row t and column f. By recognising that we can write $\mathbf{C}_G$ as $(\mathbf{c}_{G1} \quad \dots \quad \mathbf{c}_{GF_G})$, where $\mathbf{c}_{Gf}$ is $T \times 1$ vector of coefficients from factor f to all traits, it follows that

$$\frac{\partial \mathbf{C}_G}{\partial \gamma}\mathbf{C}_G^\top = \frac{\partial \mathbf{C}_G}{\partial \gamma}\begin{pmatrix} \mathbf{c}_{G1}^\top \\ \vdots \\ \mathbf{c}_{GF_G}^\top \end{pmatrix} \tag{86}$$

$$= \begin{pmatrix} \mathbf{0}_{(t-1)\times T} \\ \mathbf{c}_{Gf}^\top \\ \mathbf{0}_{(T-t)\times T} \end{pmatrix} . \tag{87}$$

Therefore,

$$\frac{\partial \mathbf{V}_G}{\partial \gamma} = \begin{pmatrix} \mathbf{0}_{T\times(t-1)} & \mathbf{c}_{Gf} & \mathbf{0}_{T\times(T-t)} \end{pmatrix} + \begin{pmatrix} \mathbf{0}_{(t-1)\times T} \\ \mathbf{c}_{Gf}^\top \\ \mathbf{0}_{(T-t)\times T} \end{pmatrix} . \tag{88}$$

Analogously, with ε denoting the coefficient from environmental factor f to trait t, the partial derivative of $\mathbf{V}_E$ with respect to ε is:

$$\frac{\partial \mathbf{V}_E}{\partial \varepsilon} = \begin{pmatrix} \mathbf{0}_{T\times(t-1)} & \mathbf{c}_{Ef} & \mathbf{0}_{T\times(T-t)} \end{pmatrix} + \begin{pmatrix} \mathbf{0}_{(t-1)\times T} \\ \mathbf{c}_{Ef}^\top \\ \mathbf{0}_{(T-t)\times T} \end{pmatrix} . \tag{89}$$

Based on these expressions, it follows that:

$$\frac{\partial \mathbf{V}_j}{\partial \gamma} = d_j \left(\begin{pmatrix} \mathbf{0}_{T\times(t-1)} & \mathbf{c}_{Gf} & \mathbf{0}_{T\times(T-t)} \end{pmatrix} + \begin{pmatrix} \mathbf{0}_{(t-1)\times T} \\ \mathbf{c}_{Gf}^{\top} \\ \mathbf{0}_{(T-t)\times T} \end{pmatrix} \right) \text{ and} \tag{90}$$

$$\frac{\partial \mathbf{V}_j}{\partial \varepsilon} = \left(\begin{pmatrix} \mathbf{0}_{T\times(t-1)} & \mathbf{c}_{Ef} & \mathbf{0}_{T\times(T-t)} \end{pmatrix} + \begin{pmatrix} \mathbf{0}_{(t-1)\times T} \\ \mathbf{c}_{Ef}^{\top} \\ \mathbf{0}_{(T-t)\times T} \end{pmatrix} \right). \tag{91}$$

Hence, the partial derivatives of the covariance matrices for observations $j = 1, \ldots, N$, with respect to environmental parameters, do not have any observation-specific terms. Moreover, for genetic parameters, except for a scaling coefficient d_j (i.e., the EVs from the GRM), the partial derivatives of the covariance matrices for observations $j = 1, \ldots, N$ are also independent of observation-specific terms. This insight is useful in reducing the computational complexity of the gradient and AI matrix.

Step 7. Calculating the gradient efficiently

From the expression of the gradient of the log-likelihood in Eq. 17, it follows that we need efficient expressions for the squared sum of residuals and two traces.

Squared sum of residuals. Here, we seek an efficient expression for $\sum_{j=1}^{N} \mathbf{r}_j^{\top} \frac{\partial \mathbf{V}_j}{\partial \theta_1} \mathbf{r}_j$. In Step 5, we already derived efficient expressions for computing $\mathbf{r}_j$ for $j = 1, \ldots, N$ in $O\left(NT^2\right)$ time. For θ_1 being a genetic parameter γ, denoting the coefficient from genetic factor f to trait t, we therefore obtain the following

expression:

$$\sum_{j=1}^{N} \mathbf{r}_j^\top \frac{\partial \mathbf{V}_j}{\partial \theta_1} \mathbf{r}_j = \sum_{j=1}^{N} d_j \mathbf{r}_j^\top \left[\begin{pmatrix} \mathbf{0}_{T\times(t-1)} & \mathbf{c}_{Gf} & \mathbf{0}_{T\times(T-t)} \end{pmatrix} + \begin{pmatrix} \mathbf{0}_{(t-1)\times T} \\ \mathbf{c}_{Gf}^\top \\ \mathbf{0}_{(T-t)\times T} \end{pmatrix} \right] \mathbf{r}_j \tag{92}$$

$$= \sum_{j=1}^{N} d_j \left[\begin{pmatrix} \mathbf{0}_{1\times(t-1)} & \mathbf{r}_j^\top \mathbf{c}_{Gf} & \mathbf{0}_{1\times(T-t)} \end{pmatrix} \mathbf{r}_j + \mathbf{r}_j^\top \begin{pmatrix} \mathbf{0}_{(t-1)\times 1} \\ \mathbf{c}_{Gf}^\top \mathbf{r}_j \\ \mathbf{0}_{(T-t)\times 1} \end{pmatrix} \right] \tag{93}$$

$$= \sum_{j=1}^{N} d_j \left[\mathbf{r}_j^\top \mathbf{c}_{Gf} R_{tj} + R_{jt} \mathbf{c}_{Gf}^\top \mathbf{r}_j \right] \tag{94}$$

$$= 2 \sum_{j=1}^{N} R_{tj} d_j \left(\mathbf{r}_j^\top \mathbf{c}_{Gf} \right). \tag{95}$$

Here, R_{tj} denotes element t, j from the $T \times N$ matrix of rescaled GLS residuals, $\mathbf{R}$. Thus, R_{tj} is the rescaled GLS residual for individual j when explaining trait t. We can rewrite this expression much more compactly and efficiently as:

$$\sum_{j=1}^{N} \mathbf{r}_j^\top \frac{\partial \mathbf{V}_j}{\partial \theta_1} \mathbf{r}_j = \left\{ 2\mathbf{R}\mathbf{D}\mathbf{R}^\top \mathbf{C}_G \right\}_{tf}, \tag{96}$$

where $\{\mathbf{A}\}_{ij}$ denotes the element in row i and column j of a given matrix $\mathbf{A}$, and where $\mathbf{D}$ denotes the diagonal matrix with EVs from the GRM. As $\mathbf{D}$ is a diagonal matrix, calculating $\mathbf{R}\mathbf{D}\mathbf{R}^\top \mathbf{C}_G$ is trivial (e.g., construct $\widetilde{\mathbf{R}} = \mathbf{R}\mathbf{D}^{\frac{1}{2}}$ and take $\widetilde{\mathbf{R}}^\top \widetilde{\mathbf{R}} \mathbf{C}_G$). Importantly, notice that the matrix product $2\mathbf{R}\mathbf{D}\mathbf{R}^\top \mathbf{C}_G$ provides the values of $\sum_{j=1}^{N} \mathbf{r}_j^\top \frac{\partial \mathbf{V}_j}{\partial \theta_1} \mathbf{r}_j$ for all genetic parameters. Therefore, given some genetic parameter γ, we just need to take the appropriate row and column from this matrix to obtain the contribution of $\sum_{j=1}^{N} \mathbf{r}_j^\top \frac{\partial \mathbf{V}_j}{\partial \theta_1} \mathbf{r}_j$ to the element of the gradient vector that corresponds to that parameter. Analogously, for θ_1 being a environmental parameter ε denoting the coefficient from environmental factor f to trait t, we get:

$$\sum_{j=1}^{N} \mathbf{r}_j^\top \frac{\partial \mathbf{V}_j}{\partial \varepsilon} \mathbf{r}_j = \left\{ 2\mathbf{R}\mathbf{R}^\top \mathbf{C}_E \right\}_{tf}. \tag{97}$$

Notice that by computing $2\mathbf{R}\mathbf{D}\mathbf{R}^\top \mathbf{C}_G$ and $2\mathbf{R}\mathbf{R}^\top \mathbf{C}_E$, we obtain two matrices of which the appropriate entries are the values of $\sum_{j=1}^{N} \mathbf{r}_j^\top \frac{\partial \mathbf{V}_j}{\partial \theta_1} \mathbf{r}_j$ for all parameters in our model. Thus, calculating this part of the gradient has a computational complexity equal to that of computing these two matrices. This can be done in

$O\left(NT^2\right)$ time, because the number of factors in our model is at most proportional to the number of traits.

First trace. Next, we need an efficient expression for $\sum_{j=1}^{N} \operatorname{tr}\left(\mathbf{V}_j^{-1}\frac{\partial \mathbf{V}_j}{\partial \theta_1}\right)$. First, let us define

$$\mathbf{T}_G = \sum_{j=1}^{N} \mathbf{V}_j^{-1} d_j \text{ and } \mathbf{T}_E = \sum_{j=1}^{N} \mathbf{V}_j^{-1}. \tag{98}$$

By substituting $\mathbf{V}_j^{-1}$ with the expression for it in Eq. 33, we get:

$$\mathbf{T}_G = \mathbf{F}\left(\sum_{j=1}^{N} d_j \mathbf{D}_j^*\right)\mathbf{F}^\top \text{ and } \mathbf{T}_E = \mathbf{F}\left(\sum_{j=1}^{N} \mathbf{D}_j^*\right)\mathbf{F}^\top. \tag{99}$$

These expressions imply that both $\mathbf{T}_G$ and $\mathbf{T}_E$ can be obtained by taking a sum of diagonal matrices, and by respectively pre- and post-multiplying the resultant matrices by $\mathbf{F}$ and $\mathbf{F}^\top$.

Now, let us consider a genetic parameter, γ, denoting the effect of genetic factor f on trait t. In this case, we have that:

$$\sum_{j=1}^{N} \operatorname{tr}\left(\mathbf{V}_j^{-1}\frac{\partial \mathbf{V}_j}{\partial \gamma}\right) = \operatorname{tr}\left(\sum_{j=1}^{N}\left(\mathbf{V}_j^{-1} d_j \frac{\partial \mathbf{V}_G}{\partial \gamma}\right)\right) = \operatorname{tr}\left(\left(\sum_{j=1}^{N}\mathbf{V}_j^{-1} d_j\right)\frac{\partial \mathbf{V}_G}{\partial \gamma}\right) = \operatorname{tr}\left(\mathbf{T}_G \frac{\partial \mathbf{V}_G}{\partial \gamma}\right) \tag{100}$$

$$= \operatorname{tr}\left(\mathbf{T}_G\left(\begin{pmatrix} \mathbf{0}_{T\times(t-1)} & \mathbf{c}_{Gf} & \mathbf{0}_{T\times(T-t)} \end{pmatrix} + \begin{pmatrix} \mathbf{0}_{(t-1)\times T} \\ \mathbf{c}_{Gf}^\top \\ \mathbf{0}_{(T-t)\times T} \end{pmatrix}\right)\right) \tag{101}$$

$$= \operatorname{tr}\left(\begin{pmatrix} \mathbf{0}_{T\times(t-1)} & \mathbf{T}_G\mathbf{c}_{Gf} & \mathbf{0}_{T\times(T-t)} \end{pmatrix}\right) + \operatorname{tr}\left(\mathbf{T}_G\begin{pmatrix} \mathbf{0}_{(t-1)\times T} \\ \mathbf{c}_{Gf}^\top \\ \mathbf{0}_{(T-t)\times T} \end{pmatrix}\right) \tag{102}$$

$$= 2\operatorname{tr}\left(\begin{pmatrix} \mathbf{0}_{T\times(t-1)} & \mathbf{T}_G\mathbf{c}_{Gf} & \mathbf{0}_{T\times(T-t)} \end{pmatrix}\right) \tag{103}$$

$$= \left\{2\mathbf{T}_G\mathbf{C}_G\right\}_{tf}. \tag{104}$$

Analogously, for environmental parameter ε, indicating the effect of environmental factor f on trait t, we have:

$$\sum_{j=1}^{N} \operatorname{tr}\left(\mathbf{V}_j^{-1}\frac{\partial \mathbf{V}_j}{\partial \varepsilon}\right) = \left\{2\mathbf{T}_E\mathbf{C}_E\right\}_{tf}. \tag{105}$$

Matrices $\mathbf{T}_E$ and $\mathbf{T}_G$ can be obtained in $O(NT)$ time. Further calculation of $2\mathbf{T}_G\mathbf{C}_G$ and $2\mathbf{T}\mathbf{C}_E$ is trivial. By calculating these two matrices and taking the appropriate elements, we have the contribution of each parameter in our model to the gradient with respect to the $\sum_{j=1}^{N} \text{tr}\left(\mathbf{V}_j^{-1}\frac{\partial \mathbf{V}_j}{\partial \theta_1}\right)$ term. Thus, calculating this term for all parameters in the model can be done in $O(NT)$ time.

Second trace. To derive an efficient expression for the second trace, we start with the case of different covariates for each traits and then consider the special case of equal covariates across traits. To see how the computational complexity of the second trace can be reduced, even when we have different covariates per trait, first note that our aim is to find an efficient expression for the following term from Eq. 17:

$$\text{tr}\left(\mathbf{Z}^{-1}\left(\sum_{j=1}^{N}\mathbf{X}_j^\top\mathbf{V}_j^{-1}\frac{\partial \mathbf{V}_j}{\partial \theta_1}\mathbf{V}_j^{-1}\mathbf{X}_j\right)\right). \tag{106}$$

Here, we cannot use properties of the Kronecker product to make certain terms cancel out with respect to the inverse of $\mathbf{Z}$. Therefore, our derivations focus here on finding an efficient expression for $\mathbf{X}_j^\top\mathbf{V}_j^{-1}\frac{\partial \mathbf{V}_j}{\partial \theta_1}\mathbf{V}_j^{-1}\mathbf{X}_j$. However, it is important to recognise that it is still possible to write our matrix of covariates as a Kronecker product, post-multiplied by $\mathbf{S}^\top$. That is, we have that:

$$\mathbf{X}_j = \left(\mathbf{I}_T \otimes \mathbf{x}_j^\top\right)\mathbf{S}^\top. \tag{107}$$

Let's first focus on the term for γ. Because of the constancy of the partial derivatives for $\mathbf{V}_j$ with respect to γ over the observations (except for scalar d_j), we have that:

$$\begin{aligned}
\sum_{j=1}^{N}\mathbf{X}_j^\top\mathbf{V}_j^{-1}\frac{\partial \mathbf{V}_j}{\partial \gamma}\mathbf{V}_j^{-1}\mathbf{X}_j &= \sum_{j=1}^{N} d_j\mathbf{X}_j^\top\mathbf{V}_j^{-1}\frac{\partial \mathbf{V}_G}{\partial \gamma}\mathbf{V}_j^{-1}\mathbf{X}_j & (108)\\
&= \sum_{j=1}^{N} d_j\mathbf{S}\left(\mathbf{I}_T \otimes \mathbf{x}_j\right)\mathbf{F}\mathbf{D}_j^*\mathbf{F}^\top\frac{\partial \mathbf{V}_G}{\partial \gamma}\mathbf{F}\mathbf{D}_j^*\mathbf{F}^\top\left(\mathbf{I}_T \otimes \mathbf{x}_j^\top\right)\mathbf{S}^\top & (109)\\
&= \mathbf{S}\left[\sum_{j=1}^{N} d_j\left(\mathbf{I}_T \otimes \mathbf{x}_j\right)\mathbf{F}\mathbf{D}_j^*\mathbf{F}^\top\frac{\partial \mathbf{V}_G}{\partial \gamma}\mathbf{F}\mathbf{D}_j^*\mathbf{F}^\top\left(\mathbf{I}_T \otimes \mathbf{x}_j^\top\right)\right]\mathbf{S}^\top. & (110)
\end{aligned}$$

Thus, using $\mathbf{S}$ to select the appropriate submatrix of the $Tk\times Tk$ matrix between square brackets is something that can be done after aggregating observations. Therefore, we can focus on finding an efficient expression

for the term in between the square brackets. For that term, we have that:

$$\sum_{j=1}^{N} d_j \left(\mathbf{I}_T \otimes \mathbf{x}_j\right) \mathbf{F}\mathbf{D}_j^* \mathbf{F}^\top \frac{\partial \mathbf{V}_G}{\partial \gamma} \mathbf{F}\mathbf{D}_j^* \mathbf{F}^\top \left(\mathbf{I}_T \otimes \mathbf{x}_j^\top\right) \tag{111}$$

$$= \sum_{j=1}^{N} d_j \left(\mathbf{I}_T \otimes \mathbf{x}_j\right) \left(\mathbf{F} \otimes \mathbf{I}_1\right) \mathbf{D}_j^* \mathbf{F}^\top \frac{\partial \mathbf{V}_G}{\partial \gamma} \mathbf{F}\mathbf{D}_j^* \left(\mathbf{F}^\top \otimes \mathbf{I}_1\right) \left(\mathbf{I}_T \otimes \mathbf{x}_j^\top\right) \tag{112}$$

$$= \sum_{j=1}^{N} d_j \left(\mathbf{F} \otimes \mathbf{x}_j\right) \mathbf{D}_j^* \mathbf{F}^\top \frac{\partial \mathbf{V}_G}{\partial \gamma} \mathbf{F}\mathbf{D}_j^* \left(\mathbf{F}^\top \otimes \mathbf{x}_j^\top\right) \tag{113}$$

$$= \sum_{j=1}^{N} d_j \left(\mathbf{F} \otimes \mathbf{I}_k\right) \left(\mathbf{I}_T \otimes \mathbf{x}_j\right) \mathbf{D}_j^* \mathbf{F}^\top \frac{\partial \mathbf{V}_G}{\partial \gamma} \mathbf{F}\mathbf{D}_j^* \left(\mathbf{I}_T \otimes \mathbf{x}_j^\top\right) \left(\mathbf{F}^\top \otimes \mathbf{I}_k\right) \tag{114}$$

$$= \left(\mathbf{F} \otimes \mathbf{I}_k\right) \left[\sum_{j=1}^{N} d_j \left(\mathbf{I}_T \otimes \mathbf{x}_j\right) \mathbf{D}_j^* \mathbf{F}^\top \frac{\partial \mathbf{V}_G}{\partial \gamma} \mathbf{F}\mathbf{D}_j^* \left(\mathbf{I}_T \otimes \mathbf{x}_j^\top\right)\right] \left(\mathbf{F}^\top \otimes \mathbf{I}_k\right) \tag{115}$$

$$= \left(\mathbf{F} \otimes \mathbf{I}_k\right) \left[\sum_{j=1}^{N} \left(\mathbf{I}_T \otimes \mathbf{x}_j\right) \left(\left(d_j \mathbf{D}_j^* \mathbf{F}^\top \frac{\partial \mathbf{V}_G}{\partial \gamma} \mathbf{F}\mathbf{D}_j^*\right) \otimes \mathbf{I}_1\right) \left(\mathbf{I}_T \otimes \mathbf{x}_j^\top\right)\right] \left(\mathbf{F}^\top \otimes \mathbf{I}_k\right) \tag{116}$$

$$= \left(\mathbf{F} \otimes \mathbf{I}_k\right) \left[\sum_{j=1}^{N} \left(\left(d_j \mathbf{D}_j^* \mathbf{F}^\top \frac{\partial \mathbf{V}_G}{\partial \gamma} \mathbf{F}\mathbf{D}_j^*\right) \otimes \left(\mathbf{x}_j \mathbf{x}_j^\top\right)\right)\right] \left(\mathbf{F}^\top \otimes \mathbf{I}_k\right). \tag{117}$$

The Kronecker product in the summand can be written as:

$$\left(d_j \mathbf{D}_j^* \mathbf{F}^\top \frac{\partial \mathbf{V}_G}{\partial \gamma} \mathbf{F}\mathbf{D}_j^*\right) \otimes \left(\mathbf{x}_j \mathbf{x}_j^\top\right) \tag{118}$$

$$= \left(\left(\mathbf{D}_j^* \mathbf{F}^\top\right) \otimes \mathbf{I}_k\right) \left(\left(d_j \frac{\partial \mathbf{V}_G}{\partial \gamma}\right) \otimes \left(\mathbf{x}_j \mathbf{x}_j^\top\right)\right) \left(\left(\mathbf{F}\mathbf{D}_j^*\right) \otimes \mathbf{I}_k\right). \tag{119}$$

The middle Kronecker product in the preceding expression can be written as:

$$d_j \left[\begin{pmatrix} \mathbf{0}_{T\times(t-1)} & \mathbf{c}_{Gf} & \mathbf{0}_{T\times(T-t)} \end{pmatrix} \otimes \left(\mathbf{x}_j \mathbf{x}_j^\top\right) + \begin{pmatrix} \mathbf{0}_{(t-1)\times T} \\ \mathbf{c}_{Gf}^\top \\ \mathbf{0}_{(T-t)\times T} \end{pmatrix} \otimes \left(\mathbf{x}_j \mathbf{x}_j^\top\right)\right] \tag{120}$$

$$= d_j \begin{pmatrix} \mathbf{0}_{k\times(t-1)k} & c_{Gf1}\mathbf{x}_j\mathbf{x}_j^\top & \mathbf{0}_{k\times(T-t)k} \\ \vdots & \vdots & \vdots \\ \mathbf{0}_{k\times(t-1)k} & c_{GfT}\mathbf{x}_j\mathbf{x}_j^\top & \mathbf{0}_{k\times(T-t)k} \end{pmatrix} + d_j \begin{pmatrix} \mathbf{0}_{k\times(t-1)k} & c_{Gf1}\mathbf{x}_j\mathbf{x}_j^\top & \mathbf{0}_{k\times(T-t)k} \\ \vdots & \vdots & \vdots \\ \mathbf{0}_{k\times(t-1)k} & c_{GfT}\mathbf{x}_j\mathbf{x}_j^\top & \mathbf{0}_{k\times(T-t)k} \end{pmatrix}^\top, \tag{121}$$

with c_{Gft} denoting the genetic effect of genetic factor f on trait t. Thus, the Kronecker product in the summand can be written as:

$$\left(d_j \mathbf{D}_j^* \mathbf{F}^\top \frac{\partial \mathbf{V}_G}{\partial \gamma} \mathbf{F} \mathbf{D}_j^*\right) \otimes \left(\mathbf{x}_j \mathbf{x}_j^\top\right) \tag{122}$$

$$= d_j \mathbf{H}_j + d_j \mathbf{H}_j^\top, \tag{123}$$

where

$$\mathbf{H}_j = \left(\left(\mathbf{D}_j^* \mathbf{F}^\top\right) \otimes \mathbf{I}_k\right) \begin{pmatrix} \mathbf{0}_{k\times(t-1)k} & c_{Gf1}\mathbf{x}_j\mathbf{x}_j^\top & \mathbf{0}_{k\times(T-t)k} \\ \vdots & \vdots & \vdots \\ \mathbf{0}_{k\times(t-1)k} & c_{GfT}\mathbf{x}_j\mathbf{x}_j^\top & \mathbf{0}_{k\times(T-t)k} \end{pmatrix} \left(\left(\mathbf{F}\mathbf{D}_j^*\right) \otimes \mathbf{I}_k\right) \tag{124}$$

$$= \begin{pmatrix} \mathbf{0}_{k\times(t-1)k} & \mathbf{x}_j\mathbf{x}_j^\top \left(d_j\lambda_1+1\right)^{-1} \sum_{s=1}^T c_{Gfs}F_{s1} & \mathbf{0}_{k\times(T-t)k} \\ \vdots & \vdots & \vdots \\ \mathbf{0}_{k\times(t-1)k} & \mathbf{x}_j\mathbf{x}_j^\top \left(d_j\lambda_T+1\right)^{-1} \sum_{s=1}^T c_{Gfs}F_{sT} & \mathbf{0}_{k\times(T-t)k} \end{pmatrix} \left(\left(\mathbf{F}\mathbf{D}_j^*\right) \otimes \mathbf{I}_k\right) \tag{125}$$

$$= \begin{pmatrix} \mathbf{0}_{k\times(t-1)k} & \mathbf{x}_j\mathbf{x}_j^\top \left(d_j\lambda_1+1\right)^{-1} \left\{\mathbf{c}_{Gf}^\top \mathbf{F}\right\}_1 & \mathbf{0}_{k\times(T-t)k} \\ \vdots & \vdots & \vdots \\ \mathbf{0}_{k\times(t-1)k} & \mathbf{x}_j\mathbf{x}_j^\top \left(d_j\lambda_T+1\right)^{-1} \left\{\mathbf{c}_{Gf}^\top \mathbf{F}\right\}_T & \mathbf{0}_{k\times(T-t)k} \end{pmatrix} \left(\left(\mathbf{F}\mathbf{D}_j^*\right) \otimes \mathbf{I}_k\right) \tag{126}$$

$$= \begin{pmatrix} \mathbf{x}_j\mathbf{x}_j^\top \left(d_j\lambda_1+1\right)^{-1}\left(d_j\lambda_1+1\right)^{-1} \left\{\mathbf{c}_{Gf}^\top \mathbf{F}\right\}_1 F_{t1} & \dots & \mathbf{x}_j\mathbf{x}_j^\top \left(d_j\lambda_1+1\right)^{-1}\left(d_j\lambda_T+1\right)^{-1} \left\{\mathbf{c}_{Gf}^\top \mathbf{F}\right\}_1 F_{tT} \\ \vdots & & \vdots \\ \mathbf{x}_j\mathbf{x}_j^\top \left(d_j\lambda_T+1\right)^{-1}\left(d_j\lambda_1+1\right)^{-1} \left\{\mathbf{c}_{Gf}^\top \mathbf{F}\right\}_T F_{t1} & \dots & \mathbf{x}_j\mathbf{x}_j^\top \left(d_j\lambda_T+1\right)^{-1}\left(d_j\lambda_T+1\right)^{-1} \left\{\mathbf{c}_{Gf}^\top \mathbf{F}\right\}_T F_{tT} \end{pmatrix} \tag{127}$$

$$= \begin{pmatrix} \mathbf{x}_j\mathbf{x}_j^\top \left(d_j\lambda_1+1\right)^{-1}\left(d_j\lambda_1+1\right)^{-1} \left\{\mathbf{C}_{G}^\top \mathbf{F}\right\}_{f1} F_{t1} & \dots & \mathbf{x}_j\mathbf{x}_j^\top \left(d_j\lambda_1+1\right)^{-1}\left(d_j\lambda_T+1\right)^{-1} \left\{\mathbf{C}_{G}^\top \mathbf{F}\right\}_{f1} F_{tT} \\ \vdots & & \vdots \\ \mathbf{x}_j\mathbf{x}_j^\top \left(d_j\lambda_T+1\right)^{-1}\left(d_j\lambda_1+1\right)^{-1} \left\{\mathbf{C}_{G}^\top \mathbf{F}\right\}_{fT} F_{t1} & \dots & \mathbf{x}_j\mathbf{x}_j^\top \left(d_j\lambda_T+1\right)^{-1}\left(d_j\lambda_T+1\right)^{-1} \left\{\mathbf{C}_{G}^\top \mathbf{F}\right\}_{fT} F_{tT} \end{pmatrix}. \tag{128}$$

In this expression, $\{\mathbf{v}\}_i$ denotes element i in a given vector $\mathbf{v}$ and F_{ij} denotes element i, j in $\mathbf{F}$. As a result,

$$\sum_{j=1}^{N} d_j \left(\mathbf{I}_T \otimes \mathbf{x}_j\right) \mathbf{F}\mathbf{D}_j^{*}\mathbf{F}^{\top} \frac{\partial \mathbf{V}_G}{\partial \gamma} \mathbf{F}\mathbf{D}_j^{*}\mathbf{F}^{\top} \left(\mathbf{I}_T \otimes \mathbf{x}_j^{\top}\right) \tag{129}$$

$$= \left(\mathbf{F} \otimes \mathbf{I}_k\right) \left[\sum_{j=1}^{N} d_j \mathbf{H}_j + d_j \mathbf{H}_j^{\top}\right] \left(\mathbf{F}^{\top} \otimes \mathbf{I}_k\right) \tag{130}$$

$$= \left[\left(\mathbf{F} \otimes \mathbf{I}_k\right) \left(\sum_{j=1}^{N} d_j \mathbf{H}_j\right) \left(\mathbf{F}^{\top} \otimes \mathbf{I}_k\right)\right] + \left[\left(\mathbf{F} \otimes \mathbf{I}_k\right) \left(\sum_{j=1}^{N} d_j \mathbf{H}_j\right)^{\top} \left(\mathbf{F}^{\top} \otimes \mathbf{I}_k\right)\right]. \tag{131}$$

Therefore,

$$\operatorname{tr}\left(\mathbf{Z}^{-1}\left(\sum_{j=1}^{N} \mathbf{X}_j^{\top} \mathbf{V}_j^{-1} \frac{\partial \mathbf{V}_j}{\partial \gamma} \mathbf{V}_j^{-1} \mathbf{X}_j\right)\right) \tag{132}$$

$$= \operatorname{tr}\left(\mathbf{Z}^{-1}\mathbf{S}\left(\left[\left(\mathbf{F} \otimes \mathbf{I}_k\right) \left(\sum_{j=1}^{N} d_j \mathbf{H}_j\right) \left(\mathbf{F}^{\top} \otimes \mathbf{I}_k\right)\right] + \left[\left(\mathbf{F} \otimes \mathbf{I}_k\right) \left(\sum_{j=1}^{N} d_j \mathbf{H}_j\right)^{\top} \left(\mathbf{F}^{\top} \otimes \mathbf{I}_k\right)\right]\right) \mathbf{S}^{\top}\right) \tag{133}$$

$$= \operatorname{tr}\left(\mathbf{Z}^{-1}\mathbf{S}\left[\left(\mathbf{F} \otimes \mathbf{I}_k\right) \left(\sum_{j=1}^{N} d_j \mathbf{H}_j\right) \left(\mathbf{F}^{\top} \otimes \mathbf{I}_k\right)\right] \mathbf{S}^{\top}\right) + \operatorname{tr}\left(\mathbf{Z}^{-1}\mathbf{S}\left[\left(\mathbf{F} \otimes \mathbf{I}_k\right) \left(\sum_{j=1}^{N} d_j \mathbf{H}_j\right)^{\top} \left(\mathbf{F}^{\top} \otimes \mathbf{I}_k\right)\right] \mathbf{S}^{\top}\right) \tag{134}$$

$$= \operatorname{tr}\left(\mathbf{Z}^{-1}\mathbf{S}\left[\left(\mathbf{F} \otimes \mathbf{I}_k\right) \left(\sum_{j=1}^{N} d_j \mathbf{H}_j\right) \left(\mathbf{F}^{\top} \otimes \mathbf{I}_k\right)\right] \mathbf{S}^{\top}\right) + \operatorname{tr}\left(\mathbf{S}\left[\left(\mathbf{F} \otimes \mathbf{I}_k\right) \left(\sum_{j=1}^{N} d_j \mathbf{H}_j\right) \left(\mathbf{F}^{\top} \otimes \mathbf{I}_k\right)\right] \mathbf{S}^{\top}\mathbf{Z}^{-1}\right) \tag{135}$$

$$= \operatorname{tr}\left(\mathbf{Z}^{-1}\mathbf{S}\left[\left(\mathbf{F} \otimes \mathbf{I}_k\right) \left(\sum_{j=1}^{N} d_j \mathbf{H}_j\right) \left(\mathbf{F}^{\top} \otimes \mathbf{I}_k\right)\right] \mathbf{S}^{\top}\right) + \operatorname{tr}\left(\mathbf{Z}^{-1}\mathbf{S}\left[\left(\mathbf{F} \otimes \mathbf{I}_k\right) \left(\sum_{j=1}^{N} d_j \mathbf{H}_j\right) \left(\mathbf{F}^{\top} \otimes \mathbf{I}_k\right)\right] \mathbf{S}^{\top}\right) \tag{136}$$

$$= 2\operatorname{tr}\left(\mathbf{Z}^{-1}\mathbf{S}\left[\left(\mathbf{F} \otimes \mathbf{I}_k\right) \left(\sum_{j=1}^{N} d_j \mathbf{H}_j\right) \left(\mathbf{F}^{\top} \otimes \mathbf{I}_k\right)\right] \mathbf{S}^{\top}\right). \tag{137}$$

Thus, our efforts to find an efficient expression for the second trace lead us to finding an efficient expression for $\sum_{j=1}^{N} d_j \mathbf{H}_j$. Note here that:

$$\sum_{j=1}^{N} d_j \mathbf{H}_j = \left(\text{diag}\left(\left\{\mathbf{C}_G^\top \mathbf{F}\right\}_{f\bullet}\right) \otimes \mathbf{I}_k\right) \mathbf{B} \left(\text{diag}\left(\{\mathbf{F}\}_{t\bullet}\right) \otimes \mathbf{I}_k\right), \tag{138}$$

with

$$\text{diag}\left(\left\{\mathbf{C}_G^\top \mathbf{F}\right\}_{f\bullet}\right) = \begin{pmatrix} \left\{\mathbf{C}_G^\top \mathbf{F}\right\}_{f1} & & 0 \\ & \ddots & \\ 0 & & \left\{\mathbf{C}_G^\top \mathbf{F}\right\}_{fT} \end{pmatrix}, \tag{139}$$

$$\text{diag}\left(\{\mathbf{F}\}_{t\bullet}\right) = \begin{pmatrix} F_{t1} & & 0 \\ & \ddots & \\ 0 & & F_{tT} \end{pmatrix}, \tag{140}$$

$$\mathbf{B}_G = \begin{pmatrix} \mathbf{B}_{G11} & \dots & \mathbf{B}_{G1T} \\ \vdots & & \vdots \\ \mathbf{B}_{G1T} & \dots & \mathbf{B}_{GTT} \end{pmatrix}, \tag{141}$$

$$\mathbf{B}_{Gst} = \mathbf{X}^\top \mathbf{D}\mathbf{D}_s^\dagger \mathbf{D}_t^\dagger \mathbf{X}. \tag{142}$$

Therefore,

$$\text{tr}\left(\mathbf{Z}^{-1}\left(\sum_{j=1}^{N} \mathbf{X}_j^\top \mathbf{V}_j^{-1} \frac{\partial \mathbf{V}_j}{\partial \gamma} \mathbf{V}_j^{-1} \mathbf{X}_j\right)\right) \tag{143}$$

$$= 2\text{tr}\left(\mathbf{Z}^{-1}\mathbf{S}\left[(\mathbf{F} \otimes \mathbf{I}_k)\left(\left(\text{diag}\left(\left\{\mathbf{C}_G^\top \mathbf{F}\right\}_{f\bullet}\right) \otimes \mathbf{I}_k\right) \mathbf{B}_G \left(\text{diag}\left(\{\mathbf{F}\}_{t\bullet}\right) \otimes \mathbf{I}_k\right)\right)\left(\mathbf{F}^\top \otimes \mathbf{I}_k\right)\right] \mathbf{S}^\top\right). \tag{144}$$

With our previously defined binary $K \times Tk$ selector matrix $\mathbf{S}$, we can now rewrite our trace as follows:

$$\text{tr}\left(\mathbf{Z}^{-1}\left(\sum_{j=1}^{N} \mathbf{X}_j^\top \mathbf{V}_j^{-1} \frac{\partial \mathbf{V}_j}{\partial \gamma} \mathbf{V}_j^{-1} \mathbf{X}_j\right)\right) \tag{145}$$

$$= 2\text{tr}\left(\left(\left(\text{diag}\left(\left\{\mathbf{C}_G^\top \mathbf{F}\right\}_{f\bullet}\right) \otimes \mathbf{I}_k\right) \mathbf{B}_G \left(\text{diag}\left(\{\mathbf{F}\}_{t\bullet}\right) \otimes \mathbf{I}_k\right)\left[\left(\mathbf{F}^\top \otimes \mathbf{I}_k\right) \mathbf{S}^\top \mathbf{Z}^{-1} \mathbf{S} \left(\mathbf{F} \otimes \mathbf{I}_k\right)\right]\right). \tag{146}$$

In the last expression, notice that $\operatorname{diag}\left(\left\{\mathbf{C}_G^\top \mathbf{F}\right\}_{f\bullet}\right) \otimes \mathbf{I}_k$ and $\operatorname{diag}\left(\{\mathbf{F}\}_{t\bullet}\right) \otimes \mathbf{I}_k$ are both diagonal matrices. Therefore, by properties of the Hadamard product, we have that:

$$\operatorname{tr}\left(\mathbf{Z}^{-1}\left(\sum_{j=1}^{N}\mathbf{X}_j^\top \mathbf{V}_j^{-1}\frac{\partial \mathbf{V}_j}{\partial \gamma}\mathbf{V}_j^{-1}\mathbf{X}_j\right)\right) = 2\left(\left\{\mathbf{C}_G^\top \mathbf{F}\right\}_{f\bullet}^{\top} \otimes \boldsymbol{\iota}_{1\times k}\right)\left[\mathbf{B}_G \circ \mathbf{J}\right]\left(\{\mathbf{F}\}_{t\bullet} \otimes \boldsymbol{\iota}_{k\times 1}\right) \tag{147}$$

$$= \left\{2\left(\mathbf{C}_G^\top \mathbf{F} \otimes \boldsymbol{\iota}_{1\times k}\right)\left[\mathbf{B}_G \circ \mathbf{J}\right]\left(\mathbf{F}^\top \otimes \boldsymbol{\iota}_{k\times 1}\right)\right\}_{ft}, \text{ where} \tag{148}$$

$$\mathbf{J} = \left(\mathbf{F}^\top \otimes \mathbf{I}_k\right)\mathbf{S}^\top \mathbf{Z}^{-1}\mathbf{S}\left(\mathbf{F} \otimes \mathbf{I}_k\right), \tag{149}$$

$$\mathbf{B}_G = \begin{pmatrix} \mathbf{B}_{G11} & \dots & \mathbf{B}_{G1T} \\ \vdots & & \vdots \\ \mathbf{B}_{G1T} & \dots & \mathbf{B}_{GTT} \end{pmatrix}, \text{ and} \tag{150}$$

$$\mathbf{B}_{Gst} = \mathbf{X}^\top \mathbf{D}\mathbf{D}_s^\dagger \mathbf{D}_t^\dagger \mathbf{X}. \tag{151}$$

Therefore, to determine the second trace, we need to compute our grand $Tk \times Tk$ matrix $\mathbf{B}_G$. This calculation can be done in $O\left(NT^2\right)$ time. Next, we need to compute the element-wise product of this grand matrix with $\mathbf{J}$, which is readily available from previous steps and computationally trivial. Finally, we need to pre-multiply the resultant matrix by the $F_G \times Tk$ matrix $\mathbf{C}_G^\top \mathbf{F} \otimes \boldsymbol{\iota}_{1\times k}$ and post-multiply by the $Tk \times T$ matrix $\mathbf{F}^\top \otimes \boldsymbol{\iota}_{k\times 1}$, where F_G denotes the number of genetic factors in our model. These two multiplications can be carried out in $O\left(T^3\right)$ time. Thus, overall, the second trace can be computed in $O\left(NT^2\right)$ time, provided $k = O(1)$ and thereby $K = O(T)$.

Analogously, for ε, the effect of environmental factor f on trait t, we have that:

$$\operatorname{tr}\left(\mathbf{Z}^{-1}\left(\sum_{j=1}^{N}\mathbf{X}_j^\top \mathbf{V}_j^{-1}\frac{\partial \mathbf{V}_j}{\partial \varepsilon}\mathbf{V}_j^{-1}\mathbf{X}_j\right)\right) = \left\{2\left(\mathbf{C}_E^\top \mathbf{F} \otimes \boldsymbol{\iota}_{1\times k}\right)\left[\mathbf{B}_E \circ \mathbf{J}\right]\left(\mathbf{F}^\top \otimes \boldsymbol{\iota}_{k\times 1}\right)\right\}_{ft}, \text{ where} \tag{152}$$

$$\mathbf{B}_E = \begin{pmatrix} \mathbf{B}_{E11} & \dots & \mathbf{B}_{E1T} \\ \vdots & & \vdots \\ \mathbf{B}_{E1T} & \dots & \mathbf{B}_{ETT} \end{pmatrix} \text{ and} \tag{153}$$

$$\mathbf{B}_{Est} = \mathbf{X}^\top \mathbf{D}_s^\dagger \mathbf{D}_t^\dagger \mathbf{X}. \tag{154}$$

Notice that in case of identical covariates across traits, $\mathbf{J}$ as defined in Eq 149 reduces to $\mathbf{J}$ as defined in Eq 53. When $\mathbf{S} = \mathbf{I}_{Tk}$, we can substitute $\mathbf{Z}^{-1}$ in Eq. 148 by the efficient expressions in Eq. 52. Doing so

yields:

$$\operatorname{tr}\left(\mathbf{Z}^{-1}\left(\sum_{j=1}^{N}\mathbf{X}_j^{\top}\mathbf{V}_j^{-1}\frac{\partial \mathbf{V}_j}{\partial \gamma}\mathbf{V}_j^{-1}\mathbf{X}_j\right)\right) \tag{155}$$

$$= \left\{ 2\left(\mathbf{C}_G^{\top}\mathbf{F}\otimes\boldsymbol{\iota}_{1\times k}\right)\left[\begin{pmatrix} \mathbf{B}_{G11} & \dots & \mathbf{B}_{G1T} \\ \vdots & & \vdots \\ \mathbf{B}_{G1T} & \dots & \mathbf{B}_{GTT}\end{pmatrix}\circ\right.\right. \tag{156}$$

$$\left.\left.\left(\left(\mathbf{F}^{\top}\otimes\mathbf{I}_k\right)\left(\left(\mathbf{F}^{-1}\right)^{\top}\otimes\mathbf{I}_k\right)\begin{bmatrix}\mathbf{B}_1^{-1} & & \mathbf{0} \\ & \ddots & \\ \mathbf{0} & & \mathbf{B}_T^{-1}\end{bmatrix}\left(\mathbf{F}^{-1}\otimes\mathbf{I}_k\right)\left(\mathbf{F}\otimes\mathbf{I}_k\right)\right)\right]\left(\mathbf{F}^{\top}\otimes\boldsymbol{\iota}_{k\times 1}\right)\right\}_{ft} \tag{157}$$

$$= \left\{ 2\left(\mathbf{C}_G^{\top}\mathbf{F}\otimes\boldsymbol{\iota}_{1\times k}\right)\left[\begin{pmatrix} \mathbf{B}_{G11} & \dots & \mathbf{B}_{G1T} \\ \vdots & & \vdots \\ \mathbf{B}_{G1T} & \dots & \mathbf{B}_{GTT}\end{pmatrix}\circ\begin{bmatrix}\mathbf{B}_1^{-1} & & \mathbf{0} \\ & \ddots & \\ \mathbf{0} & & \mathbf{B}_T^{-1}\end{bmatrix}\right]\left(\mathbf{F}^{\top}\otimes\boldsymbol{\iota}_{k\times 1}\right)\right\}_{ft} \tag{158}$$

$$= \left\{ 2\left(\mathbf{C}_G^{\top}\mathbf{F}\otimes\boldsymbol{\iota}_{1\times k}\right)\begin{bmatrix}\mathbf{B}_1^{-1}\circ\mathbf{B}_{G11} & & \mathbf{0} \\ & \ddots & \\ \mathbf{0} & & \mathbf{B}_T^{-1}\circ\mathbf{B}_{GTT}\end{bmatrix}\left(\mathbf{F}^{\top}\otimes\boldsymbol{\iota}_{k\times 1}\right)\right\}_{ft} \tag{159}$$

$$= \left\{ 2\mathbf{C}_G^{\top}\mathbf{F}\begin{bmatrix}\boldsymbol{\iota}_{1\times k}\left(\mathbf{B}_1^{-1}\circ\mathbf{B}_{G11}\right)\boldsymbol{\iota}_{k\times 1} & & \mathbf{0} \\ & \ddots & \\ \mathbf{0} & & \boldsymbol{\iota}_{1\times k}\left(\mathbf{B}_T^{-1}\circ\mathbf{B}_{GTT}\right)\boldsymbol{\iota}_{k\times 1}\end{bmatrix}\mathbf{F}^{\top}\right\}_{ft} \tag{160}$$

$$= \left\{ 2\mathbf{F}\begin{bmatrix}\boldsymbol{\iota}_{1\times k}\left(\mathbf{B}_1^{-1}\circ\mathbf{B}_{G11}\right)\boldsymbol{\iota}_{k\times 1} & & \mathbf{0} \\ & \ddots & \\ \mathbf{0} & & \boldsymbol{\iota}_{1\times k}\left(\mathbf{B}_T^{-1}\circ\mathbf{B}_{GTT}\right)\boldsymbol{\iota}_{k\times 1}\end{bmatrix}\mathbf{F}^{\top}\mathbf{C}_G\right\}_{tf}. \tag{161}$$

By virtue of the symmetry of both $\mathbf{B}_t^{-1}$ and $\mathbf{B}_{Gtt}$, we get that:

$$\boldsymbol{\iota}_{1\times k}\left(\mathbf{B}_t^{-1}\circ\mathbf{B}_{Gtt}\right)\boldsymbol{\iota}_{k\times 1} = \operatorname{tr}\left(\mathbf{B}_t^{-1}\mathbf{B}_{Gtt}\right). \tag{162}$$

Thus, in case of identical covariates across traits, we have that:

$$\operatorname{tr}\left(\mathbf{Z}^{-1}\left(\sum_{j=1}^{N}\mathbf{X}_j^{\top}\mathbf{V}_j^{-1}\frac{\partial\mathbf{V}_j}{\partial\gamma}\mathbf{V}_j^{-1}\mathbf{X}_j\right)\right)$$
$$=\left\{2\mathbf{F}\begin{bmatrix}\operatorname{tr}\left(\mathbf{B}_1^{-1}\mathbf{B}_{G11}\right) & & \mathbf{0}\\ & \ddots & \\ \mathbf{0} & & \operatorname{tr}\left(\mathbf{B}_T^{-1}\mathbf{B}_{GTT}\right)\end{bmatrix}\mathbf{F}^{\top}\mathbf{C}_G\right\}_{tf}.$$

Analogously, for ε, the effect of environmental factor f on trait t, we can derive that:

$$\operatorname{tr}\left(\mathbf{Z}^{-1}\left(\sum_{j=1}^{N}\mathbf{X}_j^{\top}\mathbf{V}_j^{-1}\frac{\partial\mathbf{V}_j}{\partial\varepsilon}\mathbf{V}_j^{-1}\mathbf{X}_j\right)\right) \tag{163}$$
$$=\left\{2\mathbf{F}\begin{bmatrix}\operatorname{tr}\left(\mathbf{B}_1^{-1}\mathbf{B}_{E11}\right) & & \mathbf{0}\\ & \ddots & \\ \mathbf{0} & & \operatorname{tr}\left(\mathbf{B}_T^{-1}\mathbf{B}_{ETT}\right)\end{bmatrix}\mathbf{F}^{\top}\mathbf{C}_E\right\}_{tf}. \tag{164}$$

Overall, computing the second trace can be done in $O\left(NT^2\right)$ time when covariates differ across traits and in $O\left(NT\right)$ time in case of identical covariates across traits. These statements about the computational order rely on $k = O\left(1\right)$.

Overall complexity of the calculation of the gradient. We can compute the gradient in $O\left(NT^2\right)$ time, provided $k = O\left(1\right)$. Notice again, however, that having no covariates at all is numerically easier in terms of the degree to which the problem scales with $O\left(NT^2\right)$. The reason is that many terms can be ignored altogether (e.g., $\log|\mathbf{Z}|$) in a model without covariates.

Step 8. Calculating the AI matrix efficiently

From the expression of the AI matrix of the log-likelihood in Eq. 24, it follows that we need efficient expressions for weighted squared sum of residuals for each combination of parameters.

First squared sum of residuals in the AI matrix. Here, we need to find an efficient expression for $\sum_{j=1}^{N}\mathbf{r}_j^{\top}\frac{\partial\mathbf{V}_j}{\partial\theta_1}\mathbf{V}_j^{-1}\frac{\partial\mathbf{V}_j}{\partial\theta_2}\mathbf{r}_j$. Central in this derivation is finding an efficient expression for $\frac{\partial\mathbf{V}_j}{\partial\theta_2}\mathbf{r}_j$. For a genetic

parameter γ and environmental parameter ε, we have:

$$\frac{\partial \mathbf{V}_j}{\partial \gamma}\mathbf{r}_j = d_j \frac{\partial \mathbf{V}_G}{\partial \gamma}\mathbf{r}_j \tag{165}$$

$$= d_j \left[\begin{pmatrix} \mathbf{0}_{T\times(t-1)} & \mathbf{c}_{Gf} & \mathbf{0}_{T\times(T-t)} \end{pmatrix} + \begin{pmatrix} \mathbf{0}_{(t-1)\times T} \\ \mathbf{c}_{Gf}^\top \\ \mathbf{0}_{(T-t)\times T} \end{pmatrix} \right] \mathbf{r}_j \tag{166}$$

$$= d_j \left[\mathbf{c}_{Gf} R_{tj} + \begin{pmatrix} \mathbf{0}_{(t-1)\times 1} \\ \mathbf{c}_{Gf}^\top \mathbf{r}_j \\ \mathbf{0}_{(T-t)\times 1} \end{pmatrix} \right] \text{ and} \tag{167}$$

$$\frac{\partial \mathbf{V}_j}{\partial \varepsilon}\mathbf{r}_j = \left[\mathbf{c}_{Ef} R_{tj} + \begin{pmatrix} \mathbf{0}_{(t-1)\times 1} \\ \mathbf{c}_{Ef}^\top \mathbf{r}_j \\ \mathbf{0}_{(T-t)\times 1} \end{pmatrix} \right]. \tag{168}$$

With θ_1 being a genetic parameter, λ, denoting the effect of genetic factor g on trait u, and θ_2 being a genetic parameter, γ, denoting the effect of genetic factor f on trait t, we have that:

$$\sum_{j=1}^{N} \mathbf{r}_j^\top \frac{\partial \mathbf{V}_j}{\partial \theta_1} \mathbf{V}_j^{-1} \frac{\partial \mathbf{V}_j}{\partial \theta_2} \mathbf{r}_j = \sum_{j=1}^{N} \mathbf{r}_j^\top \frac{\partial \mathbf{V}_j}{\partial \lambda} \mathbf{V}_j^{-1} \frac{\partial \mathbf{V}_j}{\partial \gamma} \mathbf{r}_j \tag{169}$$

$$= \sum_{j=1}^{N} d_j^2 \left[\mathbf{c}_{Gg}^\top R_{uj} + \begin{pmatrix} \mathbf{0}_{1\times(u-1)} & \mathbf{c}_{Gg}^\top \mathbf{r}_j & \mathbf{0}_{1\times(T-u)} \end{pmatrix} \right] \mathbf{V}_j^{-1} \left[\mathbf{c}_{Gf} R_{tj} + \begin{pmatrix} \mathbf{0}_{(t-1)\times 1} \\ \mathbf{c}_{Gf}^\top \mathbf{r}_j \\ \mathbf{0}_{(T-t)\times 1} \end{pmatrix} \right] \tag{170}$$

$$= \sum_{j=1}^{N} d_j^2 \Bigg[\left(\mathbf{c}_{Gg}^\top \mathbf{V}_j^{-1} \mathbf{c}_{Gf} R_{uj} R_{tj} \right) + \left(\begin{pmatrix} \mathbf{0}_{1\times(u-1)} & \mathbf{c}_{Gg}^\top \mathbf{r}_j & \mathbf{0}_{1\times(T-u)} \end{pmatrix} \mathbf{V}_j^{-1} \mathbf{c}_{Gf} R_{tj} \right) \tag{171}$$

$$+ \mathbf{c}_{Gg}^\top \mathbf{V}_j^{-1} \begin{pmatrix} \mathbf{0}_{(t-1)\times 1} \\ \mathbf{c}_{Gf}^\top \mathbf{r}_j \\ \mathbf{0}_{(T-t)\times 1} \end{pmatrix} R_{uj} + \begin{pmatrix} \mathbf{0}_{1\times(u-1)} & \mathbf{c}_{Gg}^\top \mathbf{r}_j & \mathbf{0}_{1\times(T-u)} \end{pmatrix} \mathbf{V}_j^{-1} \begin{pmatrix} \mathbf{0}_{(t-1)\times 1} \\ \mathbf{c}_{Gf}^\top \mathbf{r}_j \\ \mathbf{0}_{(T-t)\times 1} \end{pmatrix} \Bigg] \tag{172}$$

$$= \sum_{j=1}^{N} d_j^2 \Big[\left\{ \mathbf{C}_G^\top \mathbf{V}_j^{-1} \mathbf{C}_G \right\}_{gf} R_{uj} R_{tj} + \left\{ \mathbf{C}_G^\top \mathbf{R} \right\}_{gj} \left\{ \mathbf{V}_j^{-1} \mathbf{C}_G \right\}_{uf} R_{tj} \tag{173}$$

$$+ \left\{ \mathbf{C}_G^\top \mathbf{R} \right\}_{fj} \left\{ \mathbf{V}_j^{-1} \mathbf{C}_G \right\}_{tg} R_{uj} + \left\{ \mathbf{V}_j^{-1} \right\}_{ut} \left\{ \mathbf{C}_G^\top \mathbf{R} \right\}_{gj} \left\{ \mathbf{C}_G^\top \mathbf{R} \right\}_{fj} \Big]. \tag{174}$$

Similarly, with θ_1 being an environmental parameter, ν, denoting the effect of environmental factor g on trait u, and θ_2 being a genetic parameter, γ, denoting the effect of genetic factor f on trait t, we have that:

$$\sum_{j=1}^{N} \mathbf{r}_j^\top \frac{\partial \mathbf{V}_j}{\partial \theta_1} \mathbf{V}_j^{-1} \frac{\partial \mathbf{V}_j}{\partial \theta_2} \mathbf{r}_j = \sum_{j=1}^{N} \mathbf{r}_j^\top \frac{\partial \mathbf{V}_j}{\partial \nu} \mathbf{V}_j^{-1} \frac{\partial \mathbf{V}_j}{\partial \gamma} \mathbf{r}_j \tag{175}$$

$$= \sum_{j=1}^{N} d_j \Big[\left\{ \mathbf{C}_E^\top \mathbf{V}_j^{-1} \mathbf{C}_G \right\}_{gf} R_{uj} R_{tj} + \left\{ \mathbf{C}_E^\top \mathbf{R} \right\}_{gj} \left\{ \mathbf{V}_j^{-1} \mathbf{C}_G \right\}_{uf} R_{tj} \tag{176}$$

$$+ \left\{ \mathbf{C}_G^\top \mathbf{R} \right\}_{fj} \left\{ \mathbf{V}_j^{-1} \mathbf{C}_E \right\}_{tg} R_{uj} + \left\{ \mathbf{V}_j^{-1} \right\}_{ut} \left\{ \mathbf{C}_E^\top \mathbf{R} \right\}_{gj} \left\{ \mathbf{C}_G^\top \mathbf{R} \right\}_{fj} \Big]. \tag{177}$$

Finally, for θ_1 being an environmental parameter, ν, denoting the effect of environmental factor g on trait u, and θ_2 being an environmental parameter, ε, denoting the effect of environmental factor f on trait t, we

have that:

$$\sum_{j=1}^{N}\mathbf{r}_j^\top\frac{\partial\mathbf{V}_j}{\partial\theta_1}\mathbf{V}_j^{-1}\frac{\partial\mathbf{V}_j}{\partial\theta_2}\mathbf{r}_j=\sum_{j=1}^{N}\mathbf{r}_j^\top\frac{\partial\mathbf{V}_j}{\partial\nu}\mathbf{V}_j^{-1}\frac{\partial\mathbf{V}_j}{\partial\varepsilon}\mathbf{r}_j \tag{178}$$

$$=\sum_{j=1}^{N}\left[\left\{\mathbf{C}_E^\top\mathbf{V}_j^{-1}\mathbf{C}_E\right\}_{gf}R_{uj}R_{tj}+\left\{\mathbf{C}_E^\top\mathbf{R}\right\}_{gj}\left\{\mathbf{V}_j^{-1}\mathbf{C}_E\right\}_{uf}R_{tj}\right. \tag{179}$$

$$\left.+\left\{\mathbf{C}_E^\top\mathbf{R}\right\}_{fj}\left\{\mathbf{V}_j^{-1}\mathbf{C}_E\right\}_{tg}R_{uj}+\left\{\mathbf{V}_j^{-1}\right\}_{ut}\left\{\mathbf{C}_E^\top\mathbf{R}\right\}_{gj}\left\{\mathbf{C}_E^\top\mathbf{R}\right\}_{fj}\right]. \tag{180}$$

While calculating the log-likelihood, we already obtained $\mathbf{R}$. Moreover, matrices $\mathbf{C}_E^\top\mathbf{R}$ and $\mathbf{C}_G^\top\mathbf{R}$ in the three preceding expressions both need to be computed only once. This calculation can be done in $O\left(NT^2\right)$ time. Thus, the computationally most intensive steps here are obtaining matrices $\mathbf{V}_j^{-1}\mathbf{C}_G$, $\mathbf{V}_j^{-1}\mathbf{C}_E$, $\mathbf{C}_G^\top\mathbf{V}_j^{-1}\mathbf{C}_G$, $\mathbf{C}_E^\top\mathbf{V}_j^{-1}\mathbf{C}_G$, and $\mathbf{C}_E^\top\mathbf{V}_j^{-1}\mathbf{C}_E$. Each of these matrix multiplications can be carried out in at most $O\left(T^3\right)$ time. Thus, across observations, carrying out all these matrix multiplications for $j=1,\ldots,N$ can be done in $O\left(NT^3\right)$ time.

Bearing all this in mind, we can initialise a $P\times P$ matrix $\overline{\boldsymbol{\mathcal{I}}}^*$ with P denoting the number of free parameters in our model. Here, it holds that $P=O\left(T^2\right)$. For each given observation, j, and a given combination of parameters, $\{\theta_1,\theta_2\}$, we can add $\mathbf{r}_j^\top\frac{\partial\mathbf{V}_j}{\partial\theta_1}\mathbf{V}_j^{-1}\frac{\partial\mathbf{V}_j}{\partial\theta_2}\mathbf{r}_j$ to the appropriate element in $\overline{\boldsymbol{\mathcal{I}}}^*$. In this fashion, we can calculate $\sum_{j=1}^{N}\mathbf{r}_j^\top\frac{\partial\mathbf{V}_j}{\partial\theta_1}\mathbf{V}_j^{-1}\frac{\partial\mathbf{V}_j}{\partial\theta_2}\mathbf{r}_j$ for all combinations of parameters in $O\left(NT^4\right)$ time.

Second squared sum of residuals in the AI matrix. Here, we need to find an efficient expression for $\left(\sum_{j=1}^{N}\mathbf{r}_j^\top\frac{\partial\mathbf{V}_j}{\partial\theta_1}\mathbf{V}_j^{-1}\mathbf{X}_j\right)\mathbf{Z}^{-1}\left(\sum_{j=1}^{N}\mathbf{X}_j^\top\mathbf{V}_j^{-1}\frac{\partial\mathbf{V}_j}{\partial\theta_2}\mathbf{r}\right)$. In case of identical covariates across traits, this term can be rewritten as:

$$\left(\sum_{j=1}^{N}\mathbf{r}_j^\top\frac{\partial\mathbf{V}_j}{\partial\theta_1}\mathbf{V}_j^{-1}\mathbf{X}_j\right)\mathbf{Z}^{-1}\left(\sum_{j=1}^{N}\mathbf{X}_j^\top\mathbf{V}_j^{-1}\frac{\partial\mathbf{V}_j}{\partial\theta_2}\mathbf{r}\right) \tag{181}$$

$$=\left(\sum_{j=1}^{N}\mathbf{r}_j^\top\frac{\partial\mathbf{V}_j}{\partial\theta_1}\mathbf{F}\mathbf{D}_j^*\mathbf{F}^\top\mathbf{X}_j\right)\mathbf{Z}^{-1}\left(\sum_{j=1}^{N}\mathbf{X}_j^\top\mathbf{F}\mathbf{D}_j^*\mathbf{F}^\top\frac{\partial\mathbf{V}_j}{\partial\theta_2}\mathbf{r}_j\right) \tag{182}$$

$$=\left(\sum_{j=1}^{N}\mathbf{r}_j^\top\frac{\partial\mathbf{V}_j}{\partial\theta_1}\mathbf{F}\mathbf{D}_j^*\mathbf{F}^\top\mathbf{X}_j\left(\left(\mathbf{F}^{-1}\right)^\top\otimes\mathbf{I}_k\right)\right)\mathbf{J}\left(\sum_{j=1}^{N}\left(\mathbf{F}^{-1}\otimes\mathbf{I}_k\right)\mathbf{X}_j^\top\mathbf{F}\mathbf{D}_j^*\mathbf{F}^\top\frac{\partial\mathbf{V}_j}{\partial\theta_2}\mathbf{r}_j\right). \tag{183}$$

Substituting $\mathbf{F}$ by $\mathbf{F}\otimes\mathbf{I}_1$ and $\mathbf{X}_j$ by its definition as Kronecker product, we get that:

$$\left(\sum_{j=1}^{N}\mathbf{r}_j^\top\frac{\partial\mathbf{V}_j}{\partial\theta_1}\mathbf{V}_j^{-1}\mathbf{X}_j\right)\mathbf{Z}^{-1}\left(\sum_{j=1}^{N}\mathbf{X}_j^\top\mathbf{V}_j^{-1}\frac{\partial\mathbf{V}_j}{\partial\theta_2}\mathbf{r}\right) \tag{184}$$

$$=\left(\sum_{j=1}^{N}\mathbf{r}_j^\top\frac{\partial\mathbf{V}_j}{\partial\theta_1}\mathbf{F}\mathbf{D}_j^*\left(\mathbf{F}^\top\otimes\mathbf{I}_1\right)\left(\mathbf{I}_T\otimes\mathbf{x}_j^\top\right)\left(\left(\mathbf{F}^{-1}\right)^\top\otimes\mathbf{I}_k\right)\right)\mathbf{J}\left(\sum_{j=1}^{N}\left(\mathbf{F}^{-1}\otimes\mathbf{I}_k\right)\left(\mathbf{I}_T\otimes\mathbf{x}_j\right)\left(\mathbf{F}\otimes\mathbf{I}_1\right)\mathbf{D}_j^*\mathbf{F}^\top\frac{\partial\mathbf{V}_j}{\partial\theta_2}\mathbf{r}_j\right) \tag{185}$$

$$=\left(\sum_{j=1}^{N}\mathbf{r}_j^\top\frac{\partial\mathbf{V}_j}{\partial\theta_1}\mathbf{F}\mathbf{D}_j^*\left(\mathbf{I}_T\otimes\mathbf{x}_j^\top\right)\right)\mathbf{J}\left(\sum_{j=1}^{N}\left(\mathbf{I}_T\otimes\mathbf{x}_j\right)\mathbf{D}_j^*\mathbf{F}^\top\frac{\partial\mathbf{V}_j}{\partial\theta_2}\mathbf{r}_j\right). \tag{186}$$

Here, $\mathbf{J}$ is as defined in Eq. 53. For θ_2 being a genetic parameter γ denoting the effect of genetic factor f on trait t, we have:

$$\sum_{j=1}^{N}\left(\mathbf{I}_T\otimes\mathbf{x}_j\right)\mathbf{D}_j^*\mathbf{F}^\top\frac{\partial\mathbf{V}_j}{\partial\gamma}\mathbf{r}_j \tag{187}$$

$$=\begin{pmatrix}\sum_{j=1}^{N}\mathbf{x}_j d_j\left(d_j\lambda_1+1\right)^{-1}\left(\left\{\mathbf{F}^\top\mathbf{C}_G\right\}_{1f}R_{tj}+\{\mathbf{F}\}_{t1}\left\{\mathbf{C}_G^\top\mathbf{R}\right\}_{fj}\right)\\ \vdots\\ \sum_{j=1}^{N}\mathbf{x}_j d_j\left(d_j\lambda_T+1\right)^{-1}\left(\left\{\mathbf{F}^\top\mathbf{C}_G\right\}_{Tf}R_{tj}+\{\mathbf{F}\}_{tT}\left\{\mathbf{C}_G^\top\mathbf{R}\right\}_{fj}\right)\end{pmatrix}. \tag{188}$$

Analogously, for θ_2 being an environmental parameter ε denoting the effect of environmental factor f on trait t, we have:

$$\sum_{j=1}^{N}\left(\mathbf{I}_T\otimes\mathbf{x}_j\right)\mathbf{D}_j^*\mathbf{F}^\top\frac{\partial\mathbf{V}_j}{\partial\varepsilon}\mathbf{r}_j \tag{189}$$

$$=\begin{pmatrix}\sum_{j=1}^{N}\mathbf{x}_j\left(d_j\lambda_1+1\right)^{-1}\left(\left\{\mathbf{F}^\top\mathbf{C}_E\right\}_{1f}R_{tj}+\{\mathbf{F}\}_{t1}\left\{\mathbf{C}_E^\top\mathbf{R}\right\}_{fj}\right)\\ \vdots\\ \sum_{j=1}^{N}\mathbf{x}_j\left(d_j\lambda_T+1\right)^{-1}\left(\left\{\mathbf{F}^\top\mathbf{C}_E\right\}_{Tf}R_{tj}+\{\mathbf{F}\}_{tT}\left\{\mathbf{C}_E^\top\mathbf{R}\right\}_{fj}\right)\end{pmatrix}. \tag{190}$$

When we define the $Tk \times 1$ vectors $\mathbf{w}_\gamma$ and $\mathbf{w}_\varepsilon$ as:

$$\mathbf{w}_\gamma = \begin{pmatrix} \sum_{j=1}^{N} \mathbf{x}_j d_j \left(d_j \lambda_1 + 1\right)^{-1} \left(\left\{ \mathbf{F}^\top \mathbf{C}_G \right\}_{1f} R_{tj} + \left\{ \mathbf{F} \right\}_{t1} \left\{ \mathbf{C}_G^\top \mathbf{R} \right\}_{fj} \right) \\ \vdots \\ \sum_{j=1}^{N} \mathbf{x}_j d_j \left(d_j \lambda_T + 1\right)^{-1} \left(\left\{ \mathbf{F}^\top \mathbf{C}_G \right\}_{Tf} R_{tj} + \left\{ \mathbf{F} \right\}_{tT} \left\{ \mathbf{C}_G^\top \mathbf{R} \right\}_{fj} \right) \end{pmatrix} \text{ and} \tag{191}$$

$$\mathbf{w}_\varepsilon = \begin{pmatrix} \sum_{j=1}^{N} \mathbf{x}_j \left(d_j \lambda_1 + 1\right)^{-1} \left(\left\{ \mathbf{F}^\top \mathbf{C}_E \right\}_{1f} R_{tj} + \left\{ \mathbf{F} \right\}_{t1} \left\{ \mathbf{C}_E^\top \mathbf{R} \right\}_{fj} \right) \\ \vdots \\ \sum_{j=1}^{N} \mathbf{x}_j \left(d_j \lambda_T + 1\right)^{-1} \left(\left\{ \mathbf{F}^\top \mathbf{C}_E \right\}_{Tf} R_{tj} + \left\{ \mathbf{F} \right\}_{tT} \left\{ \mathbf{C}_E^\top \mathbf{R} \right\}_{fj} \right) \end{pmatrix}, \tag{192}$$

we can stack these vectors into a $Tk \times P$ matrix $\mathbf{W}$. Here, P denotes the number of parameters and we need to make sure that the parameters are indexed in the same manner over the columns of $\mathbf{W}$ as over the columns of $\overline{\mathcal{I}}^*$. The second term of the AI matrix across all parameter combinations is then given by $\mathbf{W}^\top \mathbf{J} \mathbf{W}$. Given that we already finalised the calculation of $\overline{\mathcal{I}}^*$ when computing the first squared sum of residuals in the AI matrix, our final AI matrix can be expressed as:

$$\overline{\mathcal{I}} = \frac{1}{2} \left(\overline{\mathcal{I}}^* - \mathbf{W}^\top \mathbf{J} \mathbf{W} \right). \tag{193}$$

However, these vectors $\mathbf{w}$ can be written more efficiently in terms of Hadamard products:

$$\mathbf{w}_\gamma = \operatorname{vec} \left(\mathbf{X}^\top \left[\begin{pmatrix} \frac{d_1}{d_1 \lambda_1 + 1} & \cdots & \frac{d_1}{d_1 \lambda_T + 1} \\ \vdots & \ddots & \vdots \\ \frac{d_N}{d_N \lambda_1 + 1} & \cdots & \frac{d_N}{d_N \lambda_T + 1} \end{pmatrix} \circ \left(\left\{ \mathbf{R} \right\}_{t\bullet} \left\{ \mathbf{C}_G^\top \mathbf{F} \right\}_{f\bullet}^\top + \left\{ \mathbf{C}_G^\top \mathbf{R} \right\}_{f\bullet} \left\{ \mathbf{F} \right\}_{t\bullet}^\top \right) \right] \right) \text{ and} \tag{194}$$

$$\mathbf{w}_\varepsilon = \operatorname{vec} \left(\mathbf{X}^\top \left[\begin{pmatrix} \frac{1}{d_1 \lambda_1 + 1} & \cdots & \frac{1}{d_1 \lambda_T + 1} \\ \vdots & \ddots & \vdots \\ \frac{1}{d_N \lambda_1 + 1} & \cdots & \frac{1}{d_N \lambda_T + 1} \end{pmatrix} \circ \left(\left\{ \mathbf{R} \right\}_{t\bullet} \left\{ \mathbf{C}_E^\top \mathbf{F} \right\}_{f\bullet}^\top + \left\{ \mathbf{C}_E^\top \mathbf{R} \right\}_{f\bullet} \left\{ \mathbf{F} \right\}_{t\bullet}^\top \right) \right] \right). \tag{195}$$

Here, $\left\{ \mathbf{R} \right\}_{t\bullet}$, $\left\{ \mathbf{C}_G^\top \mathbf{F} \right\}_{f\bullet}$, etc., are all column vectors. Thus, the second term in each Hadamard product is the sum of two outer products of pairs of vector. Each pair comprises an $N \times 1$ vector and a $T \times 1$ vector, and therefore the outer products are all of size $N \times T$.

$\mathbf{C}_E^\top \mathbf{F}$, $\mathbf{C}_G^\top \mathbf{F}$, $\mathbf{R}$, $\mathbf{F}$, $\mathbf{C}_E^\top \mathbf{R}$, and $\mathbf{C}_G^\top \mathbf{R}$ and their elements are readily available after calculating the log-likelihood and its gradient. Computing the Hadamard products can be done in $O(NT)$ time. Pre-multiplying

these Hadamard products by $\mathbf{X}^\top$ also requires $O(NT)$ time, provided $k = O(1)$. Vectorisation and pre-multiplication by the block-diagonal matrix is trivial. As this chain of calculations needs to be carried out for all P parameters, where $P = O\left(T^2\right)$, calculating the full matrix $\mathbf{W}$ requires $O\left(NT^3\right)$ time. Finally, we need to compute $\mathbf{W}^\top\mathbf{J}\mathbf{W}$ in $O\left(T^5\right)$ time. Thus, assuming $T < N$, this means the time complexity of the AI matrix lies in computing $\overline{\mathcal{I}}^*$. This computation requires $O\left(NT^4\right)$ time.

Finally, in case the covariates are not identical across traits we have that:

$$\left(\sum_{j=1}^{N}\mathbf{r}_j^\top\frac{\partial\mathbf{V}_j}{\partial\theta_1}\mathbf{V}_j^{-1}\mathbf{X}_j\right)\mathbf{Z}^{-1}\left(\sum_{j=1}^{N}\mathbf{X}_j^\top\mathbf{V}_j^{-1}\frac{\partial\mathbf{V}_j}{\partial\theta_2}\mathbf{r}\right) \tag{196}$$

$$= \left(\sum_{j=1}^{N}\mathbf{r}_j^\top\frac{\partial\mathbf{V}_j}{\partial\theta_1}\mathbf{F}\mathbf{D}_j^*\mathbf{F}^\top\left(\mathbf{I}_T\otimes\mathbf{x}_j^\top\right)\mathbf{S}^\top\right)\mathbf{Z}^{-1}\left(\sum_{j=1}^{N}\mathbf{S}\left(\mathbf{I}_T\otimes\mathbf{x}_j^\top\right)\mathbf{F}\mathbf{D}_j^*\mathbf{F}^\top\frac{\partial\mathbf{V}_j}{\partial\theta_2}\mathbf{r}_j\right) \tag{197}$$

$$= \left(\sum_{j=1}^{N}\mathbf{r}_j^\top\frac{\partial\mathbf{V}_j}{\partial\theta_1}\mathbf{F}\mathbf{D}_j^*\mathbf{F}^\top\left(\mathbf{I}_T\otimes\mathbf{x}_j^\top\right)\right)\mathbf{S}^\top\mathbf{Z}^{-1}\mathbf{S}\left(\sum_{j=1}^{N}\left(\mathbf{I}_T\otimes\mathbf{x}_j\right)\mathbf{F}\mathbf{D}_j^*\mathbf{F}^\top\frac{\partial\mathbf{V}_j}{\partial\theta_2}\mathbf{r}_j\right) \tag{198}$$

$$= \left(\sum_{j=1}^{N}\mathbf{r}_j^\top\frac{\partial\mathbf{V}_j}{\partial\theta_1}\mathbf{F}\mathbf{D}_j^*\left(\mathbf{I}_T\otimes\mathbf{x}_j^\top\right)\right)\left(\mathbf{F}^\top\otimes\mathbf{I}_k\right)\mathbf{S}^\top\mathbf{Z}^{-1}\mathbf{S}\left(\mathbf{F}\otimes\mathbf{I}_k\right)\left(\sum_{j=1}^{N}\left(\mathbf{I}_T\otimes\mathbf{x}_j\right)\mathbf{D}_j^*\mathbf{F}^\top\frac{\partial\mathbf{V}_j}{\partial\theta_2}\mathbf{r}_j\right) \tag{199}$$

$$= \left(\sum_{j=1}^{N}\mathbf{r}_j^\top\frac{\partial\mathbf{V}_j}{\partial\theta_1}\mathbf{F}\mathbf{D}_j^*\left(\mathbf{I}_T\otimes\mathbf{x}_j^\top\right)\right)\mathbf{J}\left(\sum_{j=1}^{N}\left(\mathbf{I}_T\otimes\mathbf{x}_j\right)\mathbf{D}_j^*\mathbf{F}^\top\frac{\partial\mathbf{V}_j}{\partial\theta_2}\mathbf{r}_j\right), \tag{200}$$

where $\mathbf{J}$ is as defined in Eq. 149. When using the definition of $\mathbf{J}$ as in Eq. 149 (rather than in Eq. 53), we get that expressions for the AI matrix with different covariates across traits are the same as expressions for the AI matrix in case of identical covariates.

Overall complexity of the calculation of the AI matrix. The time complexity of calculating the AI matrix is linear in the number of observations, and quadratic in the number of parameters. With $P = O\left(T^2\right)$, the AI matrix can be computed in $O\left(NT^4\right)$ time. The computational complexity thus increases rapidly with the number of traits, but our approach seems to yield the lowest time complexity that can be expected to be attainable. The reason is that the number of parameters in the MGREML model increases quadratically with the number of traits considered. In an information matrix, the number of unique elements increases quadratically with the number of parameters. Therefore, the factor T^4 cannot reasonably be avoided. Similarly, each individual contributes linearly to the AI matrix as well as to its time complexity. Therefore, the factor N cannot reasonably be avoided either.

Step 9. Maximising the likelihood using a BFGS algorithm

To maximise the likelihood function, we use a Broyden–Fletcher–Goldfarb–Shanno (BFGS) algorithm (Nocedal and Wright, 2006). The reason for using a BFGS algorithm instead of a Newton-Raphson algorithm, is that the former only requires evaluations of the likelihood function and its gradient. The latter requires calculation of the AI matrix, which is computationally much more expensive. BFGS is a quasi-Newton method in which each update takes the form:

$$\boldsymbol{\theta}_{k+1} = \boldsymbol{\theta}_k + \alpha_k \mathbf{p}_k. \tag{201}$$

Here, α is the step size of the line search, k is an iteration counter, and

$$\mathbf{p}_k = -\mathbf{B}_k^{-1} \nabla \log l\left(\boldsymbol{\theta}_k\right), \tag{202}$$

is the search direction with $\nabla \log l\left(\boldsymbol{\theta}\right)$ the gradient of $\log l\left(\boldsymbol{\theta}\right)$ and $\mathbf{B}_{k+1}^{-1}$ the approximation of the inverse Hessian, defined by:

$$\mathbf{s}_k = \boldsymbol{\theta}_{k+1} - \boldsymbol{\theta}_k = \alpha_k \mathbf{p}_k, \tag{203}$$

$$\mathbf{d}_k = \nabla \log l\left(\boldsymbol{\theta}_{k+1}\right) - \nabla \log l\left(\boldsymbol{\theta}_k\right), \tag{204}$$

$$\rho_k = (\mathbf{s}_k^\top \mathbf{d}_k)^{-1}, \tag{205}$$

$$\mathbf{B}_{k+1}^{-1} = (\mathbf{I} - \rho_k \mathbf{s}_k \mathbf{d}_k^\top) \mathbf{B}_k^{-1} (\mathbf{I} - \rho_k \mathbf{d}_k \mathbf{s}_k^\top) + \rho_k \mathbf{s}_k \mathbf{s}_k^\top. \tag{206}$$

Using these expressions, the BFGS algorithm can be described as:

1. Given start $\boldsymbol{\theta}_0$, convergence tolerance $\varepsilon > 0$, and $\mathbf{B}_0^{-1} = \mathbf{I}$.
2. $k \leftarrow 0$.
3. While $\|\nabla \log l\left(\boldsymbol{\theta}_k\right)\| > \varepsilon$.
4. Compute search direction $\mathbf{p}_k = -\mathbf{B}_k^{-1} \nabla \log l\left(\boldsymbol{\theta}_k\right)$.
5. Set $\boldsymbol{\theta}_{k+1} = \boldsymbol{\theta}_k + \alpha_k \mathbf{p}_k$, with α_k being obtained with a Golden section line search.
6. Compute B_{k+1}^{-1} as in Eq. 206.
7. $k \leftarrow k + 1$.

8. End while

Step 10. Computing standard errors using a delta method

The estimation procedure returns an estimate of $\boldsymbol{\theta}$ (i.e., an estimate of the factor model that underpins $\mathbf{V}_G$ and $\mathbf{V}_E$). In practice, it is often more useful to investigate the genetic and environmental variance matrices, the genetic and environmental correlation matrices, and the SNP-based heritability (h^2_{SNPs}), which can all be defined in terms of transformations of $\mathbf{V}_G$ and $\mathbf{V}_E$. In this section, the appropriate standard errors for these transformations are derived.

For some function $g(\boldsymbol{\theta})$, the delta method states that when this function is evaluated at the maximum likelihood estimate, $\widehat{\boldsymbol{\theta}}_{\mathrm{ML}}$, the value this function returns is (approximately) distributed as:

$$g(\widehat{\boldsymbol{\theta}}_{\mathrm{ML}}) \sim \mathcal{N}\left(g(\widehat{\boldsymbol{\theta}}_{\mathrm{ML}}), \nabla g(\widehat{\boldsymbol{\theta}}_{\mathrm{ML}})^\top \mathcal{I}^{-1}(\widehat{\boldsymbol{\theta}}_{\mathrm{ML}}) \nabla g(\widehat{\boldsymbol{\theta}}_{\mathrm{ML}})\right). \tag{207}$$

Here, $\nabla g(\boldsymbol{\theta}) = \partial g(\boldsymbol{\theta})/\partial \boldsymbol{\theta}$ is the gradient of $g()$ with respect to $\boldsymbol{\theta}$. In what follows, the functions $g(\boldsymbol{\theta})$ and their gradients are defined to derive the estimates and standard errors of the SNP-based heritability (h^2_{SNPs}), the genetic and environmental correlation matrix, and the genetic and environmental variance-covariance matrix.

Heritability. For h^2_{SNPs} of trait t (denoted by $h^2_{\mathrm{SNPs}}(t)$), we have that:

$$h^2_{\mathrm{SNPs}}(t) = V_{G_t} \left(V_{G_t} + V_{E_t}\right)^{-1} = \frac{V_{G_t}}{V_t}, \tag{208}$$

where V_{G_t} denotes the genetic variance of trait t, V_{E_t} the corresponding environmental variance, and $V_t = V_{G_t} + V_{E_t}$ the total variance of trait t. Using the product rule and the chain rule, we get:

$$\begin{aligned}
\partial h^2_{\mathrm{SNPs}}(t) &= \partial V_{G_t} \left(V_{G_t} + V_{E_t}\right)^{-1} - V_{G_t} \left(V_{G_t} + V_{E_t}\right)^{-2} \left(\partial V_{G_t} + \partial V_{E_t}\right) && (209)\\
&= \frac{\partial V_{G_t}}{V_t} - \frac{V_{G_t}}{V_t} \frac{\partial V_{G_t} + \partial V_{E_t}}{V_t} && (210)\\
&= \frac{\partial V_{G_t}}{V_t} - h_t^2 \frac{\partial V_{G_t} + \partial V_{E_t}}{V_t} && (211)\\
&= \frac{\left(1 - h_t^2\right) \partial V_{G_t} - h_t^2 \partial V_{E_t}}{V_t}. && (212)
\end{aligned}$$

Thus, quite intuitively, when $h^2_{\text{SNPs}}(t) = 0$, changes in V_{E_t} will not affect $h^2_{\text{SNPs}}(t)$ (as long as $V_{G_t} = 0$, the heritability will remain zero). Conversely, when $h^2_{\text{SNPs}}(t) = 1$, changes in V_{G_t} will not affect $h^2_{\text{SNPs}}(t)$ (as long as $V_{E_t} = 0$, the SNP-based heritability remains one). Finally, the larger V_t is, the less $h^2_{\text{SNPs}}(t)$ will be affected by changes in either V_{E_t} or V_{G_t}.

Let $\mathbf{b}_t$ be a $T \times 1$ binary vector with all elements equal to zero except for element t. We then have that:

$$V_{G_t} = \mathbf{b}_t^\top \mathbf{C}_G \mathbf{C}_G^\top \mathbf{b}_t. \tag{213}$$

This expression in turn implies, by the product rule, that:

$$\begin{aligned}
\partial V_{G_t} &= \mathbf{b}_t^\top \left(\partial \mathbf{C}_G \mathbf{C}_G^\top + \mathbf{C}_G \partial \mathbf{C}_G^\top \right) \mathbf{b}_t \\
&= \mathbf{b}_t^\top \partial \mathbf{C}_G \mathbf{C}_G^\top \mathbf{b}_t + \mathbf{b}_t^\top \mathbf{C}_G \left(\partial \mathbf{C}_G \right)^\top \mathbf{b}_t \\
&= \mathbf{b}_t^\top \partial \mathbf{C}_G \mathbf{C}_G^\top \mathbf{b}_t + \left(\mathbf{b}_t^\top \mathbf{C}_G \left(\partial \mathbf{C}_G \right)^\top \mathbf{b}_t \right)^\top \\
&= \mathbf{b}_t^\top \partial \mathbf{C}_G \mathbf{C}_G^\top \mathbf{b}_t + \mathbf{b}_t^\top \partial \mathbf{C}_G \mathbf{C}_G^\top \mathbf{b}_t \\
&= 2\mathbf{b}_t^\top \partial \mathbf{C}_G \mathbf{C}_G^\top \mathbf{b}_t.
\end{aligned}$$

Analogously, we have that:

$$\partial V_{E_t} = 2\mathbf{b}_t^\top \partial \mathbf{C}_E \mathbf{C}_E^\top \mathbf{b}_t. \tag{214}$$

Let γ denote the effect of genetic factor f on trait t. In this case, we have that:

$$\frac{\partial \mathbf{C}_G}{\partial \gamma} = \begin{cases} 1, & \text{for element} \quad t, f \\ 0, & \text{elsewhere.} \end{cases} \tag{215}$$

Thus,

$$\mathbf{b}_t^\top \frac{\partial \mathbf{C}_G}{\partial \gamma} = \begin{pmatrix} \mathbf{0}_{1 \times (f-1)} & 1 & \mathbf{0}_{1 \times (F_G - f)} \end{pmatrix}. \tag{216}$$

This expression reduces to:

$$\mathbf{b}_t^\top \frac{\partial \mathbf{C}_G}{\partial \gamma} \mathbf{C}_G^\top \mathbf{b}_t = \gamma. \tag{217}$$

By means of substituting, we obtain that:

$$\frac{\partial h^2_{\text{SNPs}}(t)}{\partial \gamma} = 2\gamma \frac{1 - h^2_{\text{SNPs}}(t)}{V_t}. \tag{218}$$

Analogously, for the effect ε of some environmental factor f on trait t, we have that:

$$\frac{\partial h^2_{\text{SNPs}}(t)}{\partial \varepsilon} = -2\varepsilon \frac{h^2_{\text{SNPs}}(t)}{V_t}. \tag{219}$$

These parameters can be computed easily for each trait.

Genetic correlation and environmental correlation. The genetic correlation between two traits, t and u, equals:

$$\rho_{G_{tu}} = \frac{\text{Cov}_{G_{tu}}}{\sqrt{V_{G_t} V_{G_u}}}. \tag{220}$$

Here, V_{G_t} and V_{G_u} denote the genetic variance of traits t and u respectively, and $\text{Cov}_{G_{tu}}$ denotes the genetic covariance of t and u. By definition, the correlation is one and the standard error is zero in case $t = u$. Therefore, we focus exclusively on the case where $t \neq u$.

By using the product and chain rule, we get that:

$$\begin{aligned} \partial \rho_{G_{tu}} &= \frac{\partial \text{Cov}_{G_{tu}}}{\sqrt{V_{G_t} V_{G_u}}} - \frac{1}{2} \frac{\text{Cov}_{G_{tu}}}{\sqrt{V_{G_t} V_{G_u}}} \frac{\partial V_{G_t} V_{G_u} + V_{G_t} \partial V_{G_u}}{V_{G_t} V_{G_u}} && (221) \\ &= \frac{\partial \text{Cov}_{G_{tu}}}{\sqrt{V_{G_t} V_{G_u}}} - \frac{1}{2} \rho_{G_{tu}} \frac{\partial V_{G_t} V_{G_u} + V_{G_t} \partial V_{G_u}}{V_{G_t} V_{G_u}}. && (222) \end{aligned}$$

By defining vector $\mathbf{b}_u$ analogously to $\mathbf{b}_t$, substituting expressions found when deriving the gradient of $h^2_{\text{SNPs}}(t)$, and recognising that:

$$\begin{aligned} \text{Cov}_{G_{tu}} &= \mathbf{b}_t^\top \mathbf{C}_G \mathbf{C}_G^\top \mathbf{b}_u \text{ and} && (223) \\ \partial \text{Cov}_{G_{tu}} &= \mathbf{b}_t^\top \partial \mathbf{C}_G \mathbf{C}_G^\top \mathbf{b}_u + \mathbf{b}_u^\top \partial \mathbf{C}_G \mathbf{C}_G^\top \mathbf{b}_t, && (224) \end{aligned}$$

we can derive that:

$$\partial \rho_{G_{tu}} = \frac{\mathbf{b}_t^\top \partial \mathbf{C}_G \mathbf{C}_G^\top \mathbf{b}_u + \mathbf{b}_u^\top \partial \mathbf{C}_G \mathbf{C}_G^\top \mathbf{b}_t}{\sqrt{V_{G_t} V_{G_u}}} - \rho_{G_{tu}} \frac{\mathbf{b}_t^\top \partial \mathbf{C}_G \mathbf{C}_G^\top \mathbf{b}_t V_{G_u} + \mathbf{b}_u^\top \partial \mathbf{C}_G \mathbf{C}_G^\top \mathbf{b}_u V_{G_t}}{V_{G_t} V_{G_u}}. \tag{225}$$

Notice that if $t \neq u$, for a parameter γ that constitutes the genetic effect of factor f on trait t and a parameter λ that constitutes the effect of genetic factor g on trait u, the following holds regardless of whether $f = g$ or $f \neq g$:

$$\frac{\mathbf{b}_t^\top \partial \mathbf{C}_G \mathbf{C}_G^\top \mathbf{b}_t}{\partial \lambda} = 0, \tag{226}$$

$$\frac{\mathbf{b}_u^\top \partial \mathbf{C}_G \mathbf{C}_G^\top \mathbf{b}_u}{\partial \gamma} = 0, \tag{227}$$

$$\frac{\mathbf{b}_t^\top \partial \mathbf{C}_G \mathbf{C}_G^\top \mathbf{b}_u}{\partial \lambda} = 0, \text{ and} \tag{228}$$

$$\frac{\mathbf{b}_u^\top \partial \mathbf{C}_G \mathbf{C}_G^\top \mathbf{b}_t}{\partial \gamma} = 0. \tag{229}$$

Moreover, from the preceding derivations of the gradient of the heritability, we know that:

$$\frac{\mathbf{b}_t^\top \partial \mathbf{C}_G \mathbf{C}_G^\top \mathbf{b}_t}{\partial \gamma} = \gamma, \text{ and} \tag{230}$$

$$\frac{\mathbf{b}_u^\top \partial \mathbf{C}_G \mathbf{C}_G^\top \mathbf{b}_u}{\partial \lambda} = \lambda. \tag{231}$$

Finally, when $f = g$ (i.e., λ and γ correspond to the same genetic factor), we have that:

$$\frac{\mathbf{b}_t^\top \partial \mathbf{C}_G \mathbf{C}_G^\top \mathbf{b}_u}{\partial \gamma} = \lambda, \text{ and} \tag{232}$$

$$\frac{\mathbf{b}_u^\top \partial \mathbf{C}_G \mathbf{C}_G^\top \mathbf{b}_t}{\partial \lambda} = \gamma. \tag{233}$$

Notice that if $f \neq g$, these two partial derivatives are also zero. By substituting expressions, we get for $t \neq u$ and $f = g$:

$$\frac{\partial \rho_{G_{tu}}}{\partial \gamma} = \frac{\lambda}{\sqrt{V_{G_t} V_{G_u}}} - \rho_{G_{tu}} \frac{\gamma}{V_{G_t}}, \text{ and} \tag{234}$$

$$\frac{\partial \rho_{G_{tu}}}{\partial \lambda} = \frac{\gamma}{\sqrt{V_{G_t} V_{G_u}}} - \rho_{G_{tu}} \frac{\lambda}{V_{G_u}}. \tag{235}$$

Either λ or γ may be constrained to zero in the given factor model. Finally, it holds that:

$$\frac{\partial \rho_{G_{tt}}}{\partial \gamma} = 0. \tag{236}$$

By analogy, we obtain that:

$$\frac{\partial \rho_{E_{tu}}}{\partial \varepsilon} = \frac{\nu}{\sqrt{V_{E_t} V_{E_u}}} - \rho_{E_{tu}} \frac{\varepsilon}{V_{E_t}}, \tag{237}$$

$$\frac{\partial \rho_{E_{tu}}}{\partial \nu} = \frac{\varepsilon}{\sqrt{V_{E_t} V_{E_u}}} - \rho_{E_{tu}} \frac{\nu}{V_{E_u}}, \text{ and} \tag{238}$$

$$\frac{\partial \rho_{E_{tt}}}{\partial \varepsilon} = 0. \tag{239}$$

In the first equality, ε is the effect of some environmental factor on trait t and ν is the effect of that same environmental factor on trait u (if any, because ν can be constrained to zero in the given factor model). In the second equality, ν is the effect of some environmental factor on trait u and ε the environmental effect of that same factor on trait t (if any, because, as before, ε can be constrained to zero in the given factor model).

Variance components. For the estimated factor coefficients in $\mathbf{C}_G$ and $\mathbf{C}_E$, the covariance matrix is readily available. However, sometimes one may be interested in the covariance between the elements in $\mathbf{V}_G$ and $\mathbf{V}_E$. That is, one may be interested in the covariance of the variance components rather than in the factor coefficients. For this case, we consider the genetic (respectively environmental) variance of trait t. We have already seen that when γ (ε) denotes the effect of genetic (environmental) factor f on trait t, it holds that:

$$\frac{\partial V_{G_t}}{\partial \gamma} = 2\gamma, \text{ and} \tag{240}$$

$$\frac{\partial V_{E_t}}{\partial \varepsilon} = 2\varepsilon. \tag{241}$$

Thus, gradient vectors for the genetic and environmental variance can be calculated easily. Let's now consider the genetic covariance between traits $t \neq u$, again letting γ denote the effect of genetic factor f on trait t and letting λ denote the effect of that same genetic factor f on trait u. From before, we know that:

$$\frac{\partial \text{Cov}_{G_{tu}}}{\partial \gamma} = \lambda, \text{ and} \tag{242}$$

$$\frac{\partial \text{Cov}_{G_{tu}}}{\partial \lambda} = \gamma. \tag{243}$$

Similarly, for the environmental covariance between $t \neq u$, with ε denoting the effect of environmental factor f on trait t and ν denoting the effect of that same environmental factor on trait u, we have that:

$$\frac{\partial \text{Cov}_{E_{tu}}}{\partial \varepsilon} = \nu, \text{ and} \tag{244}$$

$$\frac{\partial \text{Cov}_{E_{tu}}}{\partial \nu} = \varepsilon. \tag{245}$$

Consequently, gradient vectors of genetic and environmental covariances can also be calculated easily.

Implementation practicalities

Controlling for population stratification. A primary concern in genetic studies is bias resulting from population stratification. To deal with this, a common practice in the GWAS and GREML literature is the inclusion of the lead principal components (PCs) of the GRM as fixed-effect covariates in the model. Instead of using the lead PCs as fixed-effect covariates, which increases the computational burden, MGREML removes the effect of the lead PCs from the data. That is, to control for the K lead PCs, the corresponding K rows of $\mathbf{P}^\top \mathbf{Y}$ are dropped, when applying the canonical transformation. In our software implementation of MGREML, users can set K in accordance with the degree of population stratification in the dataset under consideration. By default, $K = 20$.

Initialising coefficient matrices. To start the optimisation algorithm, starting values for $\mathbf{C}_G$ (Eq. 82) and $\mathbf{C}_E$ (Eq. 83) are required. For a fully saturated model, MGREML sets starting values such that each trait has an initial h^2_{SNPs} of 20% and such that $\mathbf{C}_G\mathbf{C}_G^\top$ and $\mathbf{C}_E\mathbf{C}_E^\top$ are both proportional to a convex combination of the identity matrix and the phenotypic covariance matrix. Here, the phenotypic covariance matrix receives weight 0.999. In case a non-saturated model is specified for $\mathbf{C}_G$, MGREML gives all free elements in row t of $\mathbf{C}_G$ (corresponding to trait t) an equal weight, such that the implied genetic variance of trait t equals 20% of the phenotypic variance in that trait. Thus, also here, the initialisation starts at $h^2_{\text{SNPs}} = 20\%$ (except for the case where a trait has no genetic variance according to the model). An analogous approach is applied when a non-saturated model is specified for $\mathbf{C}_E$. Then, scaling is such that $1 - h^2_{\text{SNPs}} = 80\%$.

Unbalanced data. In case a dataset is unbalanced (i.e., not all traits and/or relevant control variables are available for all individuals in the data), the mathematical complexity of REML estimation increases dramatically. When we consider the full $(NT) \times 1$ phenotype vector $\mathbf{y}$, associated variance matrix $\mathbf{V}$, and matrix of fixed-effect covariates $\widetilde{\mathbf{X}}$, missing data requires us to keep only the subset of the rows of $\widetilde{\mathbf{X}}$ and $\mathbf{y}$

as well as the rows and columns of $\mathbf{V}$ for which both the phenotypic as well as data on the control variables is available. Under balanced data, $\mathbf{V}$ is a block-diagonal matrix with all blocks being of equal size, *viz.*, $T \times T$ (see Eq. 11). Under unbalanced data, the variance-covariance matrix is still block-diagonal. However, the blocks are then no longer necessarily of equal size. Thus, at first sight, we can no longer use our efficient expressions.

Yet, here we show that by including a set of dummy variables to control for missing data we fit we can still apply our computationally efficient expressions. To see this, we first inspect the case of balanced data more closely. By overloading notation from previous parts, we can denote the singular-value decomposition (SVD) of the matrix of fixed-effect covariates by:

$$\widetilde{\mathbf{X}} = \begin{pmatrix} \mathbf{P}_1 & \mathbf{P}_0 \end{pmatrix} \begin{pmatrix} \boldsymbol{\Theta} \\ \mathbf{0} \end{pmatrix} \mathbf{Q}^{\top}. \tag{246}$$

Here, it holds that ${\mathbf{P}_0}^{\top}\widetilde{\mathbf{X}} = \mathbf{0}$. We now define $\mathbf{K} = {\mathbf{P}_0}^{\top}$, and we let $r = \operatorname{rank}\left(\widetilde{\mathbf{X}}\right)$ denote the rank of $\widetilde{\mathbf{X}}$. Then, $\operatorname{rank}(\mathbf{P}_0) = NT - r$. As a result, the REML log-likelihood function in case of balanced data (ignoring the constant), can be described as:

$$l(\boldsymbol{\theta}) = -\frac{1}{2}\log\left(\left|\mathbf{K}\mathbf{V}\mathbf{K}^{\top}\right|\right) - \frac{1}{2}\mathbf{y}^{\top}\mathbf{K}^{\top}\left(\mathbf{K}\mathbf{V}\mathbf{K}^{\top}\right)^{-1}\mathbf{K}\mathbf{y}. \tag{247}$$

Searle et al. (1992) show that:

$$\mathbf{K}^{\top}\left(\mathbf{K}\mathbf{V}\mathbf{K}^{\top}\right)^{-1}\mathbf{K} = \mathbf{V}^{-1} - \mathbf{V}^{-1}\widetilde{\mathbf{X}}\left(\widetilde{\mathbf{X}}^{\top}\mathbf{V}^{-1}\widetilde{\mathbf{X}}\right)^{-1}\widetilde{\mathbf{X}}^{\top}\mathbf{V}^{-1}. \tag{248}$$

This identity allowed us to formulate the log-likelihood in Eq. 12, for which we developed efficient expressions in the preceding parts. Let M now denote the total number of missing values across phenotypes. Then (overloading notation), the $(NT - M) \times (NT)$ binary matrix $\mathbf{S}$ consisting of zeros and ones with precisely a single one per row and at most a single one per column such that $\mathbf{S}\mathbf{S}^{\top} = \mathbf{I}_{NT-M}$ effectively selects the

observations with non-missing data from $\mathbf{y}$. We can use matrix $\mathbf{S}$ to compute:

$$\mathbf{y}^* = \mathbf{S}\mathbf{y}, \tag{249}$$

$$\mathbf{V}^* = \mathbf{S}\mathbf{V}\mathbf{S}^\top, \text{ and} \tag{250}$$

$$\widetilde{\mathbf{X}}^* = \mathbf{S}\widetilde{\mathbf{X}} = \begin{pmatrix} \mathbf{P}_1^* & \mathbf{P}_0^* \end{pmatrix} \begin{pmatrix} \mathbf{\Theta}^* \\ \mathbf{0} \end{pmatrix} \mathbf{Q}^{*\top}. \tag{251}$$

The last expression constitutes the singular value decomposition of $\widetilde{\mathbf{X}}^*$, such that $\mathbf{P}_0^{*\top}\widetilde{\mathbf{X}}^* = \mathbf{0}$. We define $\mathbf{K}^* = \mathbf{P}_0^{*\top}$. With $r = \text{rank}\left(\widetilde{\mathbf{X}}^*\right)$ denoting the rank of $\widetilde{\mathbf{X}}^*$, $\text{rank}\left(\mathbf{P}_0^*\right) = NT - M - r$. Therefore, the REML log-likelihood function in case of unbalanced data (again ignoring the constant) can be described as:

$$l(\boldsymbol{\theta}) = -\frac{1}{2}\log\left(\left|\mathbf{K}^*\mathbf{V}^*\mathbf{K}^{*\top}\right|\right) - \frac{1}{2}\mathbf{y}^{*\top}\mathbf{K}^{*\top}\left(\mathbf{K}^*\mathbf{V}^*\mathbf{K}^{*\top}\right)^{-1}\mathbf{K}^*\mathbf{y}^* \tag{252}$$

$$= -\frac{1}{2}\log\left(\left|\mathbf{K}^*\mathbf{S}\mathbf{V}\mathbf{S}^\top\mathbf{K}^{*\top}\right|\right) - \frac{1}{2}\mathbf{y}^\top\mathbf{S}^\top\mathbf{K}^{*\top}\left(\mathbf{K}^*\mathbf{S}\mathbf{V}\mathbf{S}^\top\mathbf{K}^{*\top}\right)^{-1}\mathbf{K}^*\mathbf{S}\mathbf{y}. \tag{253}$$

At first sight, it seems we can no longer apply the identity by Searle et al. (1992). However, $\mathbf{K}^*\mathbf{S}$ in its entirety can be considered as a design matrix, like $\mathbf{K}$. With missing observations coded with an arbitrary value (e.g., zero) and a set of M dummies that code for the missing observations, $\mathbf{K}^*\mathbf{S}$ is orthogonal to $\widetilde{\mathbf{X}}$. Based on this, $\mathbf{S}^\top\mathbf{K}^{*\top}\left(\mathbf{K}^*\mathbf{S}\mathbf{V}\mathbf{S}^\top\mathbf{K}^{*\top}\right)^{-1}\mathbf{K}^*\mathbf{S}$ can be expressed in terms of a projection matrix. This expression allows to express the log-likelihood under missing data as in Eq. 12, for which we have efficient expressions. In this case, the projection matrix is based on $\widetilde{\mathbf{X}}$ and a set of M dummies that code for the missing data.

More formally, we have an $(NT) \times K$ matrix of covariates $\widetilde{\mathbf{X}}$, an $(NT - M) \times (NT)$ selection matrix $\mathbf{S}$ with elements that are either zero or one and with row-sum $\sum_{j=1}^{NT} S_{ij} = 1\,\forall\, i$ and column-sum $\sum_{i=1}^{(NT-M)} S_{ij} \in \{0,1\}\,\forall j$, and we have an $(NT - M) \times (NT - M - r)$ matrix $\mathbf{P}_0^*$ with orthonormal columns (i.e., $\mathbf{P}_0^{*\top}\mathbf{P}_0^* = \mathbf{I}_{NT-M-r}$) that lie in the null-space of the $(NT - M) \times K$ matrix $\widetilde{\mathbf{X}}^* = \mathbf{S}\widetilde{\mathbf{X}}$ such that $\mathbf{P}_0^{*\top}\widetilde{\mathbf{X}}^* = \mathbf{0}_{(NT-M-r)\times K}$. Finally, $\mathbf{M}$ is an $M \times (NT)$ matrix, defined analogously to $\mathbf{S}$ in such a manner that it selects missing observations rather than non-missing observations as $\mathbf{S}$ does. We can now show that $\mathbf{S}\mathbf{S}^\top = \mathbf{I}_{NT-M}$, $\mathbf{M}\mathbf{M}^\top = \mathbf{I}_M$, $\mathbf{S}\mathbf{M}^\top = \mathbf{0}_{(NT-M)\times M}$, $\mathbf{M}\mathbf{S}^\top = \mathbf{0}_{M\times(NT-M)}$, and $\mathbf{S}^\top\mathbf{S} + \mathbf{M}^\top\mathbf{M} = \mathbf{I}_{NT}$.

Theorem 1. *$\widetilde{\boldsymbol{P}_0} = \boldsymbol{S}^\top\boldsymbol{P}_0^*$ lies in the left null space of $\widetilde{\boldsymbol{X}}_M = \left[\widetilde{\boldsymbol{X}},\ \boldsymbol{M}^\top\right]$ and has rank$\left(\widetilde{\boldsymbol{P}_0}\right)$ = rank$(\boldsymbol{P}_0^*) \equiv NT - M - r$.*

Proof.

$$\widetilde{\mathbf{P}_0}^\top \widetilde{\mathbf{X}}_M = \mathbf{P}_0^{\top *}\mathbf{S}\left[\widetilde{\mathbf{X}},\ \mathbf{M}^\top\right] \tag{254}$$

$$= \left[\mathbf{P}_0^{\top *}\mathbf{S}\widetilde{\mathbf{X}},\ \mathbf{P}_0^{\top *}\mathbf{S}\mathbf{M}^\top\right] \tag{255}$$

$$= \left[\mathbf{P}_0^{\top *}\widetilde{\mathbf{X}}^*,\ \mathbf{P}_0^{\top *}\mathbf{0}_{(NT-M)\times M}\right] \tag{256}$$

$$= \mathbf{0}_{(NT-M-r)\times(K+M)}. \tag{257}$$

Hence, $\widetilde{\mathbf{P}_0}$ lies in the null-space of $\widetilde{\mathbf{X}}_M$.

$$\text{rank}\left(\widetilde{\mathbf{P}_0}\right) = \text{rank}\left(\widetilde{\mathbf{P}_0}^\top \widetilde{\mathbf{P}_0}\right) \tag{258}$$

$$= \text{rank}\left(\mathbf{P}_0^{*\top}\mathbf{S}\mathbf{S}^\top \mathbf{P}_0^*\right) \tag{259}$$

$$= \text{rank}\left(\mathbf{P}_0^{*\top}\mathbf{P}_0^*\right) \tag{260}$$

$$= \text{rank}\left(\mathbf{P}_0^*\right) \equiv NT - M - r. \tag{261}$$

Hence, $\text{rank}\left(\widetilde{\mathbf{P}_0}\right) = NT - M - r$. □

Theorem 2. *rank* $\left(\widetilde{\boldsymbol{X}}_M\right) = r + M$ *and, therefore,* $\widetilde{\boldsymbol{P}_0}$ *spans the null space of* $\widetilde{\boldsymbol{X}}_M$.

Proof. $\text{rank}\left(\widetilde{\mathbf{X}}_M\right)$ is the number of independent columns in $\widetilde{\mathbf{X}}_M$. Hence, orthogonalising one subset of columns of $\widetilde{\mathbf{X}}_M$ with respect to another, non-overlapping subset of columns of $\widetilde{\mathbf{X}}_M$ does not change the rank. Letting $\mathbf{I} - \mathbf{M}^\top\left(\mathbf{M}\mathbf{M}^\top\right)^{-1}\mathbf{M} = \mathbf{I} - \mathbf{M}^\top\mathbf{M} = \mathbf{S}^\top\mathbf{S}$ denote the orthogonal projection matrix that removes the collinearity with columns of $\mathbf{M}^\top$, we obtain – based on the orthogonalisation-argument – that,

$$\text{rank}\left(\widetilde{\mathbf{X}}_M\right) = \text{rank}\left(\left[\widetilde{\mathbf{X}},\ \mathbf{M}^\top\right]\right) \tag{262}$$

$$= \text{rank}\left(\left[\mathbf{S}^\top\mathbf{S}\widetilde{\mathbf{X}},\ \mathbf{M}^\top\right]\right) \tag{263}$$

$$= \text{rank}\left(\mathbf{S}^\top\mathbf{S}\widetilde{\mathbf{X}}\right) + \text{rank}\left(\mathbf{M}^\top\right) \tag{264}$$

$$= \text{rank}\left(\widetilde{\mathbf{X}}^\top\mathbf{S}^\top\mathbf{S}\mathbf{S}^\top\mathbf{S}\widetilde{\mathbf{X}}\right) + \text{rank}\left(\mathbf{M}\mathbf{M}^\top\right) \tag{265}$$

$$= \text{rank}\left(\widetilde{\mathbf{X}}^\top\mathbf{S}^\top\mathbf{S}\widetilde{\mathbf{X}}\right) + \text{rank}\left(\mathbf{I}_M\right) \tag{266}$$

$$= \text{rank}\left(\mathbf{S}\widetilde{\mathbf{X}}\right) + M \tag{267}$$

$$= \text{rank}\left(\widetilde{\mathbf{X}}^*\right) + M = r + M. \tag{268}$$

Given that $\widetilde{\mathbf{X}}_M$ is an $(NT) \times (K+M)$ matrix with $NT \gg K+M \geq r+M$ and with rank equal to $r+M$, its null space is spanned by $NT-M-r$ independent columns. $\square$

The preceding two theorems show that $\widetilde{\mathbf{P}_0}^\top = \mathbf{K}^*\mathbf{S}$ can be regarded as a design matrix, for which the following identity holds:

$$\mathbf{S}^\top\mathbf{K}^{*\top}\left(\mathbf{K}^*\mathbf{S}\mathbf{V}\mathbf{S}^\top\mathbf{K}^{*\top}\right)^{-1}\mathbf{K}^*\mathbf{S} = \mathbf{V}^{-1} - \mathbf{V}^{-1}\widetilde{\mathbf{X}}_M\left(\mathbf{X}_M^\top\mathbf{V}^{-1}\widetilde{\mathbf{X}}_M\right)^{-1}\mathbf{X}_M^\top\mathbf{V}^{-1}, \tag{269}$$

with $\widetilde{\mathbf{X}}_M = \left[\widetilde{\mathbf{X}},\ \mathbf{M}^\top\right]$.

These derivations and proofs show that efficient MGREML estimation can still be applied to unbalanced datasets by treating the data as balanced and including dummy variables for missing values as fixed-effect covariates. In other words, the estimates one obtain with MGREML using this approach will be the same when using the computationally more demanding expressions that arise when directly using unbalanced data.

Scale and convergence. Most terms in the log-likelihood function (Eq. 12) scale linearly with the number of individuals N, and therefore we optimise over the log-likelihood divided by N in our software implementation of MGREML. This scaling by N enhances the numerical stability of the optimisation algorithm.

In a similar manner, the gradient also scales linearly with the number of individuals N. Moreover, for traits that are on a larger scale (i.e., have a higher variance), one might reasonably expect a smaller response in the log-likelihood to changes in the parameters and, thus, a corresponding element of the gradient closer to zero. Therefore, MGREML takes both considerations into account in its definition of the convergence criterion.

More specifically, the convergence criterion is defined as the root mean square value of the rescaled gradient vector. Here, an element of the rescaled gradient vector is defined as the corresponding element of the true gradient divided by N (like how MGREML treats the log-likelihood) and multiplied by the standard deviation of the trait that corresponds to the given parameter. These trait-specific standard deviations are pre-computed by MGREML, before the optimisation algorithm starts. The standard deviations are calculated using OLS residuals from regressing phenotype $t = 1, \ldots, T$ on its corresponding fixed-effect covariates after the canonical transformation.

Likelihood-ratio test for nested models. Based on the model in Eq. 2, we can compare nested models using a likelihood ratio test. For example, one could compare the unconstrained model to a model without the genetic component. For two nested models, the likelihood-ratio statistic is given by

$2\left(\log l\left(\boldsymbol{\theta}_{ML}\right)-\log l\left(\boldsymbol{\theta}_{H_0}\right)\right)$, with $\boldsymbol{\theta}_{ML}$ the parameters in the unconstrained model and $\boldsymbol{\theta}_{H_0}$ the parameters in the restricted model. The test statistic follows a $\chi^2(k)$ distribution, where k is the number of coefficients that is free in the unconstrained model but constrained to zero in the (restricted) null model.

Supplementary Note 2

Supplementary Table 1 provides an overview of the data fields in the UK Biobank used to construct the traits analysed in this study.

Supplementary Table 1: Overview of the traits analysed in this study, in alphabetical order: Trait, trait description, measurement unit, and UK Biobank data fields used to construct the trait.

Trait	Trait description	Measurement unit	UK Biobank data field
BMI	Body mass index (logarithm)	Kg/m^2	21001
Depression score	First principal component of depression intensity and frequency (logarithm)	NA	2050, 2060, 4609, 4620, 5375, 5386, 2090, 2100
Drinking	Alcoholic drinks consumed per week (logarithm)	Number of units of alcohol per week	1558, 1568, 1578, 1588, 1598, 1608, 4407, 4418, 4429, 4440, 4451, 4462, 5364
Educational attainment	Highest self-reported schooling degree converted to US-schooling year equivalents using ISCED categories	years	6138
Grey matter in amygdala	Volume of grey matter in amygdala (left+right)	mm^3	25888, 25889
Grey matter in angular gyrus	Volume of grey matter in angular gyrus	mm^3	25822, 25823
Grey matter in brain-stem	Volume of grey matter in brain-stem	mm^3	25892
Grey matter in caudate	Volume of grey matter in caudate (left+right)	mm^3	25880, 25881
Grey matter in central opercular cortex	Volume of grey matter in central opercular cortex (left+right)	mm^3	25864, 25865
Grey matter in cingulate gyrus, (ad)	Volume of grey matter in cingulate gyrus, anterior division (left+right)	mm^3	25838, 25839
Grey matter in cingulate gyrus, (pd)	Volume of grey matter in cingulate gyrus, posterior division (left+right)	mm^3	25840, 25841
Grey matter in crus I cerebellum	Volume of grey matter in crus I cerebellum (left+right)	mm^3	25900, 25902

Trait	Trait description	Measurement unit	UK Biobank data field
Grey matter in crus I cerebellum)	Volume of grey matter in crus I cerebellum (vermis)	mm^3	25901
Grey matter in crus II cerebellum	Volume of grey matter in crus II cerebellum (vermis)	mm^3	25904
Grey matter in crus II cerebellum	Volume of grey matter in crus II cerebellum (left+right)	mm^3	25903, 25905
Grey matter in cuneal cortex	Volume of grey matter in cuneal cortex (left+right)	mm^3	25844, 25845
Grey matter in frontal medial cortex	Volume of grey matter in frontal medial cortex (left+right)	mm^3	25830, 25831
Grey matter in frontal operculum cortex	Volume of grey matter in frontal operculum cortex (left+right)	mm^3	25862, 25863
Grey matter in frontal orbital cortex	Volume of grey matter in frontal orbital cortex (left+right)	mm^3	25846, 25847
Grey matter in frontal pole	Volume of grey matter in frontal pole (left+right)	mm^3	25782, 25783
Grey matter in Heschl's gyrus	Volume of grey matter in Heschl's gyrus (includes H1 and H2) (left+right)	mm^3	25870, 25871
Grey matter in hippocampus	Volume of grey matter in hippocampus (left+right)	mm^3	25886, 25887
Grey matter in I-IV cerebellum	Volume of grey matter in I-IV cerebellum (left+right)	mm^3	25893, 25894
Grey matter in inferior frontal gyrus, po	Volume of grey matter in inferior frontal gyrus, pars opercularis (left+right)	mm^3	25792, 25793
Grey matter in inferior frontal gyrus, pt	Volume of grey matter in inferior frontal gyrus, pars triangularis (left+right)	mm^3	25790, 25790
Grey matter in inferior temporal gyrus, (tp)	Volume of grey matter in inferior temporal gyrus, temporooccipital part (left+right)	mm^3	25812, 25813
Grey matter in inferior temporal gyrus, (ad)	Volume of grey matter in Inferior temporal gyrus, anterior division (left+right)	mm^3	25808, 25808
Grey matter in inferior temporal gyrus, (pd)	Volume of grey matter in inferior temporal gyrus, posterior division (left+right)	mm^3	25810, 25811

Trait	Trait description	Measurement unit	UK Biobank data field
Grey matter in insular cortex	Volume of grey matter in insular cortex (left+right)	mm^3	25784, 25785
Grey matter in intracalcarine cortex	Volume of grey matter in intracalcarine cortex (left+right)	mm^3	25828, 25829
Grey matter in IX cerebellum	Volume of grey matter in IX cerebellum (left+right)	mm^3	25915, 25917
Grey matter in juxtapositional lobule cortex	Volume of grey matter in juxtapositional lobule cortex (formerly supplementary motor cortex) (left+right)	mm^3	25832, 25833
Grey matter in lateral occipital cortex, (id)	Volume of grey matter in lateral occipital cortex, inferior division (left+right)	mm^3	25826, 25827
Grey matter in lateral occipital cortex, (sd)	Volume of grey matter in lateral occipital cortex, superior division (left+right)	mm^3	25824, 25825
Grey matter in lingual gyrus	Volume of grey matter in lingual gyrus (left+right)	mm^3	25852, 25853
Grey matter in middle frontal gyrus	Volume of grey matter in middle frontal gyrus (left+right)	mm^3	25788, 25789
Grey matter in middle temporal gyrus, (tp)	Volume of grey matter in middle temporal gyrus, temporooccipital part (left+right)	mm^3	25806, 25807
Grey matter in middle temporal gyrus, (ad)	Volume of grey matter in middle temporal gyrus, anterior division (left+right)	mm^3	25802, 25803
Grey matter in middle temporal gyrus, (pd)	Volume of grey matter in middle temporal gyrus, posterior division (left+right)	mm^3	25804, 25805
Grey matter in occipital fusiform gyrus	Volume of grey matter in occipital fusiform gyrus (left+right)	mm^3	25860, 25861
Grey matter in occipital pole	Volume of grey matter in occipital pole (left+right)	mm^3	25876, 25877
Grey matter in pallidum	Volume of grey matter in pallidum (left+right)	mm^3	25884, 25884
Grey matter in paracingulate gyrus	Volume of grey matter in paracingulate gyrus (left+right)	mm^3	25836, 25837
Grey matter in parahippocampal gyrus, (ad)	Volume of grey matter in parahippocampal gyrus, anterior division (left+right)	mm^3	25848, 25849

Trait	Trait description	Measurement unit	UK Biobank data field
Grey matter in parahippocampal gyrus, (pd)	Volume of grey matter in parahippocampal gyrus, posterior division (left+right)	mm^3	25850, 25851
Grey matter in parietal operculum cortex	Volume of grey matter in parietal operculum cortex (left+right)	mm^3	25866, 25867
Grey matter in planum polare	Volume of grey matter in planum polare (left+right)	mm^3	25868, 25869
Grey matter in planum temporale	Volume of grey matter in planum temporale (left+right)	mm^3	25872, 25783
Grey matter in postcentral gyrus	Volume of grey matter in postcentral gyrus (left+right)	mm^3	25814, 25815
Grey matter in precentral gyrus	Volume of grey matter in precentral gyrus (left+right)	mm^3	25794, 25795
Grey matter in precuneous cortex	Volume of grey matter in precuneous cortex (left+right)	mm^3	25842, 25843
Grey matter in putamen	Volume of grey matter in putamen (left+right)	mm^3	25882, 25883
Grey matter in subcallosal cortex	Volume of grey matter in subcallosal cortex (left+right)	mm^3	25834, 25835
Grey matter in superior frontal Gyrus	Volume of grey matter in superior frontal gyrus (left)	mm^3	25786
Grey matter in superior parietal Lobule	Volume of grey matter in superior parietal lobule (left+right)	mm^3	25816, 25817
Grey matter in superior temporal gyrus, (ad)	Volume of grey matter in superior temporal gyrus, anterior division (left+right)	mm^3	25798, 25799
Grey matter in superior temporal gyrus, (pd)	Volume of grey matter in superior temporal gyrus, posterior division (left+right)	mm^3	25800, 25801
Grey matter in supracalcarine cortex	Volume of grey matter in supracalcarine cortex (left+right)	mm^3	25874, 25875
Grey matter in supramarginal gyrus, (ad)	Volume of grey matter in supramarginal gyrus, anterior division (left+right)	mm^3	25818, 25819
Grey matter in supramarginal gyrus, (pd)	Volume of grey matter in supramarginal gyrus, posterior division (left+right)	mm^3	25820, 25821

Trait	Trait description	Measurement unit	UK Biobank data field
Grey matter in temporal fusiform cortex, (ad)	Volume of grey matter in temporal fusiform cortex, anterior division (left+right)	mm^3	25854, 25855
Grey matter in temporal fusiform cortex, (pd)	Volume of grey matter in temporal fusiform cortex, posterior division (left+right)	mm^3	25856, 25857
Grey matter in temporal occipital fusiform cortex	Volume of grey matter in temporal occipital fusiform cortex (left+right)	mm^3	25858, 25859
Grey matter in temporal pole	Volume of grey matter in temporal pole (left+right)	mm^3	25796, 25797
Grey matter in thalamus	Volume of grey matter in thalamus (left+right)	mm^3	25878, 25879
Grey matter in V cerebellum	Volume of grey matter in V cerebellum (left+right)	mm^3	25895, 25896
Grey matter in ventral striatum	Volume of grey matter in ventral striatum (left+right)	mm^3	25890, 25891
Grey matter in VI cerebellum	Volume of grey matter in VI cerebellum (left+right)	mm^3	25897, 25899
Grey matter in VI cerebellum)	Volume of grey matter in VI cerebellum (vermis)	mm^3	25898
Grey matter in VIIb cerebellum	Volume of grey matter in VIIb cerebellum (left+right)	mm^3	25906, 25908
Grey matter in VIIb cerebellum)	Volume of grey matter in VIIb cerebellum (vermis)	mm^3	25907
Grey matter in VIIIa cerebellum	Volume of grey matter in VIIIa cerebellum (left+right)	mm^3	25909, 25911
Grey matter in VIIIa cerebellum)	Volume of grey matter in VIIIa cerebellum (vermis)	mm^3	25910
Grey matter in VIIIb cerebellum	Volume of grey matter in VIIIb cerebellum (left+right)	mm^3	25912, 25914
Grey matter in VIIIb cerebellum)	Volume of grey matter in VIIIb cerebellum (vermis)	mm^3	25913
Grey matter in X cerebellum	Volume of grey matter in X cerebellum (left+right)	mm^3	25918, 25920
Grey matter in X cerebellum)	Volume of grey matter in X cerebellum (vermis)	mm^3	25919
IQ	Standardised fluid intelligence score	correct-answers	20016, 20191

Trait	Trait description	Measurement unit	UK Biobank data field
Neuroticism	Neuroticism standardised score	NA	1920, 1930, 1940, 1950, 1960, 1970, 1980, 1990, 2000, 2010, 2020, 2030
Reaction time	Standardised reaction time	milliseconds	20023
Standing height	Standing height	cm	50
Subjective well-being	Subjective well-being: In general how happy are you? (Average value over time)	NA	4526, 20458
Visual memory	Standardised visual memory score (logarithm)	NA	399, 20132
Volume of brain	Volume of brain, grey+white matter	mm^3	25010

In our analysis sample, we removed individuals with brain diseases or surgical brain damage. Supplementary Table 2 provides an overview of the brain diseases and ICD10 codes used to make these exclusions.

As robustness check, to verify that our results are not merely a reflection of the physical proximity of brain regions, we regressed the estimated genetic correlations on the physical distance between the different brain regions (see Supplementary Note 3). Supplementary Table 3 provides an overview of the MNI (Montreal Neurological Institute) coordinates used to calculate the distances between regions.

Supplementary Table 2: Overview of data fields in the UK Biobank used to exclude individuals with brain diseases or surgical brain damage.

Trait	UK Biobank data field	ICD10 code
Dementia or Alzheimer's disease	1263	F01, F02, G30
Parkinson's disease	1262	G20, G21
Chronic degenerative neurological	1258	G23, G31, G32
Guillain-Barré syndrome	1256	G610
Multiple Sclerosis	1261	G35
Other demyelinating disease	1397	G37
Stroke or ischaemic stroke	1081	G463, G464, I64, I694
Brain cancer	1031	C70, C71, D33
Brain haemorrhage	1491	I60, I61, I62, I691, I692, I693
Brain/intracranial abscess	1245	G060, G07
Cerebral aneurysm	1425	I671, Q282, Q283
Cerebral palsy	1433	G80, A521, A504, I64
Encephalitis	1246	A83, A86, B011, B020, B262, A85, B004, B582, A84, B050, B941, G04, A321, G05
Epilepsy	1264	G40, F803
Head injury	1266	S07, T040
Infections of the nervous system	1244	A80, A81, A82, A83, A84, A85, A86, A87, A88, A89
Ischaemic stroke	1583	G45
Meningeal cancer	1031	C70, C793
Meningioma (benign)	1659	D33, D32
Meningitis	1247	G03, A170, A171, A203, G01, G02, G00, G07
Motor neuron disease (ALS)	1259	G122
Neurological injury / trauma	1240	
Spina bifida	1524	Q05, Q760
Subdural haematoma	1083	P100
Subarachnoid haemorrhage	1086	I60, S066, P103
Transient ischaemic attack	1082	G45

Supplementary Table 3: MNI coordinates of regions of interest.

Grey matter area	x-coordinate	y-coordinate	z-coordinate
Central opercular cortex	55	-5	9
Planum temporale	57	-19	5
Middle temporal gyrus, anterior division	56	-1	-19
Frontal pole	29	59	1
Precuneous cortex	13	-61	34
Heschls gyrus (includes H1 and H2)	47	-22	7
Paracingulate gyrus	-1	37	31
Juxtapositional lobule cortex (formerly Supplementary M	5	-3	63
Parietal operculum cortex	47	-27	31
Inferior temporal gyrus, posterior division	47	-21	-29
Cuneal cortex	21	-68	24
Middle temporal gyrus, posterior division	50	-23	-11
Superior temporal gyrus, posterior division	66	-20	3
Middle frontal gyrus	40	31	32
Superior temporal gyrus, anterior division	57	-10	-4
Planum polare	44	-10	-11
Insular cortex	41	-10	2
Lateral occipital cortex, inferior division	41	-77	-10
Precentral gyrus	48	-6	48
Cingulate gyrus, posterior division	9	-34	35
Cingulate gyrus, anterior division	9	18	27
Frontal operculum cortex	44	18	2
Superior frontal gyrus	14	18	59
Frontal orbital cortex	22	24	-21
Inferior temporal gyrus, temporooccipital part	51	-50	-20
Inferior temporal gyrus, anterior division	44	0	-42
Subcallosal cortex	8	17	-13
Supramarginal gyrus, anterior division	57	-30	54
Inferior frontal gyrus, pars opercularis	57	15	11
Supracalcarine cortex	23	-63	14
Inferior frontal gyrus, pars triangularis	49	27	14
Frontal medial cortex	41	24	35
Lateral occipital cortex, superior division	35	-70	37
Temporal fusiform cortex, posterior division	34	-32	-24
Temporal pole	34	16	-36
Middle temporal gyrus, temporooccipital part	65	-50	0
Temporal fusiform cortex, anterior division	27	-2	-44
Postcentral gyrus	42	-31	59
Occipital pole	23	-102	4
Angular gyrus	51	-53	33
Superior parietal lobule	30	-49	57
Supramarginal gyrus, posterior division	54	-39	36
Intracalcarine cortex	17	-77	5
Vermis crus I cerebellum	3	-71	-33
Parahippocampal gyrus, anterior division	17	-8	-29
Occipital fusiform gyrus	24	-77	-19
Parahippocampal gyrus, posterior division	24	-27	-18
Lingual gyrus	13	-59	-3
Hippocampus	-26	-19	-17
Temporal occipital fusiform cortex	35	-48	-16
Pallidum	22	6	-2
Amygdala	27	5	-17
Caudate	12	11	10
Thalamus	15	-19	0
Putamen	25	1	-3
Vermis crus II cerebellum	3	-71	-34
Ventral striatum	20	-4	-5
Crus I cerebellum	45	-52	-35
X cerebellum	2	-50	-35
Brain-stem	1	-25	-33
Vermis VI cerebellum	3	-71	-29
Crus II cerebellum	46	-52	-46
VIIb cerebellum	7	-69	-32
Vermis VIIb cerebellum	3	-71	-33
Vermis X cerebellum	3	-68	-41
VIIIb cerebellum	30	-56	-53
I-IV cerebellum	9	-45	-16
Vermis VIIIb cerebellum	3	-71	-35
IX cerebellum	10	-47	-52
VI cerebellum	28	-57	-25
V cerebellum	11	-57	-14
VIIIa cerebellum	30	-65	-53
Vermis VIIIa cerebellum	3	-71	-32
Vermis IX cerebellum	5	-68	-35

Supplementary Note 3

Runtime analyses. Panels (a) and (c) in Supplementary Figure 1 provide the results of a runtime comparison between MGREML in default mode and in pairwise bivariate mode. Although pairwise bivariate GREML is certainly not new (e.g., Lee et al., 2012), we implemented a pairwise mode in MGREML to facilitate a fair comparison between the pairwise and multivariate approach. This approach also allows the pairwise approach to take full advantage of the canonical transformation, the application of the commutation matrix, efficient control for population stratification, and other aspects that are all efficiently implemented in MGREML. The results presented are based solely on optimising the MGREML function. That is, the AI matrix and standard errors are not computed in this application of MGREML to simulated data.

The data simulated here are on traits with $h^2_{\text{SNPs}} = 25\%$, with a number of SNPs that equals the sample size N, and with no genetic correlations and no environmental correlations between traits. The results show that, given a number of individuals (N) and traits (T), MGREML is computationally faster than pairwise bivariate GREML in all scenarios considered here. However, we observe in panel (a) that the disparity between the two approaches decreases when sample size, N, increases. On the other hand, in terms of the number of traits, T, panel (c) shows that the gap only widens when more traits are analysed. Finally, we observe that even for as many as 200 traits, MGREML in default mode requires only 65 minutes and MGREML in pairwise mode only requires four hours to complete.

Memory usage. In addition to runtime, panels (b) and (d) of Supplementary Figure 1 show memory usage in GB. When fixing $N = 20,000$ and varying T in panel (b), we find that for up to 100 traits, memory usage hardly responds to T. In these scenarios, memory usage is about 12GB. However, when moving beyond 100 traits the memory required by MGREML in default mode starts to increase rapidly. The reason for this pattern is that for $T \leq 100$, at $N = 20,000$, the GRM and its eigenvalue decomposition are the largest objects that need to be held in the most memory-intensive step of the MGREML analysis. However, as T goes beyond 100, the approximation of the inverse of the Hessian, used by the BFGS algorithm, starts dominating memory usage.

For instance, when $T = 1,000$ traits, there are $1,000 \times 1,001 \approx 10^6$ parameters in the model. Thus, the approximate inverse Hessian in that case is a full matrix with about 10^6 rows and 10^6 columns. Such a matrix is memory-wise clearly far from feasible. Therefore, a future development of MGREML for enhanced scalability might lie in the usage of a so-called limited-memory BFGS algorithm.

Finally, as visible from panel (d), at a logarithmic scale memory usage increases slowly with N for both the default mode and the pairwise bivariate mode. This observation is in line with our theoretical expectation that for typical values of N and T, holding the eigenvalue decomposition and/or the GRM in memory (prior to the MGREML analysis itself) is memory-wise the most intensive step (requiring $O(N^2)$ memory).

Overall, we conclude that for reasonable values of T, MGREML in default mode and pairwise mode have a very comparable performance in terms of memory usage. Moreover, in terms of CPU time, the default mode strictly outperforms the pairwise model. Yet, for extremely large T (e.g., more than a hundred traits), users of MGREML may consider using the pairwise mode instead of the default mode. However, in pairwise mode, MGREML can no longer guarantee that the resulting genetic correlation matrix is positive semi-definite.

Comparisons with alternative individual-level data methods. To further investigate the computational gains afforded by MGREML, we compare our method to publicly available methods that are also tailored towards the same type of analyses. In particular, we consider GCTA (Yang et al., 2011), MTG2 (Lee and Van der Werf, 2016), GEMMA (Zhou and Stephens, 2012), WOMBAT (Meyer, 2007), and BOLT-REML (Loh et al., 2015). We also briefly discuss ASReml (Gilmour, 1997) vis-à-vis MGREML. We use our empirical application ($N = 20,190$) as an input to benchmark our method against GCTA, MTG2, GEMMA, WOMBAT, and BOLT-REML.

We start with a bivariate analysis ($T = 2$), and then increase to $T = 10$. After this, we further increase T in steps of 10, until we exhaust the full set of 86 traits. Thus, we consider $T = 2, 10, 20, 30, \ldots, 80, 86$. For each of these values of T, we allot each method exactly 24 hours of CPU time on the same type of machine (i.e., a machine with 24 cores, a clock speed of 2.6GHz, and 64GB of memory).

In these analyses, GCTA runs out of memory even for $T = 2$ traits, without controlling for any fixed-effect covariates. MTG2, on the other hand, can easily handle a bivariate analysis of this scale. Overall, MTG2 can handle at most 30 traits on our computing infrastructure. For this analysis, MTG2 requires 51 minutes to compute the eigenvalue decomposition of the GRM and a further 16 minutes to estimate the multivariate model. Notably, we run MTG2 without correcting for the first 20 principal components from the genetic data. The reason for this omission is that MTG2's computational complexity is $O(NT^6)$ per iteration when the number of unique fixed-effect covariates is $O(1)$ (see Lee and Van der Werf, 2016, Supplementary Data, Page 5), which becomes prohibitively complex for high T (even for $T = 30$). When $T = 40$, MTG2 runs out of memory. Furthermore, MTG2 requires fully balanced data to use its fast algorithm, whereas MGREML

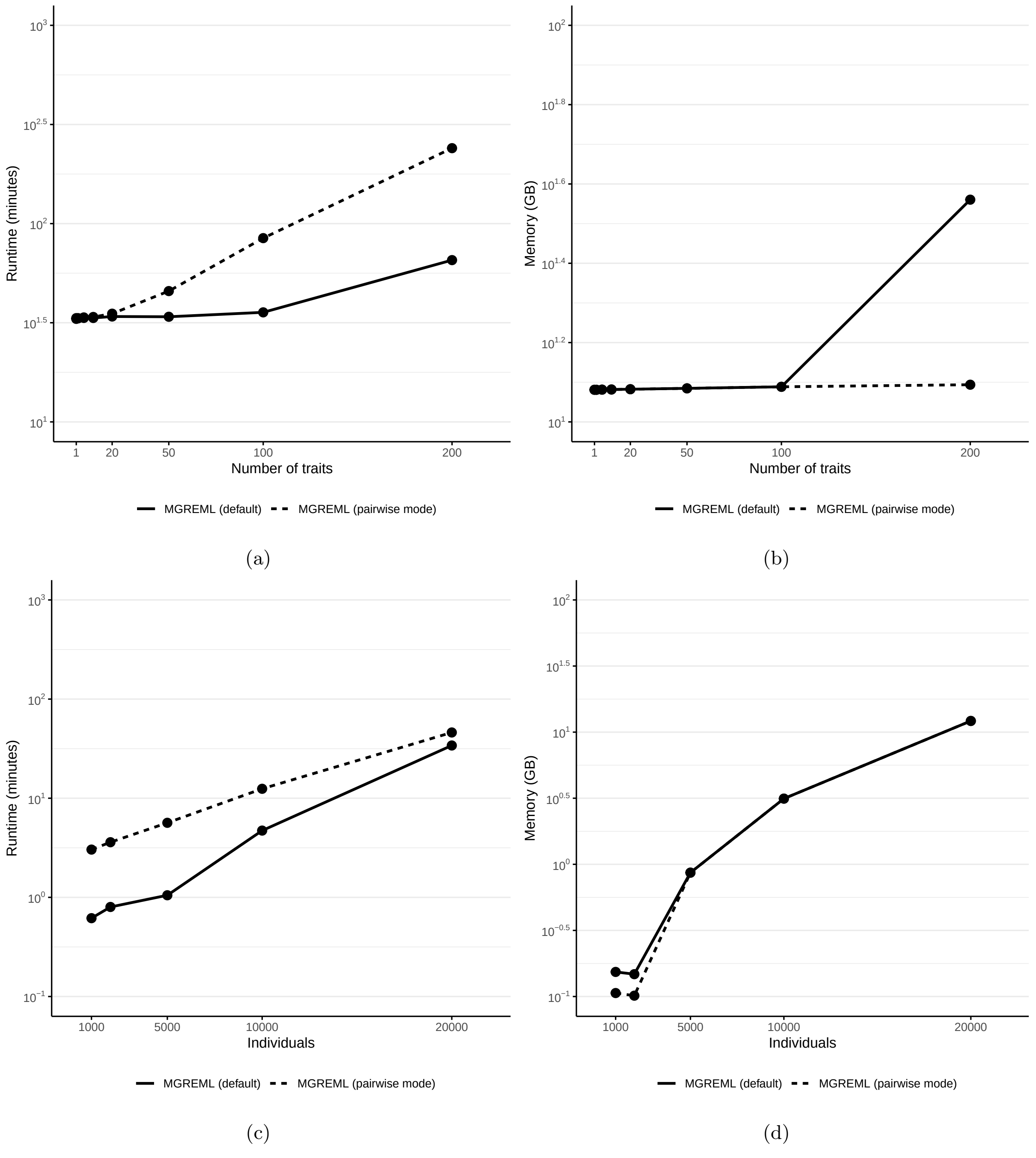

Supplementary Figure 1: Runtime (in minutes; first column) and memory usage (in GB; second column) of MGREML applied to simulated data (i) in default mode (solid lines) and (ii) in pairwise bivariate mode (dashed lines), as function of the number of traits T for sample size $N = 20,000$ (first row) and as function of sample size N for number of traits $T = 50$ (second row).

can deal with small amounts of missingness.

GEMMA, like MTG2, is able to handle at most $T = 30$ traits. For $T = 40$, the method does not converge within 24 hours. For $T = 50$, the method crashes immediately due to insufficient memory. We do note here, that MTG2 is considerably faster than GEMMA for estimating the variance components when $N = 20,190$ and $T = 30$. Whereas GEMMA takes hours to estimate the model for $T = 30$, MTG2 is able to estimate the model in 16 minutes once the eigenvalue decomposition of the GRM has been computed.

When using WOMBAT, even a simple bivariate analysis requires more than four hours of CPU time. Hence, even the simple approach of aggregating results from $[T(T-1)]/2$ pairwise bivariate analyses would imply thousands of hours of runtime, when considering our full set of $T = 86$ traits. Moreover, for $T = 10$ traits, the analysis fails due to its severe memory requirements. We conclude that WOMBAT has trouble with a dense similarity matrix, as this software is optimised for cattle data in which one effectively looks at expected relatedness (as implied by the pedigree). In such data, there is a lot of sparsity in the relatedness matrix. For applications in human genetics, where the GRM is constructed using molecular genetic data, such sparsity is completely absent.

Finally, we compare MGREML to BOLT-REML. For a simple bivariate analysis, BOLT-REML takes over two hours to estimate the model. Hence, also here, the simple approach of aggregating results from pairwise bivariate analyses would imply thousands of hours of runtime, when considering our full set of 86 traits. Moreover, for $T = 10$ traits, BOLT-REML does not converge within 24 hours.

The commercial nature of ASReml is a significant impediment for widespread usage, especially in (behaviour) genetics and related fields where researchers are used to work with freely and publicly available, open-source software. Also, as with WOMBAT, ASReml, by design, is more tailored towards an expected relatedness matrix that is implied by a given pedigree file. Again, a relatedness matrix based on molecular genetic data is a dense matrix. Moreover, like GCTA and MTG2, ASReml relies strongly on the AI matrix to maximise the log-likelihood function. Here, we stress that the AI matrix has an irreducible computational complexity of $O(NT^4)$ for a fully saturated model. Thus, at the scale of $T = 86$, methods such as ASReml simply cannot compete with the BFGS algorithm implemented in MGREML.

Comparison with summary statistic methods. We investigated the statistical efficiency of MGREML by comparing the standard errors of the estimated genetic correlations (Supplementary Data 8) to the standard errors obtained by using bivariate LD-score regression (LDSC) (Bulik-Sullivan et al., 2015) and

SumHer (Speed and Balding, 2019) to estimate genetic correlations. For this comparison, we used PLINK 1.9 (Chang et al., 2015) to run a GWAS for each of the 86 traits using the 1,384,830 SNPs that are also used for constructing the GRM, with the same covariates as those used in the MGREML analyses. The summary statistics of these GWASs were used to compute genetic correlations in a pairwise fashion using LDSC v1.0.1 and SumHer (as implemented in LDAK 5.1).

For the LDSC analyses, we computed genetic correlations using two reference samples: The European individuals of the 1000 Genomes data (McVean et al., 2012) and the European individuals of the UK Biobank (as provided by the Pan-UKB Team, 2020). While the first reference sample is more commonly used in the literature, the second reference sample is likely to reflect the LD structure of our analysis sample better. For that reason, while the LDSC results somewhat depend on the reference sample used, in the main text we report the results obtained using the UK Biobank reference sample. The estimated heritabilities, genetic correlations and corresponding standard errors are available in Supplementary Data 4 and Supplementary Data 5. The results show that the heritability estimates obtained with MGREML and LDSC are strongly correlated ($\rho = 0.95$ when using the 1000Genomes reference sample, and $\rho = 0.93$ when using the UK Biobank reference sample). The same holds for the genetic correlations ($\rho = 0.90$ and $\rho = 0.88$, these values are based on the genetic correlations below the diagonal). Importantly, the standard errors obtained with MGREML are, compared to the LDSC results, on average smaller for both the heritability estimates (37.6% and 32.7%) and the genetic correlation estimates (45.2% and 50.6%).

The heritabilities, genetic correlations and corresponding standard errors as estimated using SumHer can be found in Supplementary Data 7. Here, we used the recommended LDAK-Thin Model pre-computed tagging files for the GBR population in UKB that are publicly available on the LDAK website (see: `http://dougspeed.com/pre-computed-tagging-files/`). The results show that there is again a strong correlation between heritability estimates from MGREML and SumHer ($\rho = 0.94$). The genetic correlations are also highly correlated ($\rho = 0.89$). The standard errors obtained using MGREML are smaller than those obtained using SumHer for both the heritability estimates (46.2%) as well as the genetic correlations (46.1%).

The comparison of the results obtained with MGREML on the one hand and those obtained with LDSC and SumHer on the other hand illustrate again the statistical efficiency of individual-level data methods. While all three methods provide similar estimates, MGREML provides the most precise estimates (i.e., those with the smallest standard errors).

Construction of the genomic-relatedness matrix (GRM). Various tools, such as GCTA (Yang et al., 2011) and LDAK (Speed et al., 2012), can be used to construct the GRM which is used as input for an MGREML analysis. Importantly, the GRM as calculated by GCTA assumes that all SNPs explain the same proportion of phenotypic variance (irrespective of, e.g., a SNP's minor allele frequency and the region it is located). Therefore, other perhaps more realistic assumptions about the distribution of SNP effects have been proposed and utilized in tools such as LDAK. In the empirical application considered in this study, however, using the GRM as calculated by LDAK (based on the so-called LDAK-Thin Model) yields a slightly poorer fit in terms of the log-likelihood compared to using the GRM as calculated by GCTA. Assessing the significance of this difference in fit is not straightforward, because these models are not nested with respect to each other. In our empirical application, the estimated SNP-based heritabilities and genetic correlations are highly similar when using these two differently calculated GRMs (Supplementary Data 8).

Correcting genetic correlations for physical proximity. To verify that our results are not merely a reflection of the physical proximity of brain regions, we regressed the estimated genetic correlations on the physical distance between the different brain regions. To compute distances between brain regions, coordinates are used from the Harvard-Oxford cortical brain area maps, the JHU atlas for the subcortical structures, and the cerebellar regions from the SUIT cerebellum atlas.

More specifically, we compute squared Euclidean distances using the MNI coordinates shown in Supplementary Table 3, denoted by d. Next, we square the estimated genetic correlations, and we compute the logit transformation of these squared correlations, denoted by s, yielding a measure in $\mathbb{R}$ that is low for genetic correlations close to zero and high for genetic correlations close to $+1$ or -1. We compute the logarithm of $d+1$ to make the distances more normally distributed. The increment by one is needed, as the distance between two specific regions can be zero (resulting from the granularity of the MNI coordinates), *viz.*, Vermis VIIb Cerebellum and Vermis Crus I Cerebellum.

We regress s on $\log(1+d)$ and an intercept. Using the regression estimates, we compute fitted values $\widehat{s}$. Finally, we cast these fitted values to predicted absolute genetic correlations by taking the square root of the logistic function of $\widehat{s}$, and we compute the squared correlation between the absolute value of the estimated genetic correlations and the absolute genetic correlations as predicted by this regression model. Here, we find that physical distance explains 17.4% of the variation in the absolute value of genetic correlations between relative grey matter volume in regions of interest, as estimated by MGREML.

Supplementary Note 4

In this section, we describe the analysis pipeline used to obtain the empirical results as reported in the main text:

1. Restrict to individuals of European ancestry.
2. Exclude the individuals with brain damage (see Supplementary Table 2 for UK Biobank data cells used to exclude these individuals).
3. Restrict dataset to directly genotyped HapMap3 SNPs + SNPs with imputation quality of 0.9 or higher.
    - This step leaves a total of $1,796,892$ directly genotyped SNPs / SNPs with high imputation quality.
4. Apply regular quality control on SNP data: minor allele frequency (MAF) < 0.01, missingness per individual (MIND) < 0.05, missingness per SNP (GENO) < 0.05, Hardy-Weinberg equilibrium (HWE) p-value < 0.001.
    - This step leaves a total of $1,582,522$ SNPs.
5. Drop long-range LD (linkage disequilibrium) regions, following Linnér et al. (2019).
    - This step leaves a total of $1,384,830$ SNPs.
6. Construct GRM, and apply relatedness cutoff of 0.025 using PLINK (Chang et al., 2015).
    - This step leaves a total of $N = 37,392$ individuals.
7. Curate phenotype data (including construction of genotyping platform dummy variable, which we use as control variable for each trait)
    - Exclude individuals with missing phenotypes: Our choice for fully balanced data leaves us with $N = 20,190$ individuals.
    - Residualise the 74 brain volume phenotypes and the 2 global measures of brain volume with respect to the following covariates: age, age^2, sex, sex$\times$age, head size, head motion while resting, head motion in task, date, date^2, UK Biobank Assessment Centre, interactions between the UK Biobank Assessment Centre and all preceding covariates, and a constant.

- Residualise the 10 other traits with respect to the following covariates: age, age^2, sex, sex×age, head size, date, date^2, UK Biobank Assessment Centre, interactions between the UK Biobank Assessment Centre and all preceding covariates, and a constant.
  - For IQ, when residualising, instead of age, we use age at the moment of the IQ assessment and we, additionally, control for dummy variables for the number of IQ measurements used to construct this variable.

8. Run MGREML (with adjustment for the first 20 lead PCs).

Supplementary References

Bulik-Sullivan, B. K., , Loh, P.-R., Finucane, H. K., Ripke, S., Yang, J., Patterson, N., Daly, M. J., Price, A. L., and Neale, B. M. (2015). LD Score regression distinguishes confounding from polygenicity in genome-wide association studies. *Nature Genetics*, 47(3):291–295.

Casella, G. and Searle, S. R. (1985). On a matrix identity useful in variance component estimation. Biometrics Unit Technical Reports; Number BU-875-M.

Chang, C. C., Chow, C. C., Tellier, L. C. A. M., Vattikuti, S., Purcell, S. M., and Lee, J. J. (2015). Second-generation `PLINK`: rising to the challenge of larger and richer datasets. *GigaScience*, 4:1–16.

Ducrocq, V. and Chapuis, H. (1997). Generalizing the use of the canonical transformation for the solution of multivariate mixed model equations. *Genetics Selection Evolution*, 29:205–224.

Gilmour, A. (1997). `ASREML` for testing fixed effects and estimating multiple trait variance components. *Proceedings of the Association for the Advancement of Animal Breeding and Genetics*, 12:386–390.

Gilmour, A. R., Thompson, R., and Cullis, B. R. (1995). Average information REML: an efficient algorithm for variance parameter estimation in linear mixed models. *Biometrics*, 51:1440–1450.

Harville, D. A. (1977). Maximum likelihood approaches to variance component estimation and to related problems. *Journal of the American Statistical Association*, 72(358):320–338.

Lee, S., Yang, J., Goddard, M., Visscher, P., and Wray, N. (2012). Estimation of pleiotropy between complex diseases using single-nucleotide polymorphism-derived genomic relationships and restricted maximum likelihood. *Bioinformatics*, 28(19):2540–2542.

Lee, S. H. and Van der Werf, J. H. (2016). `MTG2`: an efficient algorithm for multivariate linear mixed model

analysis based on genomic information. *Bioinformatics*, 32:1420–1422.

Linnér, R. K., Biroli, P., Kong, E., Meddens, S. F. W., Wedow, R., Fontana, M. A., Lebreton, M., Tino, S. P., Abdellaoui, A., Hammerschlag, A. R., et al. (2019). Genome-wide association analyses of risk tolerance and risky behaviors in over 1 million individuals identify hundreds of loci and shared genetic influences. *Nature Genetics*, 51(2):245–257.

Loh, P.-R., Bhatia, G., Gusev, A., Finucane, H. K., Bulik-Sullivan, B. K., Pollack, S. J., de Candia, T. R., Lee, S. H., Wray, N. R., Kendler, K. S., et al. (2015). Contrasting genetic architectures of schizophrenia and other complex diseases using fast variance-components analysis. *Nature Genetics*, 47:1385–1392.

McVean, G. A., Altshuler, D. M., and the 1000 Genomes Project Consortium (2012). An integrated map of genetic variation from 1,092 human genomes. *Nature*, 491:56–65.

Meyer, K. (2007). `WOMBAT`—A tool for mixed model analyses in quantitative genetics by restricted maximum likelihood (REML). *Journal of Zhejiang University Science B*, 8:815–821.

Nocedal, J. and Wright, S. (2006). *Numerical Optimization*. Springer.

Pan-UKB Team (2020). https://pan.ukbb.broadinstitute.org.

Searle, S. R., Casella, G., and McCulloch, C. E. (1992). *Variance Components*, chapter M.4, pages 451–452. John Wiley and Sons, Hoboken, New Jersey, USA.

Speed, D. and Balding, D. J. (2019). Sumher better estimates the snp heritability of complex traits from summary statistics. *Nature Genetics*, 51(2):277–284.

Speed, D., Hemani, G., Johnson, M. R., and Balding, D. J. (2012). Improved heritability estimation from genome-wide SNPs. *American Journal of Human Genetics*, 91:1011–1021.

Yang, J., Benyamin, B., McEvoy, B., Gordon, S., Henders, A., Nyholt, D., Madden, P., Heath, A., Martin, N., Montgomery, G., Goddard, M., and Visscher, P. (2010). Common SNPs explain a large proportion of the heritability for human height. *Nature Genetics*, 42:565–9.

Yang, J., Lee, S. H., Goddard, M. E., and Visscher, P. M. (2011). `GCTA`: a tool for genome-wide complex trait analysis. *American Journal of Human Genetics*, 88:76–82.

Zhou, X. and Stephens, M. (2012). Genome-wide efficient mixed-model analysis for association studies. *Nature Genetics*, 44:821–824.
