# Supplementary material for: Multivariate analysis reveals shared genetic architecture of brain morphology and human behavior
Source: Commun Biol. 2021 Oct 12;4:1180. doi: 10.1038/s42003-021-02712-y (PMC8511103; doi:10.1038/s42003-021-02712-y)
Supplement: Supplementary file 3 — Description of Additional Supplementary Files [file 42003_2021_2712_MOESM3_ESM.pdf]

Description of Additional Supplementary Files

File name: Supplementary Data 1
Description: The heritability of and genetic correlations across 86 traits in 20,190 unrelated 'white British' individuals from the UK Biobank as estimated using MGREML (relative volumes).

File name: Supplementary Data 2
Description: The heritability of and genetic correlations across 86 traits in 20,190 unrelated 'white British' individuals from the UK Biobank as estimated in a pairwise (bivariate) approach using MGREML (relative volumes). Each *h2* (and its standard errors) reflects the average estimate from 85 pairwise analyses.

File name: Supplementary Data 3
Description: The heritability of and genetic correlations across 86 traits in 20,190 unrelated 'white British' individuals from the UK Biobank as estimated using MGREML (absolute volumes).

File name: Supplementary Data 4
Description: The heritability of and genetic correlations across 86 traits in 20,190 unrelated 'white British' individuals from the UK Biobank as estimated using LDSC (relative volumes). Reference sample for LDSC: 1000 Genomes (Europeans).

File name: Supplementary Data 5
Description: The heritability of and genetic correlations across 86 traits in 20,190 unrelated 'white British' individuals from the UK Biobank as estimated using LDSC (relative volumes). Reference sample for LDSC: UK Biobank (Europeans).

File name: Supplementary Data 6
Description: The heritability of and genetic correlations across 86 traits in 20,190 unrelated 'white British' individuals from the UK Biobank as estimated using MGREML (relative volumes). LDAK-Thin GRM.

File name: Supplementary Data 7
Description: The heritability of and genetic correlations across 86 traits in 20,190 unrelated 'white British' individuals from the UK Biobank as estimated using SumHer (relative volumes). Reference sample for SumHer: UK Biobank (GBR tagging file).

File name: Supplementary Data 8
Description: The average heritability (and standard error) of 86 traits as estimated in 20,190 unrelated 'white British' individuals from the UK Biobank using different heritability models.
